# Filamentous morphology of influenza A virus confers enhanced stability in aerosols

**DOI:** 10.1038/s41467-026-73133-w

**Published:** 2026-05-15

**Authors:** Lu Liu, Ghislain Motos, Céline Terrettaz, Sarah Peterl, Josephine von Kempis, Marie O. Pohl, Umut Karakus, Elisabeth Gaggioli, Beiping Luo, Ulrich K. Krieger, Thomas Peter, Petr Chlanda, Tamar Kohn, Athanasios Nenes, Silke Stertz

**Affiliations:** 1 Institute of Medical Virology, University of Zurich, Zurich, Switzerland; 2 Laboratory of Atmospheric Processes and their Impacts, School of Architecture, Civil & Environmental Engineering, École Polytechnique Fédérale de Lausanne, Lausanne, Switzerland; 3 Laboratory of Environmental Virology, School of Architecture, Civil & Environmental Engineering, École Polytechnique Fédérale de Lausanne, Lausanne, Switzerland; 4 Department of Infectious Diseases, Virology, Heidelberg University, Heidelberg, Germany; 5 BioQuant Centre for Quantitative Biology, Heidelberg University, Heidelberg, Germany; 6 Institute for Atmospheric and Climate Science, ETH Zurich, Zürich, Switzerland; 7 Center for the Study of Air Quality and Climate Change, Foundation for Research and Technology Hellas, Patras, Greece

**Keywords:** Viral transmission, Influenza virus

## Abstract

Airborne transmission of influenza A virus (IAV) poses significant challenges to public health. However, the mechanisms governing viral inactivation in aerosols remain poorly understood. IAVs exhibit morphological variability, ranging from 100 nm spherical virions to micrometer-long filaments, depending on strain and growth conditions. Although virion morphology was shown to influence transmissibility, the mechanisms of how morphology affects airborne transmission are yet to be delineated. Here, we investigated the impact of virion shape on IAV stability in bulk solutions and an aerosol system with a focus on particles in the submicrometer range to examine how physicochemical aerosol properties, such as elevated solute concentration and low pH, affect infectivity. We show that filamentous viruses exhibit enhanced stability in aerosol particles at 80% relative humidity (RH) and in bulk solution mimicking increased salinity at this RH. Similarly, filamentous viruses exhibited slower decay under acidic conditions, both in bulk solutions and in acidified aerosol particles. Using primary human airway cultures, we further found that filamentous IAVs possess an infectivity advantage under mucosal immune pressures, including neutralizing antibodies and mucus. These results reveal that filamentous shape provides IAV with enhanced stability under diverse environmental conditions in the aerosol phase and increased infectivity in the respiratory epithelium.

## Abstract (web summary)

Liu et al. reveal that filamentous shape compared to spherical morphology provides influenza A virus with enhanced stability under diverse environmental conditions in the aerosol phase and increased infectivity in the respiratory epithelium.

## Introduction

Influenza A viruses (IAVs) are human respiratory pathogens that cause seasonal epidemics and occasionally pandemics, posing a substantial burden on health care systems with >5 million hospitalizations each year^1–3^. The difficulty in controlling IAV is largely attributed to its airborne transmission, which facilitates rapid spread^4–6^. IAVs exhibit significant morphological variability^7^ and such pleomorphism has been observed for various other airborne viruses, including respiratory syncytial virus^8^, measles virus^9^, and Newcastle disease virus^10^. While many laboratory-adapted IAV strains produce predominantly spherical virions with a diameter of 80-100 nm^11,12^, some low-passage-number isolates display a heterogeneous mix of virion shapes, including spherical, bacilliform, and particularly filamentous forms^13–17^. Recent work has also shown that experimental conditions during virus cultivation can modulate the ratio of shapes produced for each strain^18^. Filamentous virions maintain a similar diameter but can extend to lengths exceeding 250 nm, with some reaching beyond 10 μm^13,19,20^. IAV filamentous morphology has been reported to be associated with viral transmissibility in animal infection models^21,22^. However, the mechanisms by which filamentous morphology contributes to viral transmission remain unclear.

IAV can spread through infectious respiratory particles (IRPs) emitted from the respiratory tract of an infected individual during coughing, sneezing, breathing and talking^23^. Airborne transmission strongly depends on the ability of virions to retain infectivity under the harsh physicochemical conditions found in IRPs. Although significant gaps remain in our understanding of aerosol microphysics, it is proposed that high salt concentrations as a result of the reduction in water activity after exhalation, as well as changes in pH play a critical role in influencing IAV stability^24–29^. IRPs initially have high water activity (~ 0.995), resulting from equilibrium with the high RH of the respiratory tract. Upon exhalation, water evaporation causes a rise in salt concentration and a decrease in water activity until equilibrium with indoor RH is reached^24^, typically ranging between 20% and 60%^30^. The evolution of IRPs pH, however, remains a topic of active debate, and is influenced by the respiratory matrix, and the gaseous acidic and alkaline compounds partitioning between the gas phase and IRPs. A number of studies show that large IPRs (diameters several 10 µm) turn alkaline due to CO_2_ outgassing and loss of bicarbonate and stay alkaline for extended periods of time^31–33^. In our studies^28,34^, we concluded that small IPRs (diameters of a few micrometers) become slightly alkaline for only a few seconds due to initial CO_2_ evaporation but acidify strongly within minutes due to gas exchange with typical room air^35,36^, reaching pH values ≲ 4. Our Respiratory Aerosol Model (ResAM), which simulates the thermodynamics and kinetics of particles exhaled by humans, shows that the pH dynamics in IRPs varies depending on the size and matrix composition of the particles as well as the composition of the room air^28^. While it has been shown that high salinity and acidity can lead to IAV inactivation^27,28^, it is thus far unclear if different strains of IAV display differential sensitivity. In particular, the effects of viral morphology on infectivity decay in IRPs are poorly defined.

Once a given dose of infectious virus enters the respiratory tract of a new host via the airborne route, the ability of virions to withstand host defenses determines infection outcomes. Notably, filamentous IAV is prevalent in primary or low-passage human isolates but is not favored under standard in vitro tissue culture conditions, suggesting a selective advantage in vivo^12–14^. In fact, filamentous virions have been shown to display reduced sensitivity to antiviral pressures, such as neutralizing antibodies against hemagglutinin (HA) and fusion inhibitors^37^. These phenomena are linked to a larger surface with greater number of HA proteins coupled with filamentous morphology^37,38^. Additionally, polarized distribution of HA and neuraminidase (NA) on viral filaments may facilitate their directional movement through mucus, a major barrier to respiratory infection^39^. However, the precise impact of these traits on viral infectivity in vivo remains insufficiently characterized, and thus, it is unknown how these findings translate into viral fitness during airborne transmission.

Here, we aimed to understand the impact of viral morphology on IAV infectivity in the airborne transmission chain. We assessed the infectivity of differently shaped IAV in the aerosol phase and an ex vivo model of the human respiratory epithelium. We evaluated the stability of spherical versus filamentous virions by first comparing their inactivation kinetics under aerosol-mimicking harsh conditions, such as low pH, high pH and high salt concentration, in bulk solutions, followed by validation in the aerosol phase with a system that focuses on virus infectivity in aerosol particles of the submicrometer range. Our findings reveal that viral morphology influences aerosol stability. Specifically, filamentous virions demonstrate higher stability under high salinity and under low pH conditions. Furthermore, we find that filamentous virions are more resistant to neutralization by IgA and IgG antibodies targeting HA, as well as mucus secreted from differentiated primary human airway epithelial cells (hAEpCs), and we dissect their underlying mechanisms. Collectively, our findings support a model in which IAV filamentous shape confers an advantage in preserving viral infectivity under harsh environmental conditions within small IRPs and the host, ultimately enhancing airborne transmission efficiency.

## Results

### Generation and characterization of IAVs with spherical or filament-producing morphology

**Fig. 1:**
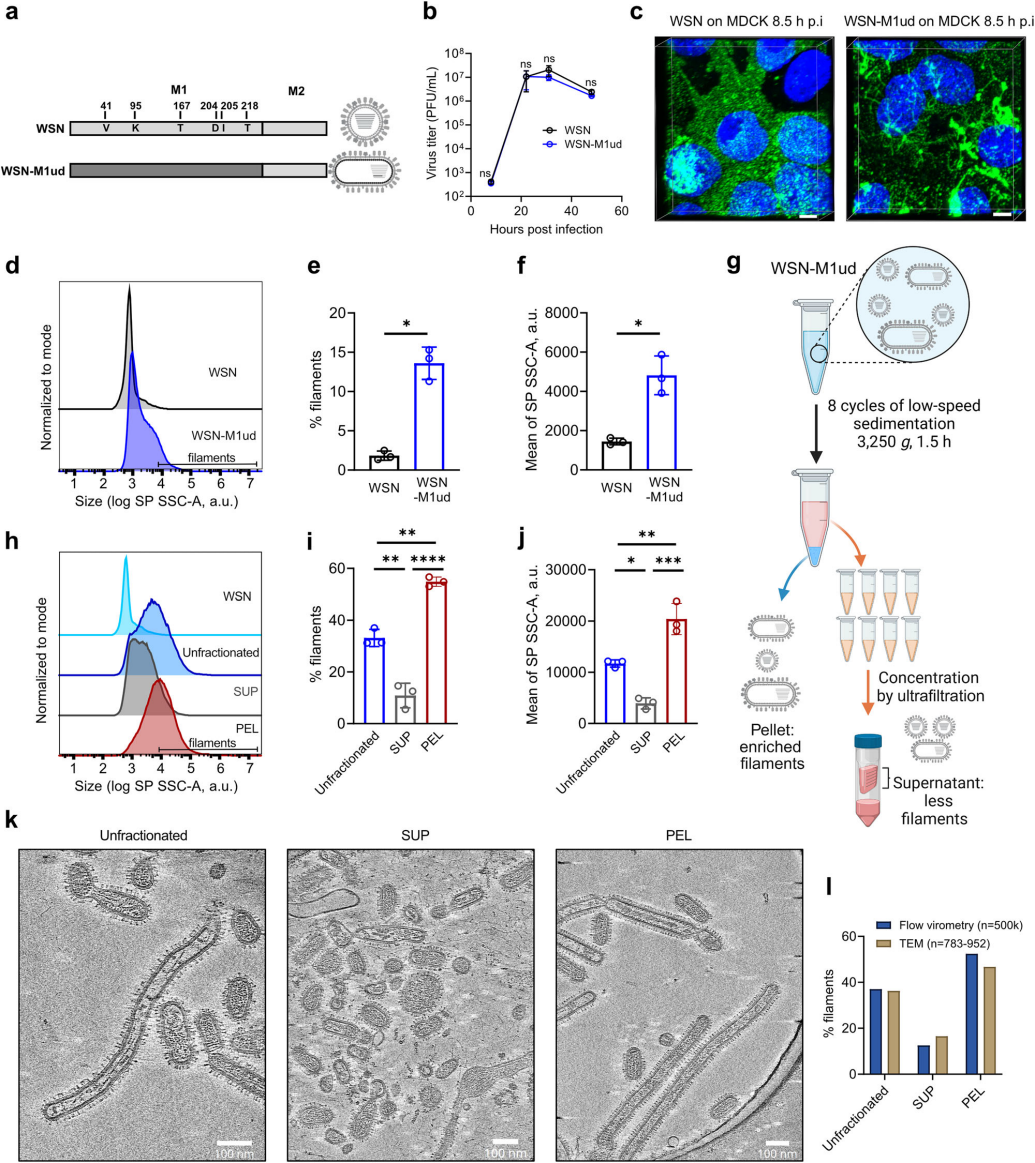
Characterization of spherical and filament-producing IAVs. **a** Schematic representation of spherical WSN and filament-producing WSN-M1ud IAVs. Light gray areas indicate sequences derived from WSN M1; dark areas represent Udorn M1 sequences. ud: Udorn. Amino acid differences between the M1 genes are indicated. Created in BioRender. Stertz, S. (https://BioRender.com/l32yd6f). **b** Replication kinetics of indicated viruses in MDCK cells. Cells were infected with viruses at MOI 0.001. Viral titers of cell culture supernatants were determined by plaque assay. Data represent mean ±  standard deviation (s.d.) from three independent measurements. Virus titers at each time point were compared by two-way ANOVA followed by Tukey’s post-test in GraphPad Prism. *p*-values ≥ 0.05 were considered not significant (ns). **c** Effect of M1 amino acid polymorphisms on budding morphology. MDCK cells were infected with the indicated viruses at MOI 3. Cells were fixed at 8.5 h post infection and stained for cell surface HA (green). Nuclei were stained with DAPI (blue). 3D reconstructed z-stacks were acquired via confocal microscopy. The scale bar corresponds to 5 μm. **d** Shape measurements of three biologically independent batches of WSN and WSN-M1ud virions by flow virometry, using the small particle side scatter (SP SSC) and fluorescence channels. Shown are representative SP SSC histograms of gated Alexa Fluor 488–labeled virion populations. The threshold for filamentous virions is indicated. The percentage of filamentous virions in each preparation is shown in (**e**), and mean SP SSC-A values related to virion size are shown in (**f**). Statistical analysis was performed using an unpaired t-test (two-tailed) in GraphPad Prism. *p* values < 0.05 were considered significant (*p* < 0.05 (*)). **g** Schematic workflow for enrichment of viral filaments. Created in BioRender. Stertz, S. (https://BioRender.com/dx1jtgp). **h** Shape of WSN-M1ud before and after fractionation into PEL and SUP fractions from (**g**), plus a WSN control were analyzed by flow virometry. The percentage of filamentous virions within three independent virus preparations is shown (**i**), and mean SP SSC-A values are shown in (**j**). Statistical analysis was performed using one-way ANOVA followed by Tukey’s multiple comparisons test in GraphPad Prism. *p* values < 0.05 were considered significant (*p* < 0.05 (*), *p* < 0.005 (**), p < 0.0005 (***), *p* < 0.00005 (****)). **k** Representative slice through cryo-electron tomogram of WSN-M1ud fractions. Scale bar: 100 nm. **l** Comparison of shape quantification of the same batch of WSN-M1ud fractions, obtained either by flow virometry using the SP SSC threshold indicated in (**h**), or by quantification of virions larger than 230 nm (*n*  =  783-952) from Cryo-TEM overview maps. For all data panels, plotted values are mean and s.d. of three biological replicates. Source data and exact *p* values are provided in the Source Data file.

To investigate the effects of viral morphology on infectivity in aerosol particles and host epithelium, we generated a pair of morphologically distinct but genetically comparable IAVs using reverse genetics (Fig. 1a). Strain A/WSN/33 (H1N1) (WSN) was chosen in order to achieve high enough virus titers required for aerosol experiments. WSN was engineered to generate filamentous WSN-M1ud virions by swapping the M1 encoding sequence with that of the filament-producing A/Udorn/307/72 (H3N2) (Udorn) strain^38,40^. WSN and WSN-M1ud have a total of six amino acid differences in the M1 protein (Fig. 1a). Using a multicycle replication assay, we found that WSN-M1ud replicated with kinetics comparable to the parental WSN strain in MDCK cells (Fig. 1b). WSN-M1ud has been shown previously to generate a heterogeneous population of virions, comprising predominantly intermediate-length bacilliform particles (120–300 nm) and a minor fraction (<10%) of long filaments (>300 nm)^40,41^, similar to that observed in human IAV isolates^42^. In contrast, wildtype WSN produces predominantly spherical particles (> 80%), with the remainder consisting of shorter bacilliform virions. To confirm the morphology phenotypes of these virions, we performed fluorescence imaging of unpermeabilized MDCK cells infected with either virus for 8.5 h by surface staining of HA glycoproteins (Fig. 1c). Consistent with previous findings^41,43,44^, we observed a punctate staining for HA of wildtype WSN, which has been described as a typical phenotype for budding spherical particles. In contrast, WSN-M1ud mutant produced long HA-positive protrusions from the apical cell membrane that represent viral filaments. Next, we employed flow virometry^18^ to profile virion shape of our preparations concentrated by ultrafiltration. Alexa Fluor 488-labeled virions were detected and resolved from background by both fluorescent and small particle side scatter (SP SSC) channels (Fig. 1d and Supplementary Fig. 1a). The SP SSC profiles of the gated virions revealed distinct scattering characteristics between WSN and WSN-M1ud, with the latter showing stronger scatter signals (Fig. 1d). To assess whether the stronger scatter signal was due to differences in virion morphology or due to so-called virion swarming or concentration-dependent aggregation, a dilution series of WSN and WSN-M1ud was measured by flow virometry (Supplementary Fig. 1a). We observed that counts of gated virions linearly decreased with higher dilution, while median fluorescent intensity measurements remained constant (Supplementary Fig. 1b–c). This indicates that the viruses are monodisperse and that concentration-dependent viral aggregates are largely absent from the sample^45^. Quantification across three independent preparations confirmed that WSN-M1ud contained a higher proportion of filaments and, overall, a population of larger virions (Fig. 1e, f).

Given that the proportion of filamentous virions, defined as virions with scatter signals greater than WSN virions, was below 20% in our preparations, we modified the purification protocol and enriched the filamentous virions in WSN-M1ud using low-speed sedimentation^37^ (Fig. 1g). The resulting fractions were analyzed by flow virometry (Fig. 1h–j) and cryo-electron microscopy (cryo-EM) (Fig. 1k). Importantly, also here we found that virion aggregation was largely absent (Supplementary Fig. 2). The unfractionated WSN-M1ud already contained 30% of elongated virions, presumably due to the changes in the protocol for virus purification. Incorporating the enrichment step based on low-speed sedimentation led to a further increase in filament content. The pellet (PEL) fraction contained about 55% filamentous virions compared to 30% for unfractionated WSN-M1ud, while the supernatant (SUP) mainly consisted of spherical and bacilliform virions (Fig. 1h–k). Notably, the relative proportion of filaments estimated by flow virometry closely matched values obtained from cryo-EM quantification (Fig. 1l, and Supplementary Fig. 3). Given that the purified fractions are genetically identical and differ only in their proportion of filamentous versus spherical virions, we selected this system for further analysis.

### Differential inactivation kinetics of spherical and filamentous IAV mediated by temperature, pH, and high salinity in bulk

**Fig. 2:**
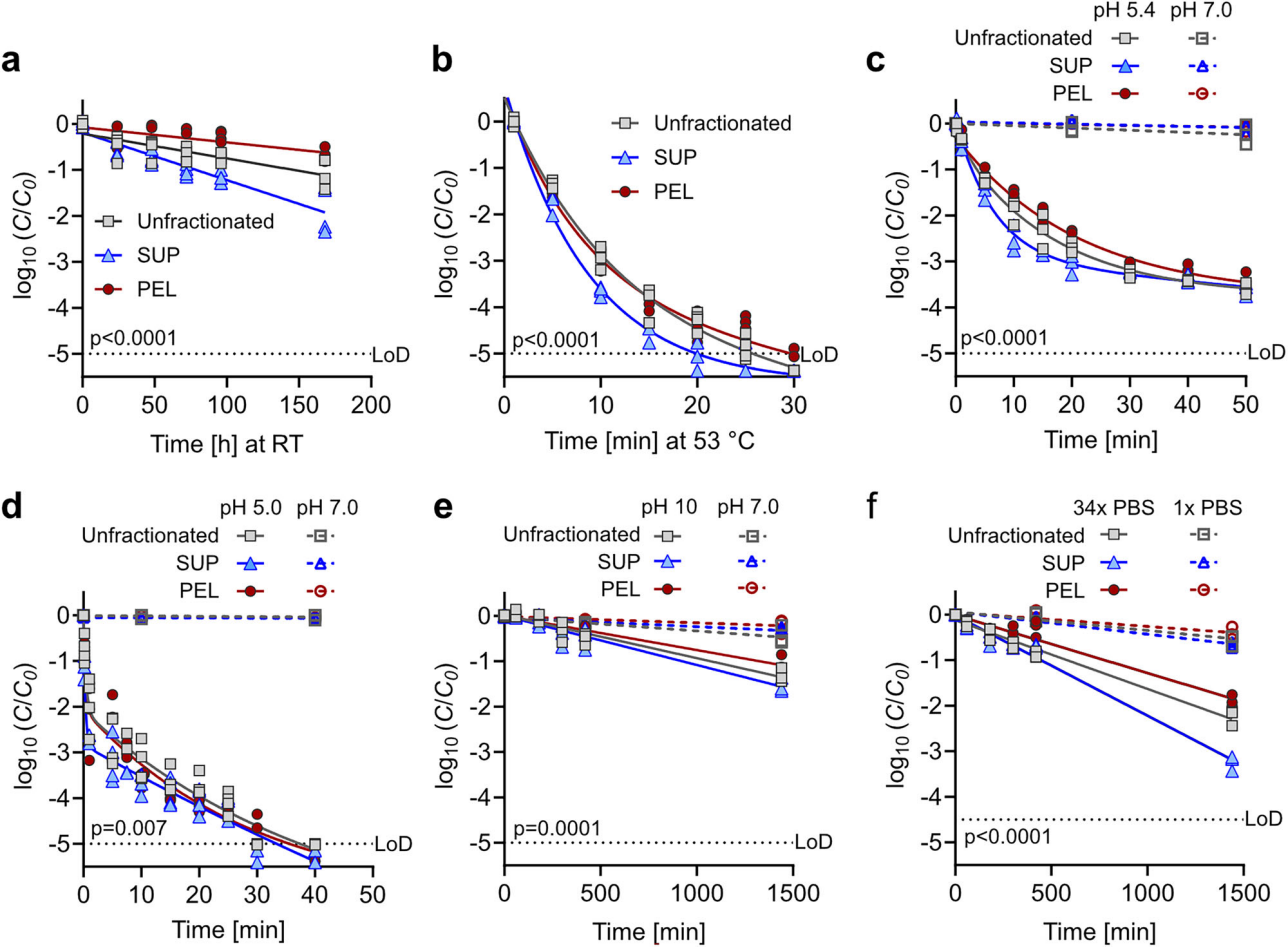
Infectivity decay for WSN-M1ud unfractionated, SUP, and filament-enriched PEL fractions in bulk solutions under varying temperature, pH, and high-salinity conditions over time. **a**, **b** log_10_ reduction in infectious virus titers of WSN-M1ud fractions in PBS. Infectivity was assessed by plaque assay following incubation at RT (24 ± 1 °C) (**a**) or at 53 °C (**b**) for the indicated time points and normalized to viral inocula. Plotted lines represent the mean of three independent experiments and are fitted using simple linear regression. Statistical significance between the slopes of the regression lines for the SUP and PEL samples was assessed using analysis of covariance (ANCOVA) in GraphPad Prism. **c**, **d** pH dependence: log_10_ reduction in infectious virus titers following exposure to aqueous citric acid/Na_2_HPO_4_ buffer adjusted to pH 5.4 (**c**) and 5.0 (**d**), or normalized to the pH 7.0, 10 s sample. Each dot represents a measurement from an independent experiment. Data shown are from three independent experiments and were fitted using nonlinear regression. The statistical significance between the fitted curves for the SUP and PEL samples was determined by the one-sided extra sum-of-squares F test in GraphPad Prism. **e** Same as (**c**, **d**), except the viruses were exposed to aqueous Na_2_CO_3_/NaHCO_3_ buffer adjusted to pH 10.0. Plotted lines represent the mean of three independent experiments and are fitted using simple linear regression. Statistical significance between the slopes of the regression lines for SUP and PEL fractions was assessed using ANCOVA in GraphPad. **f** Salt concentration dependence: log_10_ reduction in infectious virus titers of WSN-M1ud viral fractions in 1× PBS and 34× PBS, at the same initial virus titer normalized to their respective controls (after 10 s). Plotted lines represent the mean of three independent experiments and are fitted using simple linear regression. Statistical significance between the slopes of the regression lines for the SUP and PEL samples was assessed using ANCOVA in GraphPad Prism. **a–f** *P* values are shown in the bottom left corner; a *p* value < 0.05 was considered statistically significant. The dotted horizontal line represents the assay’s limit of detection (LoD). Source data and exact *p* values are provided in the Source Data file.

Airborne transmission of IAV has been shown to be influenced by temperature and humidity in animal models^46^. To evaluate the effect of temperature on the stability of IAV virions differing in shape, we used the purified fractions of WSN-M1ud (SUP and PEL) and compared their kinetics of infectivity loss in phosphate-buffered saline (PBS) under environmentally relevant room temperature (RT) conditions (Fig. 2a). The unfractionated WSN-M1ud was included for comparison. The PEL fraction enriched in filamentous virions displayed significantly higher stability than the SUP fraction (Fig. 2a). We next examined the thermal stability of these virions by incubating them at 53 °C for up to 30 min and found that the PEL fraction showed the slowest decay (Fig. 2b). These findings suggest that filamentous morphology protects from temperature-mediated inactivation.

In the next step, we assessed pH-mediated inactivation of IAV differing in shape.^29^. We had previously shown that freshly exhaled aerosol particles with diameters smaller than a few micrometers can reach a terminal acidic pH value of about 4 within a few minutes through rapid uptake of gaseous acids contained in typical room air. At this pH, acid-labile viruses such as IAV become rapidly (<2 minutes) inactivated in various matrices^28,29,47^. Alkaline pH (~10) has also been suggested to occur shortly after aerosol particle exhalation due to CO_2_ evaporation^28,31–34^, and may affect SARS-CoV-2 infectivity^31,33^. Building on these insights, we aimed to reproduce the differing pH conditions in respiratory aerosol particles and investigate in the corresponding bulk solutions how viral morphology influences pH stability of IAV. To this end, we compared the inactivation dynamics of the two purified fractions (SUP and PEL) and the unfractionated WSN-M1ud in bulk solutions adjusted to acidic pH values of 5.4 and 5.0 (Fig. 2c, d) and alkaline pH 10.0 (Fig. 2e). IAVs added into a solution with pH 7.0 and placed at RT showed no decay for the entire monitoring period. The SUP fraction, containing the fewest filamentous virions, displayed a faster decay in infectivity than unfractionated and PEL fractions at pH 5.4 over 30 min and pH 5.0 during the first 10 min of exposure (Fig. 2c, d). This pattern suggests that filamentous IAV particles are more stable under acidic conditions. Consistent with previous findings^27,48^, the infectivity loss of WSN-M1ud viral fractions was considerably smaller in pH 10.0 solution compared to pH 5.0 or 5.4, but again, the PEL fraction displayed increased stability over the SUP fraction (Fig. 2e).

Besides pH, high salt concentrations caused by water loss due to evaporation represent another physicochemical condition that aerosolized IAV encounters, contributing to viral inactivation^27^. At RH levels below 75%^28^, solutes surpass solubility limits for NaCl in equilibrated aerosols, leading to the formation of supersaturated salt concentrations, which are experimentally inaccessible in the bulk phase system. At high RH above the efflorescence relative humidity (ERH) of the salts, aerosols remain liquid. The water activity (*ɑ*_*w*_*)* of the complex solute mixture within the aerosol equilibrates within seconds with the ambient RH. For RH > ERH, the resulting steady-state salt concentrations within an equilibrated aerosol can be approximated using bulk solutions with solute molarities adjusted to the target RH. To investigate whether viral morphology influences IAV sensitivity to high salinity, we reconstituted the water activity of aqueous solution in equilibrated aerosols at 80% RH using a 34× PBS solution^49^ that has *ɑ*_*w*_ = 0.80 (Fig. 2f). The PEL fraction, enriched in filamentous virions, maintained infectivity most effectively in 34× PBS, showing <2log_10_ decay after 24 h. In contrast, the SUP fraction, which contained the fewest filaments, decayed most rapidly. The unfractionated WSN-M1ud displayed intermediate stability. Together, these results indicate that filamentous morphology correlates with reduced sensitivity to high salt concentrations.

In order to test if a smaller proportion of filamentous virions would already lead to increased stability, we next tested the pair of viruses differing in their M1 gene (Fig. 1a) for infectivity decay at RT, at pH 5.0, and at high salt concentrations (Supplementary Fig. 4a–c). When monitoring decay at RT over 7 days, no difference could be detected between WSN and WSN-M1ud (Supplementary Fig. 4a). Incubation at pH 5.0 for different times revealed increased stability of WSN compared to WSN-M1ud (Supplementary Fig. 4b), whereas at high salt concentrations WSN-M1ud displayed higher stability (Supplementary Fig. 4c). These data suggest that stability at high salt concentration correlates with the proportion of filamentous virions when comparing WSN and WSN-M1ud or shape-enriched fractions of WSN-M1ud, and both systems can be used to study salt-dependent virus inactivation. However, opposite trends were observed for acid stability in the two viral systems, leading us to speculate that the amino acid differences in M1 between WSN and WSN-M1ud not only affect viral morphology but also other viral traits that impact acid stability. The shape-enriched fractions of WSN-M1ud thus represent a better model to study shape-related effects for low pH-dependent virus inactivation.

### Filamentous IAV displays enhanced stability at high RH and in acidified air

**Fig. 3:**
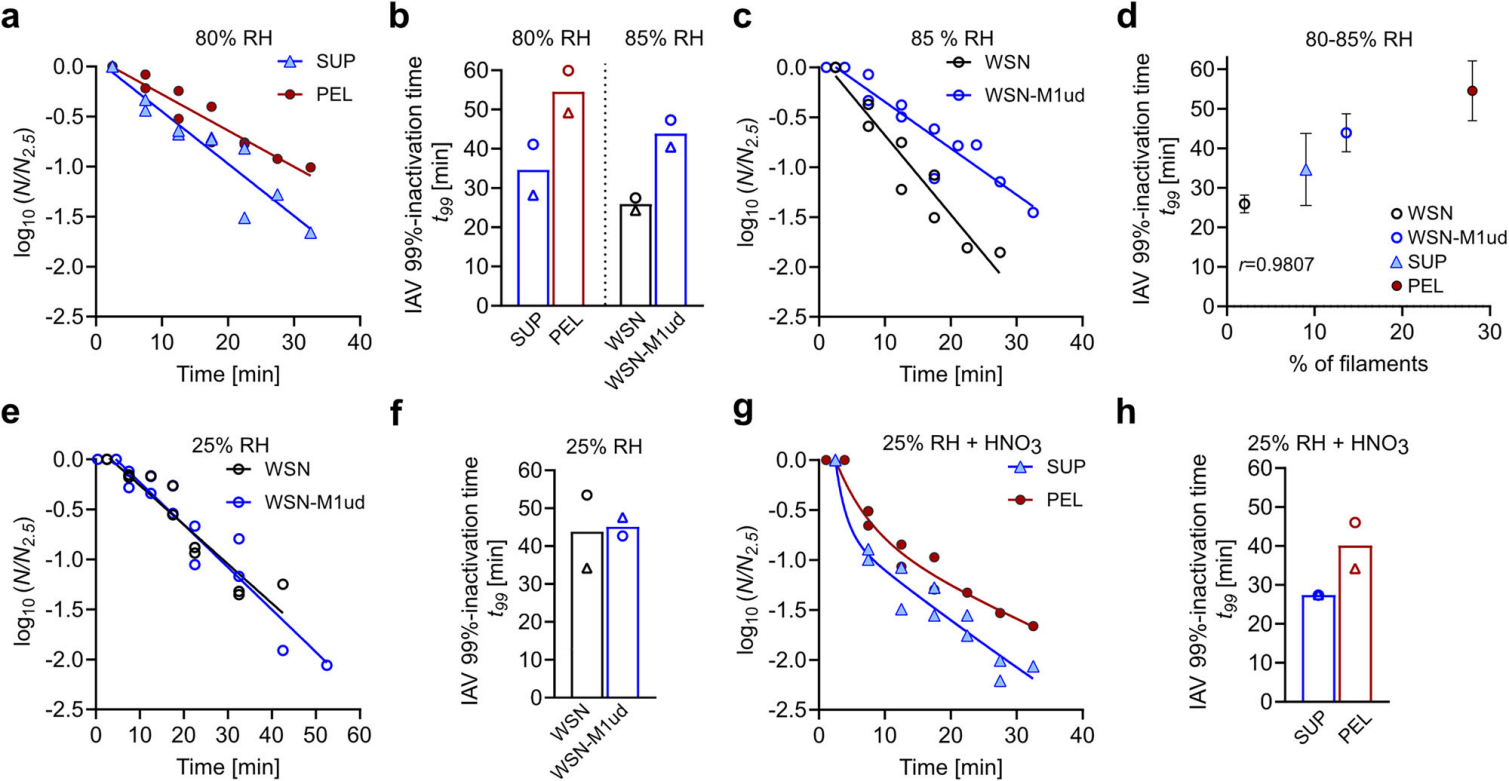
Inactivation of differently shaped IAV in aerosol particles. **a** WSN-M1ud SUP or PEL suspended in PBS were aerosolized and introduced into an aerosol chamber maintained at 80% RH at RT (24 °C ± 1 °C). The chamber was supplied with purified air to assess the salt-mediated inactivation. Samples were collected every 5 min (4 min sampling and 1 min for Petri dish exchange) using the BioSpot-VIVAS for 35 min to measure virus titers (PFU/mL) and total viral particles (GC/mL) using plaque assay and dPCR, respectively. The ratio of infectious to total virus particles at time *t* is denoted as *N*(*t*)/*N*_2.5_, where *N*_2.5_ represents this ratio at first sampling time (0.5–4.5 min, after aerosolization). Data are fitted using simple linear regression. **b** Time required for a 99% reduction (*t*_99_) in infectious virus titer corresponding to datasets in (**a**) and (**c**), respectively. (**c**) Same as (**a**), except WSN and WSN-M1ud were used, and experiments were performed at 85% RH. **d** *t*_99_ values from (**b**) plotted against virion shapes determined by flow virometry for the samples in (**a**) and (**c**). *r* value derived from two-tailed Pearson’s correlation analysis performed in GraphPad Prism. **e** Same as (**c**), except the chamber humidity was set to 25% RH. Corresponding *t*_99_ values are shown in (**f**). **g** Same as (**a**), except the viruses were suspended in SLF, experiments were performed at 25% RH and the chamber air was additionally supplemented with HNO_3_ to assess pH-mediated inactivation. Corresponding *t*_99_ values are shown in (**h**). For each data panel, points represent two independent experiments (*n* = 2). For data (**c**, **f**, and **h**) each independent dataset is shown using the same symbol shape (either empty circles or triangles). Source data are provided in the Source Data file.

To test if our findings from the bulk experiments extend to aerosol particles, we nebulized viruses using the LAPI BREATH, an aerosol chamber system that allows to retrieve inactivation rates of pathogens in aerosols^50^. While aerosol particles with a diameter of up to 7 μm in dry size are produced, the vast majority are in the submicrometer range (Supplementary Fig. 5a). We assume the infectious viruses to be volume-proportionally distributed in the particles. Under this assumption, about 98.5% of all viruses are in submicron particles (Supplementary Fig. 5b). We initially assessed the stability and infectivity of aerosolized SUP and PEL fractions of WSN-M1ud, which were exposed to 80% RH at RT and collected at designated time points. Notably, filtered air and PBS as the aerosol matrix were used in this system. This setup was specifically designed to evaluate the impact of viral morphology on IAV sensitivity to high salinity in deliquesced saline aerosol particles while minimizing changes in pH. To determine the decay of IAV infectivity, infectious virus (plaque forming units, PFU/mL) and total virus concentration (genomic copies, GC/mL) were determined for all time points. The filament-enriched WSN-M1ud PEL fraction displayed slower inactivation kinetics compared to the SUP fraction (Fig. 3a), with a longer time required to reach 99% inactivation of the initial aerosolized virus population (*t*_99_) observed across two independent experiments (Fig. 3b, left). A similar higher stability of filament-producing WSN-M1ud over WSN (Fig. 3c) along with a higher *t*_99_ (Fig. 3b, right) occurred at similar experimental conditions at 85% RH. Importantly, we observed a strong correlation between filament prevalence and *t*_99_ (Fig. 3d). This enhanced stability for filamentous virions aligns well with the findings from the high salt bulk experiments (Fig. 2f and Supplementary Fig. 4c).

When repeating the chamber experiment with WSN and WSN-M1ud in the same matrix but at 25% RH, we observed similar first-order inactivation kinetics for both viruses, corresponding to a non-significant difference in the *t*_99_ for these viruses (Fig. 3e, f)^50^. Similar viral decay is likely attributed to rapid efflorescence occurring in aerosols at 25% RH, which is below ERH^50^, leading to substantial depletion of the liquid phase. The resulting almost complete suppression of the physicochemical reactions of IAV in the aqueous phase at low RH could possibly explain the similar inactivation kinetics of the different virus types. For this set of experiments, we measured dry aerosol particle size distribution using a scanning mobility particle sizer (SMPS) for two independent chamber experiments. We found that the total aerosol concentration of WSN and WSN-M1ud, measured as integrated size distributions over periods of 4 minutes, varied across experiments (Supplementary Fig. 5c), but correlated with the genomic copy (GC) concentrations recovered from aerosol samples. Despite this variability, the decay kinetics from the two experiments were highly similar (Fig. 3e). These findings support the use of GC-normalized infectivity measurements for assessing IAV inactivation kinetics in our aerosol chamber.

Next, we experimentally determined the inactivation kinetics of the shape-enriched fractions of WSN-M1ud in aerosol particles with acidity as the main driver of inactivation. We used synthetic lung fluid (SLF) as matrix instead of PBS and introduced HNO_3_ into the chamber air at 25% RH. Based on our previously established modeling approach using ResAM we expect that particles with an initial radius of 1 μm (corresponding to a radius of 300 nm in dry size) or less acidify to IAV-inactivating pH values in less than 100 s (Supplementary Fig. 5d). In particles with an initial radius of 5 μm (corresponding to a dry radius of 1.5 μm) acidification would take ~16 min, whereas for particles with an initial radius of > 10 μm it would take 1 h or longer (Supplementary Fig. 5d). Since an estimated 98.5% of the viruses in our aerosol chamber will be present in particles with an initial radius of 1 μm or less (Supplementary Fig. 5b) we expect to observe a substantial decrease in infectivity in our experimental setting. In line with our model results and consistent with the results observed in bulk solutions at acidic pH, a slower decay and thus a higher *t*_99_ of filamentous virions was observed in aerosol particles exposed to air enriched with 10 ppbv HNO_3_ (Fig. 3g–h). These findings suggest that filamentous IAV also exhibits reduced sensitivity to low pH-mediated inactivation in aerosol particles compared to spherical virions.

Aerosol particle size is another critical factor that influences IAV aerostability^51,52^. Given the size difference between filamentous and spherical virions, variations in the size distribution of aerosols incorporating these viruses could be expected. Based on this, we hypothesized that filamentous virions would be associated with larger aerosol particles due to their elongated shape, potentially contributing to increased stability because of prolonged *t*_99_ at RH > 80%. To test this hypothesis, aerosolized WSN and WSN-M1ud were collected via an Andersen Impactor that separates aerosol particles into six stages with specific size ranges. Each fraction was subsequently tested for concentrations of infectious virus and GC. Our data revealed distinct patterns in the size-resolved distribution of infectious virus for the two morphological variants (Supplementary Fig. 5e). We observed that viral genome content and infectivity declined from stage 6 to stage 1 for WSN-M1ud viruses, whereas genome content and infectivity peaked in stage 4 for WSN virions, a result contrary to our initial expectation. However, it should be noted that we were not able to quantify virus shape in the fractions from the Andersen impactor as the minimum input for flow virometry could not be reached.

Overall, our results indicate morphology-dependent stability of IAV under the physicochemical conditions encountered in expiratory aerosol particles, with filamentous virions displaying enhanced stability under high salt and low pH conditions. Moreover, the distinct inactivation patterns observed between filamentous and spherical virions in the bulk system are predictive of their behavior in the aerosol system.

### IAV shape modulates sensitivity to HA-binding neutralizing IgA and IgG antibodies

**Fig. 4:**
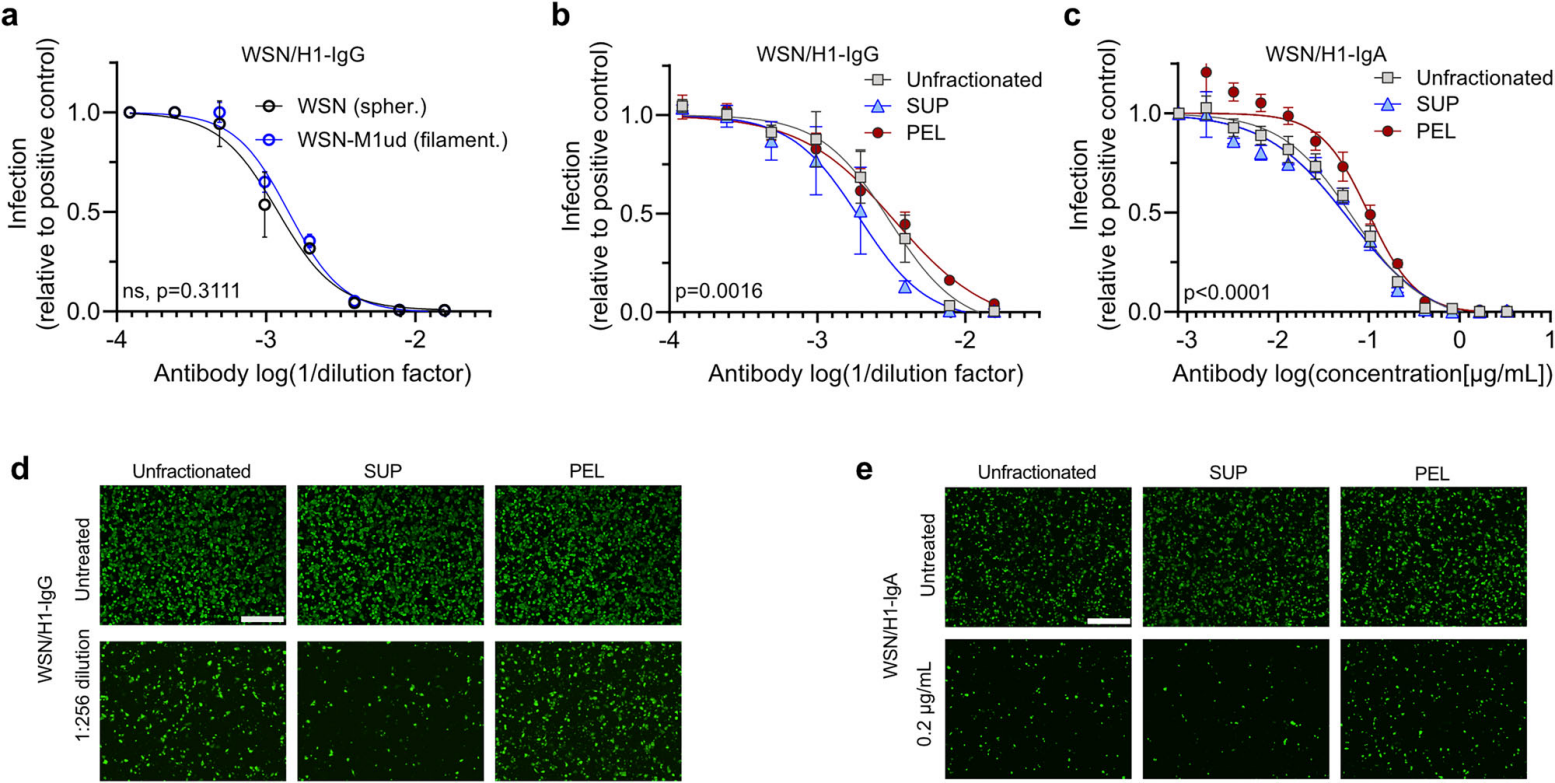
Filament-producing IAV is more resistant to neutralization by antibodies. **a**–**c** Microneutralization assay of indicated IAV was performed by pre-incubating IAV with serial dilutions of HA-binding IgG (**a**, **b**) or IgA (**c**) antibody for 1 h at RT. A549 cells were infected with the virus-antibody mixtures. At 7 h post-infection, cells were fixed and stained for NP. Mean fluorescence signal intensity (MFI) of cells was analyzed by microscopy in an IncuCyteS3 (Sartorius). Representative images corresponding to panels (**b**) and (**c**) are shown in panels (**d**) and (**e**), respectively. The scale bar represents 400 μm. Non-linear regression was used for fitting curves. Error bars are mean ± S.E.M of three independent replicates. Statistical significance between fitted curves for SUP and PEL fraction in (**a**–**c**) was assessed using the one-sided extra sum-of-squares F test in GraphPad Prism. *p* < 0.05 was considered significant. Source data and exact *p* values are provided in the Source Data file.

When IAV retains infectivity during airborne transmission and enters the respiratory tract of a new host, the virus will encounter a range of barriers to infection, including neutralizing antibodies and respiratory mucus. We aimed to evaluate if viral shape contributes to IAV infectivity under these conditions. To determine the optimal inoculum dose for single-cycle infection in A549 cells, we assessed the relationship between input virus dose and the mean fluorescence intensity (MFI) of infected cells (Supplementary Fig. 6a). Infection increased linearly across an MOI range of 0.625 to 2 for all three viral fractions (Supplementary Fig. 6b). We thus selected an MOI of 1.25 as the optimal input dose, providing a moderate, quantifiable MFI without reaching saturation. Initial tests with the virus pair WSN and WSN-M1ud showed only minor differences in sensitivity to an HA-binding neutralizing antibody (IgG) (Fig. 4a), despite previous reports suggesting filamentous particles have an advantage^37,38^. This discrepancy could be due to a lower proportion of filamentous virions in our preparations than prior studies^38^ (Fig. 1e). Notably, when testing the shape-enriched fractions of WSN-M1ud we observed differences in the fractions’ sensitivity to inhibition by the HA-specific (IgG) antibody, with filaments (PEL fraction) demonstrating reduced sensitivity compared to the SUP fraction (Fig. 4b, d). Importantly, in the absence of antibody, the three viral fractions displayed similar infectivity in A549 cells (Fig. 4d). While IgG is the predominant isotype induced systemically, we also tested whether the reduced sensitivity of the filament-enriched fraction extends to IgA, the predominant isotype in mucosal tissues, such as the upper respiratory tract. Indeed, our results revealed a higher resistance of filament-enriched PEL fraction to an HA-specific IgA antibody compared to the SUP fraction (Fig. 4c, e). Of note, the unfractionated virus preparation showed comparable IgG sensitivity to the PEL fraction but was closer to the SUP fraction in the IgA experiment. In summary, we could detect reduced sensitivity to antibody-mediated neutralization for filamentous IAV compared to the spherical counterpart, which is in line with previous reports.

### Filamentous IAV possesses an infectivity advantage in human airway epithelial cultures that secrete mucus

**Fig. 5:**
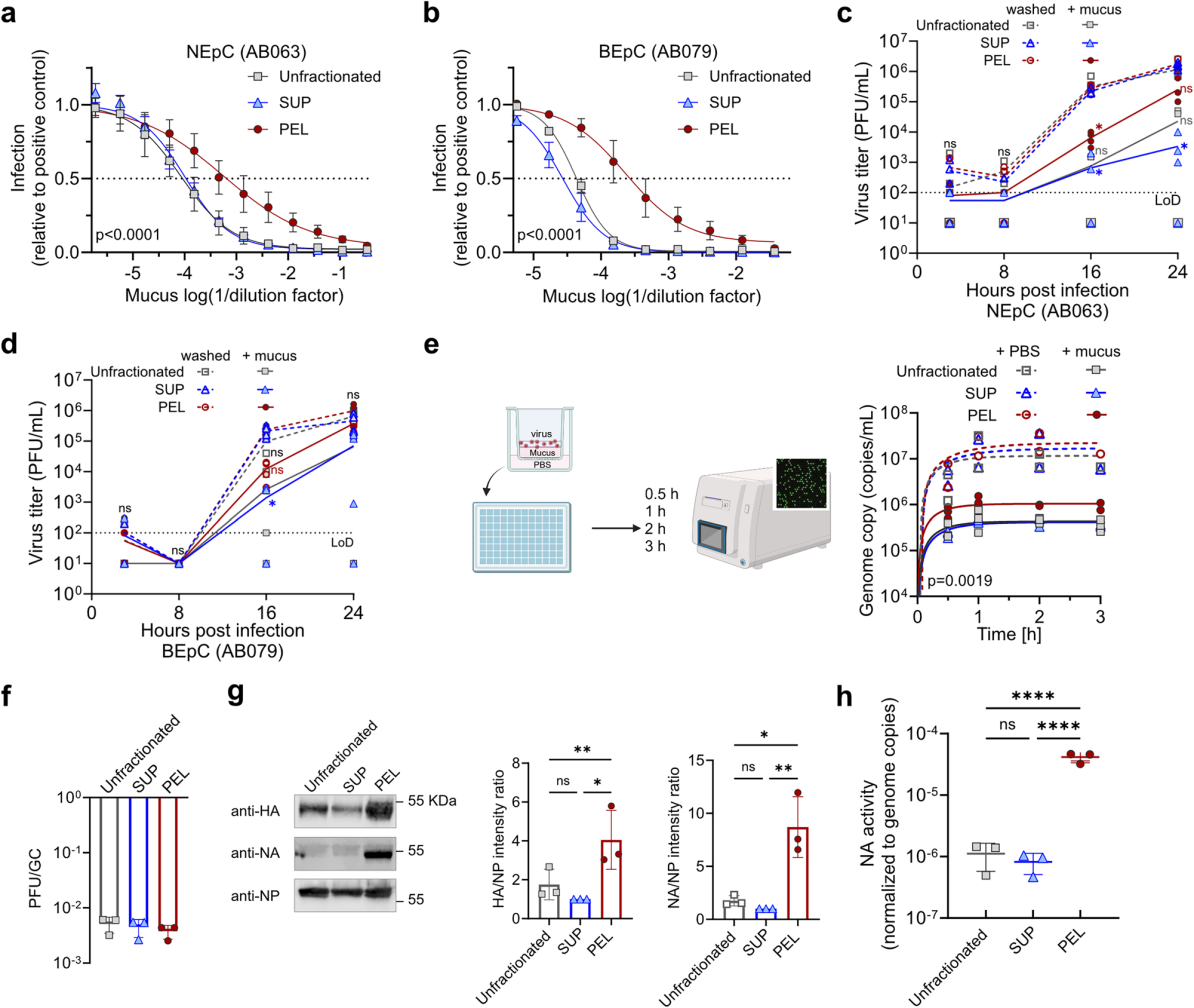
Filament-producing IAV is more resistant to neutralization by mucus. **a**,** b** Neutralization of the indicated WSN-M1ud virus fractions by mucus harvested from human NEpC (**a**) or BEpC (**b**). Viruses were pre-incubated with serial dilutions of mucus on ice for 1 h. A549 cells were infected with the virus-mucus mixtures, fixed at 7 h post-infection, and stained for viral nucleoprotein (NP). Mean fluorescence intensity (MFI) was measured and normalized to values from virus-infected cells without mucus treatment. Plotted are mean and S.E.M from three independent experiments, with non-linear regression curves fitted to the data. Statistical significance between fitted curves was assessed using the one-sided Extra sum-of-squares F test in GraphPad Prism. *p* < 0.05 was considered significant. **c**,** d** Replication kinetics of WSN-M1ud fractions on NEpC (**c**) or BEpC (**d**) culture**d** at ALI. Prior to infection, mucus was either removed by PBS washing (“washed”) or left intact (“+ mucus”). The dotted line indicates the assay’s limit of detection (LoD) at 100 PFU/mL. Statistical analysis was performed using two-way ANOVA followed by Tukey’s post-test in GraphPad Prism. *p* values ≥ 0.05 were considered not significant (ns), and *p* values < 0.05 were considered significant (*p* < 0.05 (*), *p* < 0.005 (**)). When all pairwise comparisons within a single time point yielded non-significant results, “ns” is displayed in black. (**e**) Schematic representation illustrates the diffusion of viruses through a 30μL-thick mucus layer (solid line) or PBS control (dotted line) overlaying a transwell insert. Created in BioRender. Stertz, S. (2026): https://BioRender.com/s6owsej. At 30, 60, 120, and 180 min 50 μL samples were collected from the basal side to quantify viral genome copies by dPCR. Shown are three independent experiments, with non-linear regression curves fitted to the data. Statistical significance between fitted curves was assessed using the one-sided Extra sum-of-squares F test in GraphPad Prism. **f** Comparison of the ratio of infectious viral titer (PFU/mL) to total virus particles (GC/mL) in stocks of indicated viruses. Plotted are mean and s.d. from three independent experiments. **g** Western blots of virus stock lysates containing equal amounts of input virus particles were performed for unfractionated pleomorphic WSN-M1ud, SUP, and PEL fractions. Blots were probed for HA, NA, and nucleoprotein (NP). Quantitative analysis of HA and NA levels relative to NP is shown in the middle and right panels, based on three independent virus preparations. Plotted are mean and S.D. **h** The NA activity of each WSN-M1ud viral fraction (with equal GC number) was measured with the NA-Star Influenza Neuraminidase Inhibitor Resistance Detection Kit. Data are mean ± s.d. from three independent experiments. Statistical analysis was performed using one-way ANOVA followed by Tukey’s post-test (**g,** **h**). *p* values < 0.05 were considered significant (*p* < 0.05 (*), *p* < 0.005 (**), *p* < 0.0001 (****)). Source data and exact *p* values are provided in the Source Data file.

Another barrier that inhibits IAV infectivity in the human respiratory tract is the mucus lining of the airway epithelium. While viral NA activity correlates with mucus sensitivity^53^, the association between viral shape and its ability to penetrate the mucus layer, along with the underlying mechanisms, remain poorly understood. We performed a microneutralization assay to assess how infection with filamentous and spherical virions is inhibited by mucus produced from differentiated primary human airway epithelial cultures of nasal (NEpC) and bronchial (BEpC) origin. We found that unfractionated and spherical SUP viral fractions exhibited significantly higher susceptibility to mucus neutralization than the PEL fraction (Fig. 5a–b). Next, we evaluated the inhibitory effect of mucus on the growth kinetics of IAV of different shapes in NEpCs and BEpCs. After mucus removal through extensive apical PBS washes, the three viral fractions replicated with similar kinetics and grew to titers of 10^6^ PFU/mL in both NEpCs and BEpCs (Fig. 5c–d). In the presence of mucus, however, the growth of unfractionated and spherical SUP fraction (gray and blue solid lines) was strongly reduced or completely abrogated in NEpCs and BEpCs. In contrast, the filament-enriched PEL fraction (red solid lines) was less prone to mucus neutralization and still replicated to titers of about 10^5^ PFU/mL.

To demonstrate that this growth advantage on the airway cultures in the presence of mucus was indeed due to better mucus penetration of the filamentous IAV we developed a specialized mucus diffusion assay using transwell inserts, based on a previously published setup^54^. In the apical compartment, we placed 30 µl of mucus to form a layer. A single drop (3 µl) of virus was then overlaid on the mucus layer, allowing us to assess IAV particle diffusion through this defined mucus layer over designated time points (Fig. 5e). The diffusion was quantified by measuring the amount of virus that reached 400 µl of PBS in the basal compartment using dPCR. While all virion populations, regardless of size, exhibited similar diffusion rates in PBS controls, the filament-enriched PEL fraction (red solid line) moved more rapidly through mucus than the other two viral fractions (gray and blue solid line), with a higher number of virions reaching the basal side (Fig. 5e).

Mucus can inhibit IAV through binding of virions to sialic acid, which is abundantly present on mucins, while viral NA can counteract this binding by cleaving off sialic acid moieties^55^. Therefore, a functional NA/HA balance is crucial for IAV mobility through mucus. Our Western blotting revealed ~four times more HA in the filament-enriched PEL fraction over spherical SUP fraction when normalizing to viral genome copies (Fig. 5f, g). Furthermore, the filament-enriched PEL fraction exhibited an 8-fold increase in NA protein level (Fig. 5g) and 50-fold increase in NA activity (Fig. 5h). The increase in HA content is in line with previous reports and can explain the lower sensitivity of the filamentous virions to antibody-mediated neutralization^38^. Given the higher increase in NA compared to HA these findings also support the concept that filamentous IAV with a higher NA:HA ratio may be more resistant to mucus inhibition.

Collectively, these findings reveal that filamentous morphology correlates with higher infectivity in the presence of HA-targeting IgA and IgG neutralizing antibodies, as well as under conditions of respiratory mucus-mediated inhibition, thereby facilitating viral infection and replication in human airway cultures.

## Discussion

IAV strains that cause epidemics and pandemics in humans are efficiently transmitted via IRPs, enabling rapid spread. Understanding the mechanisms governing airborne transmissibility is critical for pandemic preparedness and the development of effective mitigation strategies. Viruses must navigate multiple layers of hostile environments presented by the IRP microenvironment and the host, as they retain infectivity from one host to the next. Many animal transmission models have revealed the viral genetic traits^56,57^ and environmental factors (temperature and RH) that influence transmissibility^46,58^. For example, filamentous morphology has been correlated with airborne transmissibility^22^. However, this has been tested using animal models that emphasize the biological consequences of transmission events but do not clarify whether, and how, virion pleomorphism contributes to airborne stability. In particular, the relationship between viral shape and IAV aerostability in response to diverse aerosol microenvironments has not been probed yet. Using an aerosol chamber, we experimentally characterized the stability of spherical and filamentous IAV in aerosol particles under conditions of acidic pH or high salt concentrations coupled with lowered water activity, while concurrently testing them in bulk systems. Our results reveal that filamentous shape confers enhanced stability to IAV under high salt and low pH conditions.

We experimentally examined the stability of morphologically distinct virions in both, effloresced and deliquesced PBS aerosols. Given that PBS is a pure salt solution that buffers pH fluctuations, we aimed to assess the impact of viral morphology on IAV inactivation driven solely by varying salinities in aerosol particles. Our results reveal similar viral decay kinetics for spherical and filamentous virions in effloresced PBS aerosol particles at 25% RH (Fig. 3e), whereas filamentous virions show enhanced stability in aqueous PBS–aerosol particles at 80-85% RH (Fig. 3a–c). At low RH, aqueous-phase reactions are likely largely halted due to salt crystallization, resulting in similarly restricted or absent virus–solution interactions. Consequently, viruses exhibit comparable inactivation rates regardless of morphology under these conditions. In contrast, under equilibrium conditions at 80-85% RH, solute molarities increase ~26–34-fold upon nebulization as water partitions into the gas phase. A recent study showed that high salinity conditions can inactivate IAV by disrupting its structure^27^. Based on this, we hypothesize that the enhanced stability of filamentous virions under these conditions, both in aerosol particles and bulk systems, may be attributed to their intrinsically higher structural resilience under high salt concentrations. Further experiments are required to reveal how filaments maintain their structural integrity. We speculate that one possibility could be that filamentous virions are only partially engulfed by the liquid phase of drying aerosol particles, thereby reducing the surface area exposed to high salinity and conferring a protective advantage.

Aerosol pH is influenced by air composition, particularly by the partitioning of volatile acidic and alkaline compounds between the gas phase and aerosol phase. According to our model results, the pH value of exhaled aerosol particles with a diameter of a few micrometers or less decreases to about 4 within a few minutes due to the uptake of condensable acidic substances, such as HNO_3_, in typical indoor air^28,34^. We assessed the role of pH in the stability of aerosolized spherical and filament-enriched virions using SLF as matrix and supplementation of chamber air with HNO_3_ at 25% RH. At this RH, the presence of organics in SLF affects the phase state of an aerosol particle by increasing the viscosity and surface tension that influences efflorescence^50^. Based on electrodynamic balance and optical microscopy measurements of SLF particles from Luo et al.^28^, we hypothesize that a residual liquid phase can coexist with the salt crystal in an efflorescing SLF–aerosol particle. Such a liquid microenvironment could allow for pH-dependent interactions with virions even at low RH. However, these observations were made on micrometer-sized SLF particles whose morphology evolved over time; whether the same phase behavior occurs in submicrometer dry aerosol particles remains to be confirmed. Although 80% RH would maintain the aerosol particles in an aqueous state, this condition was not experimentally tractable in our setup because viral infectivity is highly sensitive to nitric acid in the air at high RH. Since IAVs are highly sensitive to low pH^28^ but remain stable for hours when exposed to alkaline conditions, it is likely that the inactivating effect of acidic pH in submicrometer dry aerosol particles would outweigh that of high salt concentrations during water evaporation. Here, we report that filamentous IAV exhibits greater stability in acidic pH solutions, as well as in acidified aerosol particles. It is known that the loss of infectivity of IAV under acidic pH conditions is caused by an irreversible conformational rearrangement of the surface glycoprotein HA, wherein it transitions from a pre-fusion to a post-fusion state^59^. This conformational shift is well-documented to abolish the virus’ ability to bind to host cell receptors, resulting in viral inactivation^59–62^. As filamentous virions have significantly more HA trimers present in their envelope, their chance of retaining enough intact trimers for infection is higher. For larger particles, the pH may remain alkaline for hours to days^34^. Under such conditions, our pH 10 bulk data suggest that filamentous virions may also retain an infectivity advantage. However, this remains to be confirmed in an IRP system.

Besides our results on aerosol stability, we also established functional relationships between viral morphology and IAV infectivity under attenuating pressures exerted by mucosal immunity, including neutralizing antibodies and human respiratory mucus, particularly in an ex vivo model of the human airway epithelium. Previous studies have shown that filamentous IAV particles carry more surface glycoproteins, potentially aiding in immune evasion or enhancing attachment under conditions of HA inactivation^37,38^. Our findings support and extend this concept by demonstrating that the filamentous fraction with the higher HA protein content has a distinct infectivity advantage in the presence of neutralizing IgG and, particularly, IgA antibodies (Fig. 4b–e), which are abundant in the upper respiratory tract where influenza virus primarily establishes infection. Using a cell-based microneutralization assay and primary human airway epithelial cell cultures, we further demonstrate that IAV preparations enriched in filaments are less susceptible to mucus restriction and exhibit higher virus titers in primary human airway cultures (Fig. 5a–d). This could be linked to a higher NA/HA ratio (Fig. 5g) and the asymmetric distribution of HA and NA on the viral surface, which facilitates more efficient penetration of the mucus layer via NA activity^39^. Together, our data suggest that differences in HA/NA content and ratio may underlie IAV shape-dependent infectivity during airborne transmission. Importantly, the significance of morphological variability would not be unique to IAV, as other virus families also exhibit pleomorphism and are airborne transmissible.

In this study, we used simple matrices to isolate individual protective or inactivating factors, allowing us to assess the effects of specific physical processes on viral inactivation. However, respiratory fluids and saliva in vivo contain a far greater diversity of components. Additional factors, such as salt crystallization, ionic strength, and the formation of a glassy shell by organics, further complicate aerosol microenvironments. To fully understand these effects, it is essential to integrate studies using both simplified and more physiologically relevant matrices, thereby providing a more comprehensive assessment of their role in IAV morphology-dependent airborne stability. An additional limitation of our work is the focus of our experimental system on small size aerosol particles. For comprehensive characterization future studies should also include experimental systems that produce and capture larger size aerosols. Furthermore, some studies suggest that IAV pH and salt stability are strain-dependent^63,64^. Additional research should explore the generalizability of these findings across different subtypes, particularly clinically relevant strains. Overall, our study reveals that filamentous shape of IAV may serve as a strategic adaptation, enhancing viral fitness during airborne transmission by increasing stability and infectivity under diverse extracellular pressures.

## Methods

### Cells

Human alveolar basal epithelial adenocarcinoma cells A549, Human embryonic kidney cells (HEK) 293T and Madin-Darby canine kidney (MDCK) cells were acquired from ATCC (cat #CCL-185, #CRL-11268 and #CCL-34, respectively) and maintained in Dulbecco’s Modified Eagle Medium (DMEM, Gibco) supplemented with 10% fetal calf serum (FCS, Gibco) and 1% penicillin-streptomycin (100 U/mL penicillin, 100 µg/mL streptomycin, Gibco) at 37 °C and 5% CO_2_.

Primary human nasal epithelial cells (NEpC, donor AB063, female, 41 years old, Caucasian, non-smoker) and bronchial epithelial cells (BEpC, donor AB079, male, 62 years old, Hispanic, non-smoker) were purchased from Epithelix (EP51AB). The cells were cultured in airway epithelium basal growth medium (#C-212601, PromoCell) supplemented with an airway growth medium supplement pack (#C-39160, PromoCell) and 10 µM ROCK inhibitor Y-27632 (#1251, Tocris). 12-mm filter inserts in Transwell plates (#CLS3460, Corning) were coated with 0.15 mg/mL collagen (#C7774, Sigma-Aldrich) in DPBS (Gibco). A total of 50,000 cells were seeded onto the coated transwell filters in a 1:1 mixture of airway epithelium basal growth medium and DMEM, supplemented with an airway growth medium supplement pack (Gray’s medium), and cultured until confluence was reached. To induce differentiation at the air-liquid interface (ALI), the medium in the apical compartment was removed, while the basal compartment was maintained with Gray’s medium supplemented with 150 ng/mL retinoic acid (#R2625, Sigma-Aldrich). Cells were cultured at ALI for a minimum of 28 days before use, with the medium in the basal compartment refreshed every 2 − 3 days. Epithelial integrity was monitored weekly by measuring transepithelial electrical resistance (TEER) using an ERS-2 meter (Millicell). For TEER measurements, Gray’s medium was temporarily added to the apical compartment. TEER values were normalized to the area of the transwell insert and expressed as area unit resistance (Ω·cm²). Cultures with a normalized TEER above 300 Ω·cm² were considered intact.

### Mucus

To collect mucus from BEpC and NEpC ALI cultures, 150 µL of sterile, ultrapure H_2_O was added to the apical compartment of 12 mm transwell inserts and incubated for 15 minutes at 37 °C. The resulting mucus–H_2_O mixture was harvested from each well, pooled by individual donor, and stored at −80 °C. Mucus samples collected sequentially at 4, 6, and 8 weeks of ALI culture were thawed, pooled per donor, aliquoted, and stored at –80 °C until further use.

### Plasmids and viruses

The plasmid pcDNA3.1-M1-Udorn-M2-WSN, which encodes M1 from influenza A/Udorn/307/72 (H3N2) (Udorn) and M2 from influenza A/WSN/33 (H1N1) (WSN), was described before^38,40^. This plasmid was used to generate the filament-producing strain A/WSN-M1udorn/33 (H1N1) (WSN-M1ud) within a WSN genetic background, as previously described^38,40^. Both the predominantly spherical parental WSN strain and the mutant filamentous-producing WSN-M1ud strain were rescued using reverse genetics, as described previously^65^. The plaque-purified viruses were passaged twice in MDCK cells with a multiplicity of infection (MOI) of 0.001 and titered by standard plaque assay. WSN viruses were stored at −80 °C, while WSN-M1ud viruses were snap-frozen in liquid nitrogen before being transferred to −80 °C for storage. The correctness of stock viruses was confirmed by sequencing.

### Virus concentration and purification

For preparations of the virus pair (WSN, WSN-M1ud), virus-containing supernatant from infected MDCK cells was first clarified by low-speed centrifugation to remove cell debris, followed by concentration using Amicon Ultra-15 PLHK Ultracel-PL Membrane 100 kDa tubes (UFC910024, Millipore) according to the manufacturer’s instructions.

Virus filament enrichment was performed as previously described^37^, with modifications. Briefly, WSN-M1ud supernatant from infected MDCK cells was first clarified by low-speed centrifugation to remove cell debris before virions were purified through a 30% sucrose cushion. An aliquot of unfractionated WSN-M1ud was then set aside before purification of viral fractions continued. Virions were subjected to eight cycles of centrifugation at 3250 × *g* for 1.5 h at 4 °C. Supernatants collected from all eight centrifugation cycles were pooled and further concentrated using Amicon Ultra-4 PLHK Ultracel-PL Membrane 100 kDa tubes (#UFC810096, Millipore), yielding the SUP fraction. The filament-enriched fraction (PEL fraction) was obtained from the pellet. Viral fractions were sonicated in an ultrasonic bath (Sonorex Super RK 52 H, Bandelin Electronic) three times for 10 s each to ensure a homogeneous suspension before aliquoting. All virus fractions were snap-frozen in liquid nitrogen and stored at −80 °C until further use. Sonication was also performed prior to each experiment.

### Virus infection

Infections for multicycle growth in MDCK cells were conducted in 6-well plates at an MOI of 0.001. The viral inoculum, prepared in PBSi (Dulbecco’s PBS supplemented with 0.3% BSA [#A7906, Sigma-Aldrich], 1 mM Ca²⁺/Mg²⁺, and 1% penicillin–streptomycin), was added to pre-washed cell monolayers and removed after a 1 h adsorption period. Cells were then washed three times with DPBS to remove the unbound virus. Subsequently, 2 mL of infection medium (Opti-MEM; #31985047, Thermo Fisher), supplemented with 1% penicillin–streptomycin, were added to each well. The cells were incubated at 37 °C, and supernatants were collected at the indicated time points for analysis by plaque assay.

Infections with unfractionated, SUP, and PEL fractions of WSN-M1ud in differentiated human primary epithelial cells cultured at ALI were carried out at 37 °C. ALI cultures aged 4 to 6 weeks were either apically washed with DPBS to remove accumulated mucus or left untreated. After a 3-day incubation at 37°C, the pre-washed wells were washed a second time with apical DPBS to remove residual mucus prior to infection. The basal medium was refreshed and maintained throughout the experiment. A viral inoculum with 7.5 × 10^6^ PFU prepared in PBSi, was applied to the apical surface of the cultures and incubated at 37 °C for 1.5 h. Following adsorption, all inserts were gently washed three times with warm DPBS to remove residual inoculum. At 3-, 8-, 16-, and 24-h post-infection, virus samples were collected by performing apical washes with DPBS, incubating for 15 min at 37 °C. The resulting virus–DPBS mixtures were harvested and stored at −80 °C. Infectious virus titers were determined by plaque assay on MDCK cells. The limit of detection (LoD) for the assay was 100 PFU/mL.

### Immunofluorescence

MDCK cells were seeded on coverslips in 24-well plates and infected at an MOI of 3. At 8.5 h post infection, cells were washed three times with DPBS and fixed with 3.7% paraformaldehyde (PFA, #15710, Lucerna) for 15 min. Cells were not permeabilized but blocked with 3% BSA in DPBS for 1 h. Following three washes with DPBS, samples were incubated with primary antibody anti-IAV H1 (WCL50, 1:10^66^) and secondary antibody Donkey anti-Mouse IgG (H + L) Highly Cross-Adsorbed Secondary Antibody, Alexa Fluor 488 (# A-21202. 1:1000, ThermoFisher Scientific) for 1 h. Samples were mounted with ProLong Gold Antifade Mountant (#P36930; ThermoFisher Scientific) and imaged with a confocal SP8 microscope using a 63x objective (Leica) or DMIL LED fluorescent microscope (Leica). A Z-stack series was collected, and 3D reconstruction was assembled using Imaris software.

### Flow virometry

Flow virometry was performed using a BD FACSymphony™ A1 Cell Analyzer (BD Biosciences) equipped with a small particle detector. Instrument performance was verified prior to acquisition using 90 nm NIST-traceable beads (#3090 A, Thermo Fisher Scientific) and Megamix-Plus SSC beads (#7803, BioCytex). Threshold and voltage settings for virus and calibration bead detection were optimized according to the manufacturers’ guidelines for small particle analysis.

Virus preparations were first sonicated and titered on the flow cytometer to determine the appropriate dilution yielding an acquisition of less than 20,000 total events per second, as suggested by the manufacturer. The anti-H1 HA WCL50 antibody specific for the WSN strain was purified using magnetic protein A beads (# L00695-4, GenScript) and conjugated to Alexa Fluor 488 (#A88062, ThermoFisher) following the manufacturers’ instructions. The conjugated antibody was used at a final dilution of 1:500 in HNE20 buffer (20 mM HEPES-NaOH, pH 7.4; 150 mM NaCl; 0.2 mM EDTA).

Virus samples were diluted in HNE20 buffer and mixed 1:1 with the antibody solution. Labeling was performed at room temperature for 1.5 h. Fluorescently labeled viruses were transferred to 5 mL polystyrene round-bottom tubes and analyzed by flow virometry. The A/Tasmania/503/2020 (H3N2) strain, which does not bind the anti-H1 antibody, served as negative control. Samples were acquired for 1 min at a slowest flow rate tolerated by the instrument for measurement. H3 virions and buffer-only controls were used to define gates for Alexa Fluor 488–positive virion populations, based on fluorescence and small particle side scatter (SP SSC). HA-positive virions were acquired using BD FACSDiva software (v9.0.2). and quantified by FlowJo software (v10.10.0). Viral filaments were defined as virion population with scatter signals higher than that of the WSN population, which correlated with virions longer than 230 nm as measured by cryo-electron microscopy (described below). Of note, the gating strategy based on HA staining of a control virus led to a large number of particles, especially in the small size range, being excluded from the analysis (Figure S2). While this could potentially lead to an overestimation of filamentous particles in our preps, adjusting the gate in a way that includes more of the “missed” virions would come with the risk of including exosomes or virus particles with undetectable levels of HA incorporation. Given that such particles would not be infectious we opted for the chosen HA-based gating strategy. Furthermore, quantification of shape by EM validated the quantification of shape by flow virometry, thus supporting the gating strategy.

### Quantification of IAV particle length using cryo-electron microscopy

To validate the morphological characterization of purified IAV obtained by flow virometry, virion length was analyzed for the three conditions WSN-M1ud unfractionated, SUP, and PEL, using cryo-electron microscopy. For each virus preparation, protein A-conjugated colloidal gold particles (Aurion, 10 nm diameter) were added as fiducial markers. Electron microscopy grids (Quantifoil R2/1, holey carbon film, Cu 200 mesh) were plasma-cleaned in a Solarus 950 (Gatan) prior to sample application. Plunge-freezing in liquid ethane was performed using an EM GP2 device (Leica) with the following settings: 3.5 µl sample volume, backside blotting for 1.5 s, 80% chamber humidity at 25 °C. Cryo-electron microscopy was performed using a Titan Krios transmission electron microscope (Thermo Fisher Scientific) operated at 300 keV and equipped with a BioQuantum LS energy filter with a slit width of 15 eV and a K3 direct electron detector (Gatan). Overview maps of grid squares were acquired at 8700x magnification with a pixel size of 10.64 Å/pixel and defocus of ~80 µm using SerialEM^67^. From these maps, virion lengths were manually measured in IMOD^68^ for at least 750 particles per condition. Additionally, tilt series were acquired at 33,000x magnification and pixel size of 2.671 Å/pixel and a defocus range of -2.5 and -4 µm using PACEtomo^69^. Projection images were collected following a dose symmetric tilt scheme in a tilt range between +60° and -60° in 3° increments and an approximate electron dose of 3.2 e^-^/Å^2^ per projection. Tomograms were reconstructed in Etomo^70^ with a pixel size of 5.342 Å/pixel.

### IAV inactivation assays

To determine the temperature stability of IAV, virus stocks were spiked into 350 µL of DPBS (#14040133, Thermo Fisher Scientific) to achieve an initial virus titer of 10⁷ PFU/mL and incubated in a Thermal Cycler (T3000, biometra) pre-heated to 24 °C or 55 °C for various times.

To assess the inactivation of IAV across a pH range from neutral to acidic conditions, Milli-Q H_2_O was adjusted to pH 7.0, pH 5.4, or pH 5.0 using a disodium phosphate (#71645, Sigma-Aldrich) and citric acid (#C1909, Sigma-Aldrich) buffer system, and to pH 10.0 using a sodium carbonate (#31432, Sigma-Aldrich) and sodium bicarbonate (#CS6014, Sigma-Aldrich) buffer system, as described by Sigma-Aldrich. The pH values were confirmed using an Ultra-Micro-ISM electrode and a FiveEasy pH meter (Mettler Toledo). For each reaction, 4 µL of virus stock were spiked into 96 µL of either neutral (pH 7.0) or acidic solutions, reaching a final concentration of 6 × 10⁶ PFU/mL. The mixtures were vortexed for ~5 s, and a 10 μL sample was taken immediately and neutralized in 190 μL of PBSi with 3.5 mM citric acid and 33 mM disodium phosphate (pH 7.0 PBSi). Further samples were collected and neutralized at specified time points post-exposure.

IAV inactivation in 26× PBS, 34× PBS, or 1× PBS bulk solutions (#524650, Millipore, in Milli-Q H_2_O) was performed at RT. Virus stocks (5 µL) were spiked into 495 µL of either 26× PBS, 34× PBS, or 1× PBS in 1.5-mL plastic tubes (#3080521, Sarstedt). and a 10 μL sample was taken every 2 h for 7 h in 270 μL or 350 μL of PBSi, followed by a final sample at 24 h. Virus titers of samples were quantified by plaque assay.

### Aerosol particle experiments: viral suspension preparation, aerosolization, exposure and sampling

A stock of 22 mL of PBS or synthetic lung fluid (SLF) containing 1 × 10^10^ – 4.5 × 10^11^ PFU of each individual virus was prepared in a 100-mL screw-capped container and kept at 4°C during preparation. SLF was prepared as described before^28,71^. SLF consists of Hanks’ balanced salt solution (HBSS) without phenol red, lyophilized albumin from human serum, human transferrin, 1,2-dipalmitoyl-sn-glycero-3-phosphocholine (DPPC), 1,2-dipalmitoyl-sn-glycero-3-phospho-rac-(1-glycerol) ammonium salt (DPPG), cholesterol, L-ascorbic acid, uric acid, and glutathione, which were all purchased from Sigma-Aldrich. Virus inoculum control was set by pipetting 25 μL of inoculated medium into 2.5 mL of PBSi and keeping it at 4°C in a 50-mL falcon tube over the course of the experiment. Virus-laden aerosol particles were produced by a bubble-bursting nebulizer (Sparging liquid aerosol generator, SLAG) for 1 or 1.5 min and exposed in a ~ 1.6 m^3^ aerosol chamber with controlled temperature and RH integrated inside a level 2 biosafety laboratory^50^. Particle-free air was filtered using class F9 HEPA filters Purified air was first cooled or heated to 21 °C and filtered through a classical particle filter (class F9). It was then further dried with drierite, and additionally passed through activated carbon and HEPA filters. This processed air, with or without the addition of HNO_3_, was then used to flush the chamber prior to each experiment. The chamber temperature was maintained between 23 °C and 25 °C during the experiments.

For aerosol sampling uing a BioSpot-VIVAS, aerosol particles were collected every 5 min in a Petri dish filled with 2.5 mL of PBSi. Each sampling cycle consisted of 4 min of collection followed by 1 min for Petri dish replacement. The air flow into the VIVAS was 8 L/min. For size-resolved aerosol sampling using an Andersen Impactor, 10 mL of PBSi was added to each of six glass Petri dishes, with one dish positioned beneath each of the six stages during impactor assembly. Virus inocula were nebulized for 1 min as described above, and aerosol particles were subsequently collected directly from the chamber into the Andersen Impactor for 10 min. The airflow rate through the Impactor was maintained at 28.3 L/min. Following collection, the liquid media from each stage was transferred into 50 mL falcon tubes and transported on ice for further processing. After each experiment, the chamber was decontaminated using UV radiation and ozone-rich air (6 ppm) for 1 h.

The infectious virus titer of each sample was determined by plaque assay. Total virus particle number was quantified by determining GC/mL by digital Polymerase Chain Reaction (dPCR).

### Particle size distribution measurements in the aerosol chamber

Particle size distribution was measured using a scanning mobility particle sizer (SMPS) and an optical particle counter (OPC). The SMPS consists of a differential mobility analyzer (DMA model TSI long, TSI Inc., Shoreview, MN, USA) and a condensation particle counter (CPC model 3772, TSI Inc., Shoreview, MN, USA). The OPC (model Met One HHPC 6 + , Beckman Colter Inc., Brea, CA, USA) is a 6-channel portable particle counter that counts particles between 0.3 and 10.0 μm optical diameter. The SMPS measuresd particles with diameters up to 760 nm. To extend this range, OPC measurements were performed; however, the OPC data utilize wider size bins. For instance, the first bin captures particles between 300 nm and 500 nm. To ensure continuity, the slope within each OPC bin was adjusted so that the distribution at the upper boundary of one bin matches the lower boundary of the subsequent bin.

### Quantification of IAV genome copies by dPCR

RNA was extracted by QIAamp Viral RNA Mini extraction kit (Qiagen, 52906) and eluted in 80 μL of elution buffer following manufacturer’s instructions. dPCR was performed using the QIAcuity Digital PCR System and QIAcuity OneStep Advanced Probe Kit (#250132, Qiagen) with the following primers targeting a 110-base amplicon of the IAV M segment (Microsynth):

Forward primer 5’- TGG AAT GGC TAA AGA CAA GAC CAA T-3’

Reverse primer 5’- AAA GCG TCT ACG CTG CAG TCC-3’

Probe- TTT GTK TTC ACG CTC ACC GTG CCC

A 12 µL single reaction mixture was prepared with 3 µL 4× OneStep Advanced Probe Mastermix, 0.12 µL 100× OneStep Advanced reverse transcriptase (RT) Mix, 1.2 µL Primer-Probe working solution (0.8 µM final concentration of each primer and 0.4 µM final concentration for probe), 1.5 µL Enhancer GC, 3.18 µL RNase-free water, and 3 µL of RNA sample diluted within dPCR detection range (<10^4^ copies/µL) or non-template control. The reactions were transferred into QIAcuity Nanoplate 8.5k 24-well or 96-well (#250011 or #250021, Qiagen) and dPCR was conducted by QIAcuity One Digital PCR System (#911020, QIAcuity one, 5-plex, Qiagen). The following conditions were used for the one-step cycling dPCR program: 40 min at 50 °C for reverse transcription, 2 min at 95 °C for enzyme denaturation, followed by 40 cycles of 95 °C for 5 s, then 58 °C for 30 s for annealing and extension. The absolute RNA concentration (copies/µL) was calculated using integrated QIAcuity Software Suite.

### Biophysical modeling

The Respiratory Aerosol Model ResAM is a biophysical model to determine virus inactivation in IRPs after exhalation as a function of air composition. ResAM is based on a spherical shell diffusion model, which embodies the thermodynamic and kinetic properties of respiratory fluids composed of H_2_O, H ^+^ , OH ^−^ , Na ^+^ , Cl ^−^ , CO_2_(aq), HCO_3_ ^−^ , NH_3_(aq), NH_4_ ^+^ , CH_3_COOH(aq), CH_3_COO ^−^ , CH_3_COONH_4_(aq), NO_3_ ^− ^, as well as two classes of organic compounds with low and high molecular weight, representative of the lipids and proteins in the lung fluid. For additional details on ResAM we refer to Luo et al.^28^.

### Microneutralization assay

A549 cells were seeded into clear, flat-bottom 96-well plates (TPP) and cultured until a confluent monolayer was established. For each virus strain, an MOI was selected to achieve ~90% infection, ensuring a robust signal for quantification.

For the antibody microneutralization assay, viruses were incubated for 1 h at RT in Opti-MEM with two-fold serially diluted S139/1 IgA (kindly provided by Prof. Ayato Takada, Hokkaido University, Japan) or WCL50 IgG antibodies targeting the H1 HA. As positive controls, viruses were mixed with an equal volume of Opti-MEM; Opti-MEM alone served as negative/mock control. The antibody–virus mixtures were added to pre-washed A549 cells and incubated for 1 h at 37 °C. After removal of the inoculum, infection medium (DMEM supplemented with 1% penicillin–streptomycin, 0.3% BSA, 20 mM HEPES [#H7523, Sigma-Aldrich], and 0.1% FCS) containing serially diluted antibodies was added to each well. Plates were incubated for another 6 h at 37 °C.

For the mucus microneutralization assay, equal volumes of virus were added to pre-warmed (37 °C), serially three-fold diluted mucus in Opti-MEM and incubated on ice for 1 h. Subsequent steps were carried out as described for the antibody microneutralization assay.

Following a 6 h incubation after inoculum removal, cells were washed with DPBS and fixed with 3.7% paraformaldehyde (PFA). Cells were then permeabilized and blocked for 1 h in confocal buffer (PBS supplemented with 50 mM ammonium chloride (#254134, Sigma-Aldrich), 0.1% saponin (#47036, Sigma-Aldrich), and 2% BSA). Infection was quantified by immunostaining for IAV NP using a mouse monoclonal antibody (HB65, #H16-L10-4R5, ATCC, 1:5) followed by Alexa Fluor-conjugated donkey anti-mouse IgG (H + L) secondary antibody (#A-21202, Thermo Fisher Scientific, 1:1000 diluted), both diluted in confocal buffer. Fluorescent signals were acquired using the IncuCyte S3 imaging system (Sartorius). To correct for background, the average infected cell area from mock-infected wells was subtracted from virus-infected wells, with negative values adjusted to zero. Relative infection was calculated by normalizing each condition to the average infection level of the positive control, representing maximal infection. For data analysis, inverse dilution factors of antibodies or mucus were log-transformed and plotted on the x-axis. A non-linear regression model was applied for curve fitting. To enable log-transformation, *x*-values for positive controls (i.e., without added mucus or antibody) were set to values 100-fold higher or lower than the highest matrix dilution or lowest antibody concentration, respectively. The regression curve was constrained with the maximum infection level (y) set to 1 and the baseline fixed at 0.

### Mucus penetration assay

To evaluate the ability of virus particles to diffuse through the mucus layer, 30 μL of either human respiratory mucus or PBS (control) was added to the apical chamber of 6.5-mm transwell inserts (0.4μm pore size, 0.3 cm² surface area; Sarstedt) placed in a 24-well plate. The basal compartment was filled with 400 μL of PBS. Subsequently, 3 μL of virus inoculum (3 × 10¹¹ GC/mL) was gently overlaid onto the mucus or PBS surface. The plate was incubated at 37 °C, and 50 μL samples were collected from the basal chamber at 30-, 60-, 120-, and 180-min. Viral genome copy concentrations in these samples were quantified using digital PCR (dPCR).

### Immunoblotting

Virus stock samples with the same genome copy number were lysed in 1x Laemmli buffer (1610747, Bio-Rad) supplemented with 10 % β-mercaptoethanol (M6250, Sigma-Aldrich). Samples were incubated at 95 °C for 5 min and loaded onto Bolt™ 4-12% Bis-Tris Plus Mini Protein Gels (NW04125, ThermoFisher Scientific). Proteins were separated by SDS-polyacrylamide gel electrophoresis (SDS-PAGE) and transferred onto 0.45 µm nitrocellulose membranes (10600008, Amersham). After blocking in 5% milk (T145.3, Roth) in Tris-buffered saline (TBS) containing 0.1 % Tween20 (93773, Sigma-Aldrich) (TBS-T) at RT for 1 h, membranes were probed with primary antibodies against IAV H1 (WCL50, 1:5), NA (#PA5-32238, ThermoFisher Scientific, 1:1000), and NP (rabbit polyclonal, 1:10000, kind gift of J. Pavlovic, Institute of Medical Virology, Zurich, Switzerland) in TBS-T overnight at 4 °C. Membranes were subsequently washed three times in TBS-T and probed with secondary antibodies (IRDye 800CW Goat anti-Mouse IgG (H + L) (LiCor, #926-32210), IRDye 680RD Goat anti-Rabbit IgG (H + L) (LiCor, #926-68071)) with dilution of 1:5000 in PBS-T at RT for 1 h. Signal was imaged with the Odyssey Fc Imager (Li-Cor) and quantified with Image Studio Lite (Version 5.2, Li-Cor).

### NA activity assay

NA activity was measured using the NA-Star Influenza Neuraminidase Inhibitor Resistance Detection Kit (#4374348, Thermo Fisher Scientific) following the manufacturer’s instructions, as previously described^53^. Virus stocks were diluted to 10⁷ PFU/mL in DPBS, and 10 µL of the virus dilution were added in triplicate to a 96-well plate (#CLS-3917, Costar) containing 40 µL of NA-Star assay buffer. As a negative control, DPBS was diluted 1:5 in the same assay buffer. Subsequently, 10 µL of the diluted NA-Star substrate was added to all wells. After 20 min of incubation at room temperature, 60 µL of the NA-Star accelerator was added, and luminescence was recorded using a 2140 EnVision multilabel plate reader (PerkinElmer). Signals from virus-containing wells were normalized to the negative control and then further normalized to the genome copies per well.

### Reporting summary

Further information on research design is available in the Nature Portfolio Reporting Summary linked to this article.

## Supplementary information

Supplementary Information

Reporting summary

Transparent Peer Review file

## Source data

Source data

## Data Availability

All data supporting the findings of this study, except for the electron microscopy images and tomograms, are available within the paper and its supplementary information files. Source data are provided with this paper. Cryo-EM overview maps used for morphology quantitative analyses presented in Fig. 1I have been deposited in Figshare: (10.6084/m9.figshare.31293784). (10.6084/m9.figshare.31293751). (10.6084/m9.figshare.31293721). Cryo-electron tomograms corresponding to Fig. 1k were deposited into EMDB under accession codes: EMD-56668 (Unfractionated); EMD-56664 (SUP); EMD-56667 (PEL). Source data are provided with this paper.
