# Supplementary material for: Filamentous morphology of influenza A virus confers enhanced stability in aerosols
Source: Nat Commun. 2026 May 15;17:6470. doi: 10.1038/s41467-026-73133-w (PMC13376898; doi:10.1038/s41467-026-73133-w)
Supplement: Supplementary file 1 — Supplementary Information [file 41467_2026_73133_MOESM1_ESM.pdf]

Supplementary figures

Filamentous morphology of influenza A virus confers enhanced stability in aerosols

Lu Liu[1], Ghislain Motos[2], Céline Terrettaz[2,3], Sarah Peterl[4,5], Josephine von Kempis[1], Marie O. Pohl[1], Umut Karakus[1], Elisabeth Gaggioli[1], Beiping Luo[6], Ulrich K. Krieger[6], Thomas Peter[6], Petr Chlanda[4,5], Tamar Kohn[3], Athanasios Nenes[2,7], and Silke Stertz[1*]

[1]Institute of Medical Virology, University of Zurich, Zurich, Switzerland

[2]Laboratory of Atmospheric Processes and their Impacts, School of Architecture, Civil & Environmental Engineering, École Polytechnique Fédérale de Lausanne, Lausanne, Switzerland

[3]Laboratory of Environmental Virology, School of Architecture, Civil & Environmental Engineering, École Polytechnique Fédérale de Lausanne, Lausanne, Switzerland

[4]Department of Infectious Diseases, Virology, Heidelberg University, Heidelberg, Germany

[5]BioQuant Centre for Quantitative Biology, Heidelberg University, Heidelberg, Germany

[6]Institute for Atmospheric and Climate Science, ETH Zurich, Zürich, Switzerland

[7]Center for the Study of Air Quality and Climate Change, Foundation for Research and Technology Hellas, Patras, Greece

* Corresponding author: Silke Stertz, Ph.D. (E-mail: stertz.silke@virology.uzh.ch)

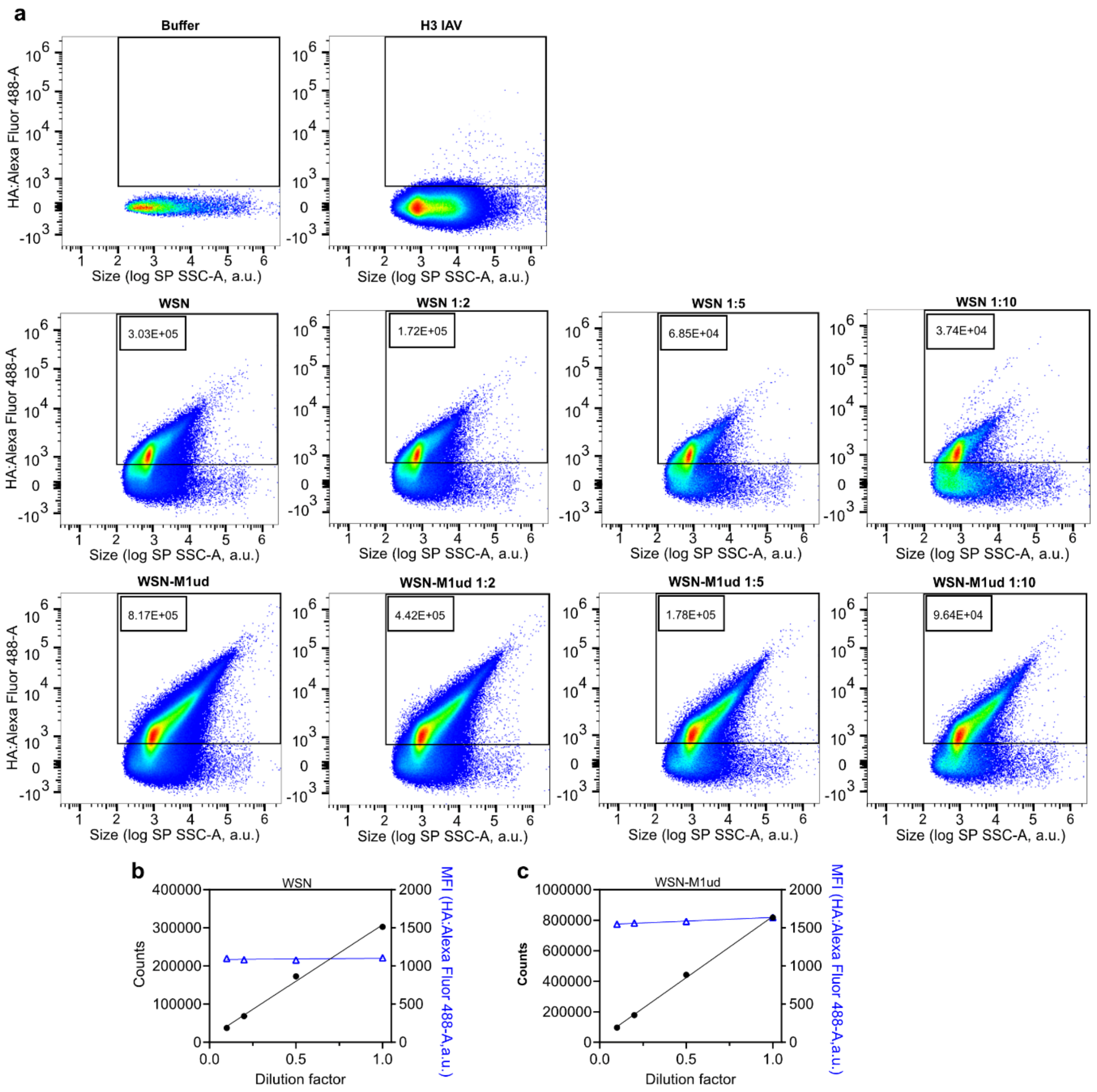

Supplementary Fig.1. Gating strategy and quality control of WSN and WSN-M1ud virions analyzed by flow virometry. (**a**) WSN and WSN-M1ud viruses were stained with an Alexa Fluor 488–conjugated anti-HA antibody specific for the WSN (H1) subtype. H3N2 virions incubated with the same antibody served as a negative control, additionally, buffer only samples were used to define background events. Fluorescence and small particle side scatter (SP SSC) signals were used to gate HA-positive virions (black frame). The same gating strategy was applied to serially diluted (1:2, 1:5, 1:10) samples. Denoted virion counts correspond to events in the gated region, acquired for 1 min. One representative experiment out of two independent experiments is shown. (**b**)–(**c**). Plot of serial virus dilutions of WSN (**b**) and WSN-M1ud (**c**), showing counts (left axis) and median fluorescence intensity (MFI) of Alexa Fluor 488 (right axis) as a function of the dilution factor. Values were derived from the gated population shown in (**a**). Source data are provided as a Source Data file.

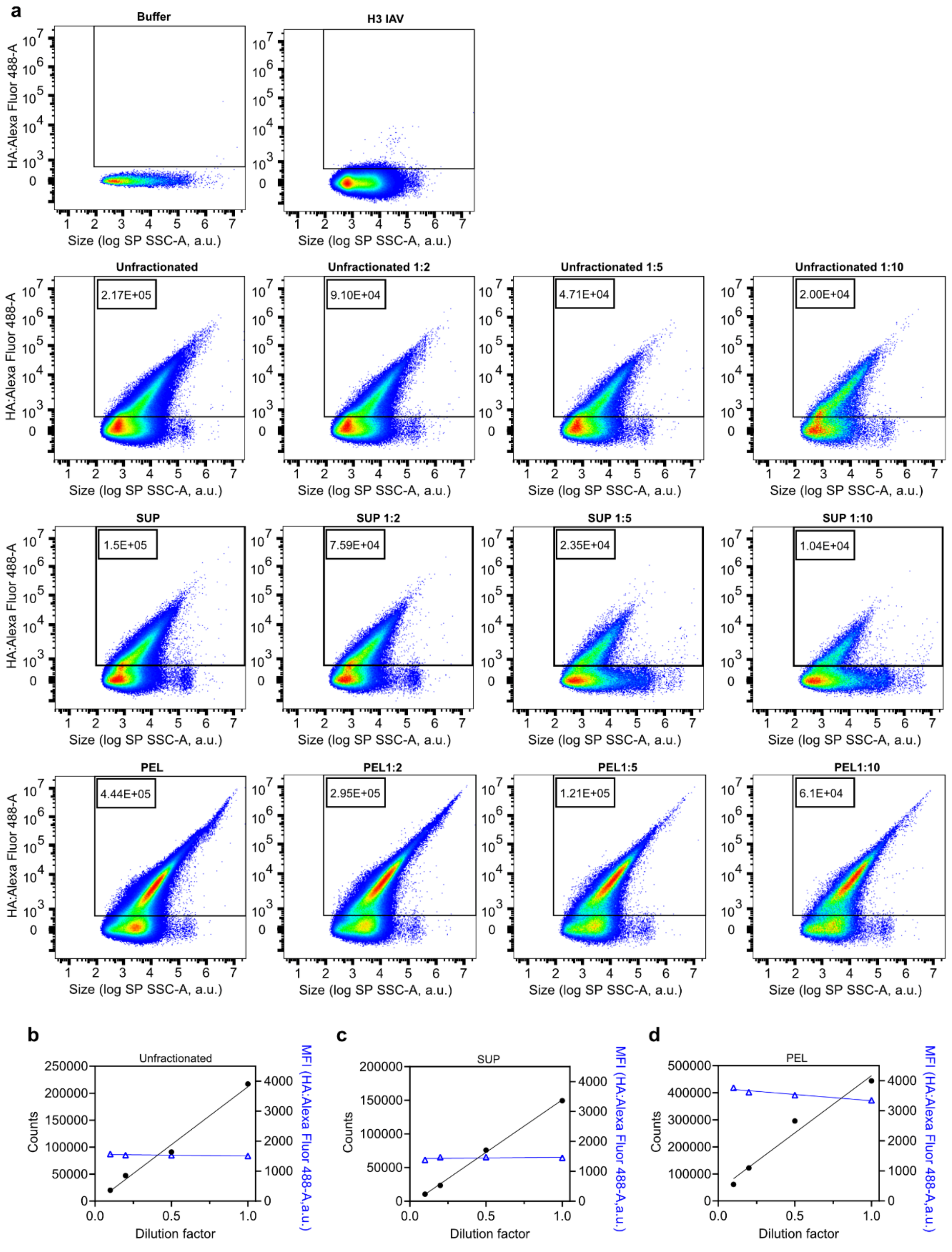

Supplementary Fig.2. Quality control of WSN-M1ud viral fractions in flow virometry. Swarm detection assays were performed using the same procedure as in Supplementary Fig. 1a. (**a**) Scatter plots of SP-SSC versus fluorescence for WSN-M1ud unfractionated, SUP, and filament-enriched PEL fractions, with gating of Alexa Fluor 488–labeled virions (black frame). Denoted numbers indicate virion counts in the gated region, acquired for 1 min. One representative experiment out of two independent experiments is shown. (**b–d**) Serial dilution plots of unfractionated (**b**), SUP (**c**), and PEL (**d**) fractions showing event counts (left axis) and median fluorescence intensity (MFI) as a function of dilution factor, derived from the gated populations in (**a**). Source data are provided as a Source Data file.

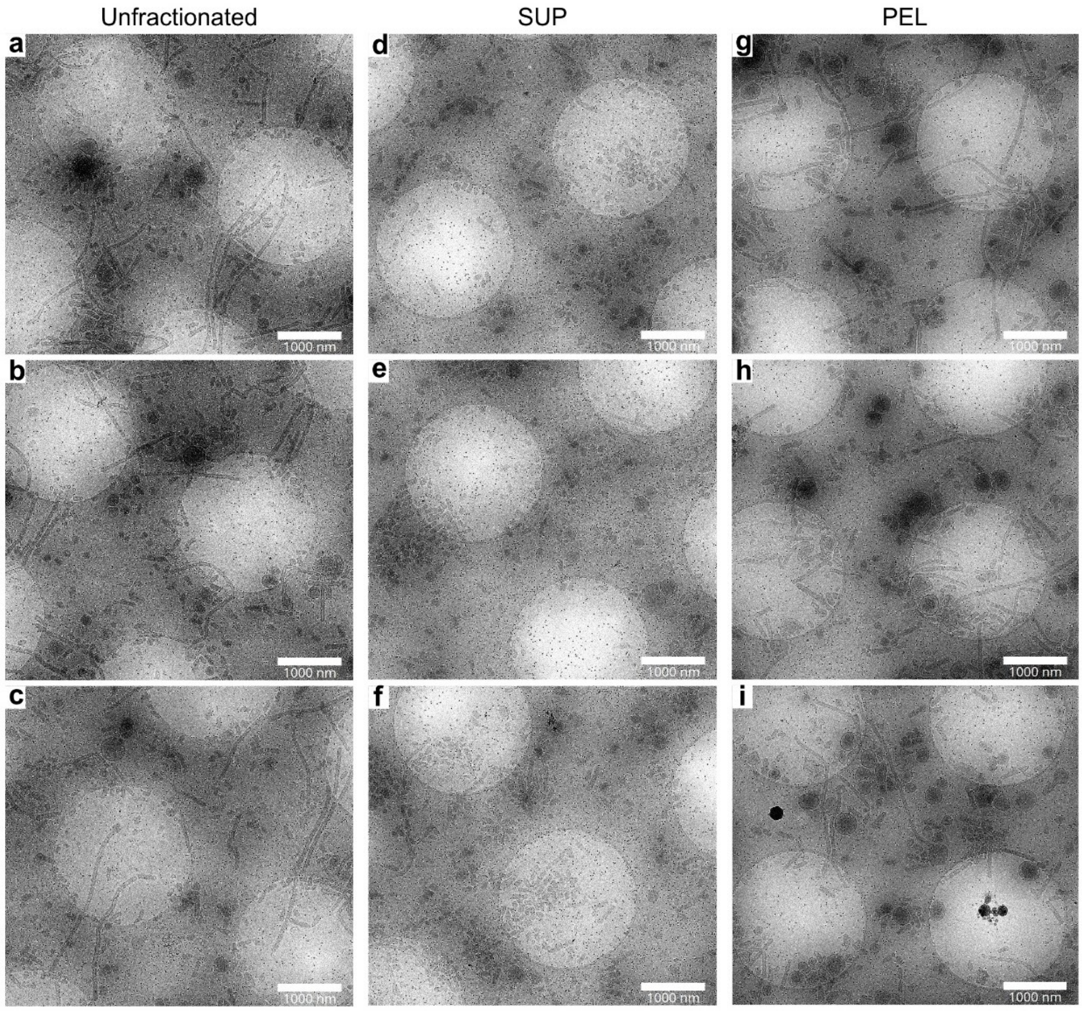

Supplementary Fig. 3. Representative cryo-EM maps used for quantification of virus shape distribution. Cryo-EM overview maps of WSN-M1ud unfractionated (a-c), SUP (d-f) and PEL (g-i) fractions. Scale bars: 1000 nm. Source data are provided as a Source Data file.

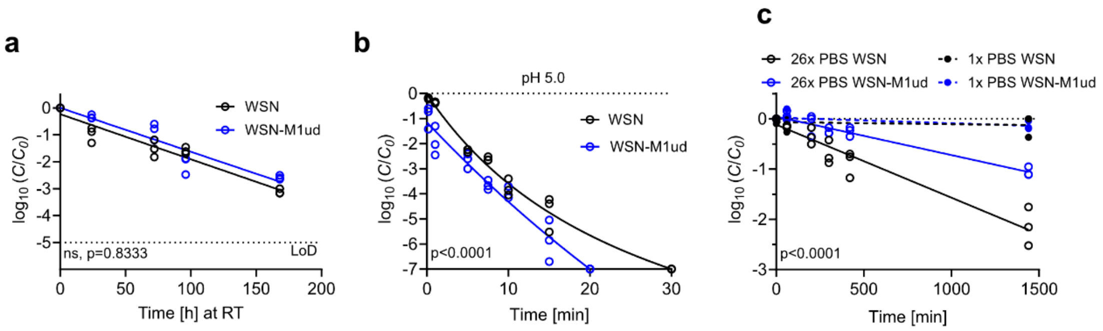

Supplementary Fig. 4. Infectivity decay for spherical and filament-producing IAV in bulk solutions under room temperature, acidic pH, and high-salinity (26× PBS) conditions. Experimental procedures were the same as described in Fig. 2. (**a**) log_{10} reduction in infectious virus titers of spherical WSN and filament-producing WSN-M1ud in PBS following incubation at RT (24 ± 1 °C) for the indicated time points over 7 days. (**b**) pH dependence: log_{10} reduction in infectious virus titers following exposure to aqueous citric acid/Na_2HPO_4 buffer adjusted to pH 5.0, normalized to the pH 7.0, 10 s sample. (**c**) Salt concentration dependence: log_{10} reduction in titers in 26× PBS or 1× PBS at the same initial virus titer normalized to their respective controls (after 10 s). (**a**–**c**) Data points represent independent experiments (n = 3), lines indicate mean values fitted with linear or nonlinear regression as appropriate. Statistical analyses were performed as described in Fig. 2; A p-value < 0.05 was considered statistically significant. Source data are provided in the Source Data file.

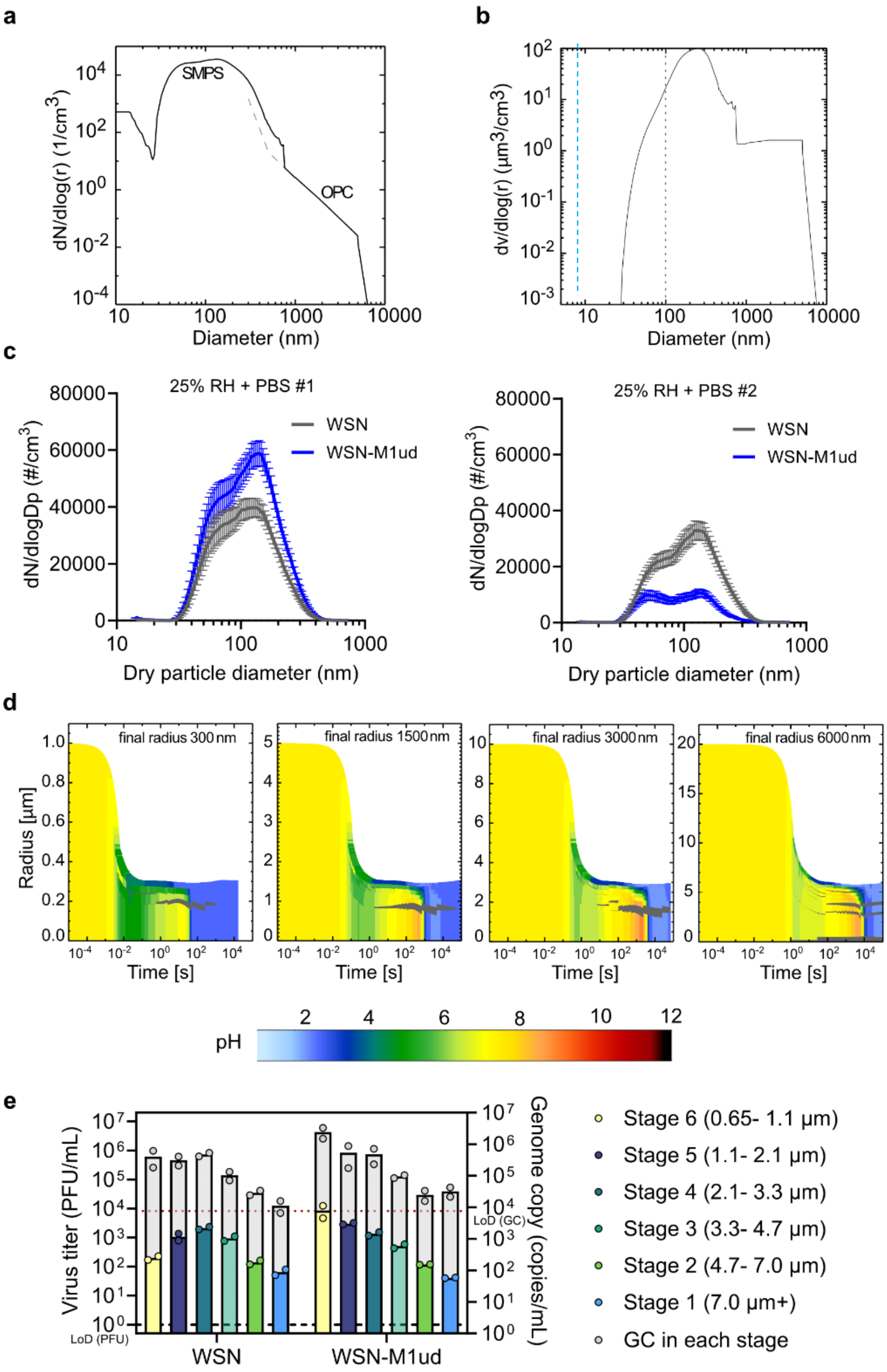

Supplementary Fig. 5. Size distributions of particles in the aerosol chamber. (a) Size distributions were characterized using both an SMPS (Scanning Mobility Particle Sizer) and an OPC (Optical Particle Counter). The SMPS measured particles with diameters up to 760 nm. To extend this range, OPC measurements were performed. The OPC data have wider size bins (for instance, the first bin captures particles between 300 nm and 500 nm, see dashed line). Therefore, we chose the slope within each OPC bin such that the distribution at the lower boundary of one bin matches the upper boundary of the subsequent bin, ensuring a continuous distribution. **(b)** Volume distribution as function of size corresponding to the number distribution in a. About 1.4 % of the total aerosol volume is in particles with diameter > 1 μm. Assuming viruses to be initially distributed volume-proportionally above 100 nm (blue dashed line), this results in about 1.5 % of all viruses that reside in supermicron particles. These particles are accessible to the virus measurements with the six-stage Andersen Impactor. **(c)** Particle number distributions of the two experiments described in Fig. 3e. The SMPS scanned every 4 min for a total of 32 min. **(d)** ResAM simulation of pH evolution in the interior of four SLF

particles with different initial sizes (1, 5, 10, 20 µm) exhaled into air with 25 %RH, 400 ppmv CO_2, 10 ppbv HNO_3 (without acetic acid, HCl or NH_3) at 25°C. **(e)** Virus inocula were prepared and nebulized as PBS particles into the aerosol chamber (for the experiment at 85 %RH described in Fig 3b) and then aerosol particles were fractioned by a six-stage Andersen Impactor straight after nebulization for a total of 10 min. The aerosol particle size cut-offs for stages 1 to 6 (top to bottom) are 7.0, 4.7, 3.3, 2.1, 1.1, and 0.65 µm, respectively. Each aerosol fraction was then analyzed in technical triplicates for infectious virus titers (PFU/mL) and total viral particle concentrations (GC/mL). Mean values are shown. The dotted lines indicate the limits of detection (LoD) of plaque assay (black) and dPCR (red). All experiments were repeated independently twice. Source data are provided as a Source Data file.

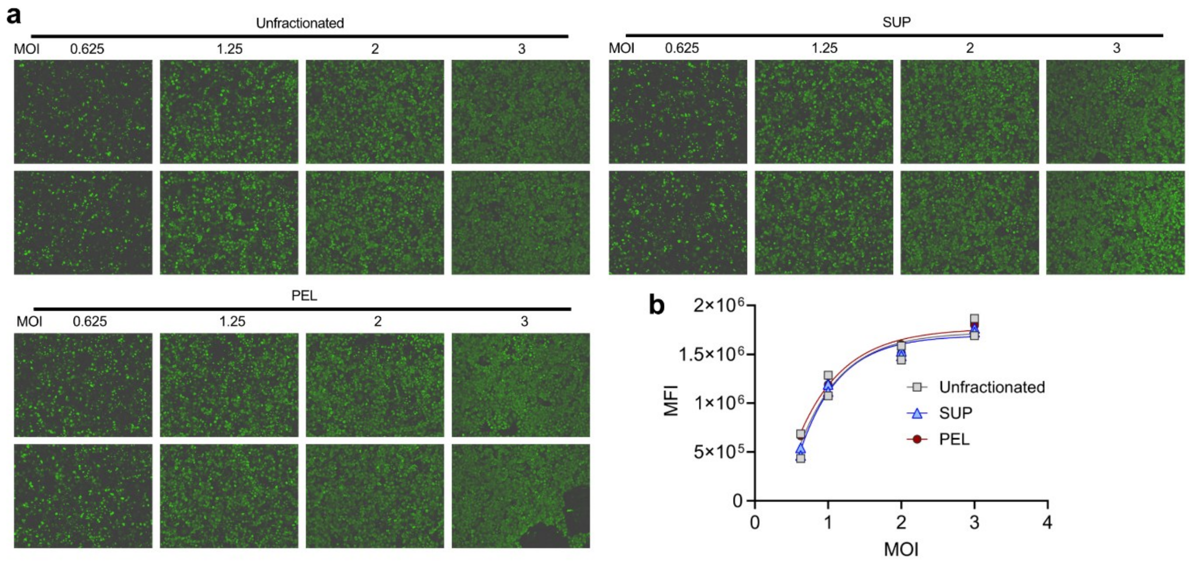

Supplementary Fig.6. Optimization of infection conditions for the microneutralization assay. (**a**) Fluorescence microscopy images of A549 cells infected in duplicate with increasing input amounts of WSN-M1ud viral fractions (MOI = 0.625, 1.25, 2, and 3) for 7 h. Infected cells were stained with an anti-IAV NP antibody and imaged using the IncuCyte S3 (Sartorius). One representative experiment out of two independent experiments is shown. (**b**) Corresponding mean fluorescence signal intensity (MFI) of cells analyzed with the IncuCyte S3 software. Source data are provided as a Source Data file.
