# Supplementary material for: Filamentous morphology of influenza A virus confers enhanced stability in aerosols
Source: Nat Commun. 2026 May 15;17:6470. doi: 10.1038/s41467-026-73133-w (PMC13376898; doi:10.1038/s41467-026-73133-w)
Supplement: Supplementary file 2 — Reporting summary [file 41467_2026_73133_MOESM2_ESM.pdf]

nature portfolio

Corresponding author(s): Silke Stertz, PhD

Last updated by author(s): May 4, 2026

Reporting Summary

Nature Portfolio wishes to improve the reproducibility of the work that we publish. This form provides structure for consistency and transparency in reporting. For further information on Nature Portfolio policies, see our Editorial Policies and the Editorial Policy Checklist.

Statistics

For all statistical analyses, confirm that the following items are present in the figure legend, table legend, main text, or Methods section.

n/a	Confirmed	
☐	☒	The exact sample size (*n*) for each experimental group/condition, given as a discrete number and unit of measurement
☐	☒	A statement on whether measurements were taken from distinct samples or whether the same sample was measured repeatedly
☐	☒	The statistical test(s) used AND whether they are one- or two-sided<br>*Only common tests should be described solely by name; describe more complex techniques in the Methods section.*
☒	☐	A description of all covariates tested
☒	☐	A description of any assumptions or corrections, such as tests of normality and adjustment for multiple comparisons
☐	☒	A full description of the statistical parameters including central tendency (e.g. means) or other basic estimates (e.g. regression coefficient) AND variation (e.g. standard deviation) or associated estimates of uncertainty (e.g. confidence intervals)
☐	☒	For null hypothesis testing, the test statistic (e.g. *F*, *t*, *r*) with confidence intervals, effect sizes, degrees of freedom and *P* value noted<br>*Give P values as exact values whenever suitable.*
☒	☐	For Bayesian analysis, information on the choice of priors and Markov chain Monte Carlo settings
☒	☐	For hierarchical and complex designs, identification of the appropriate level for tests and full reporting of outcomes
☒	☐	Estimates of effect sizes (e.g. Cohen's *d*, Pearson's *r*), indicating how they were calculated

Our web collection on statistics for biologists contains articles on many of the points above.

Software and code

Policy information about availability of computer code

Data collection	Leica LAS X software v3.0.2.16120, QIAcuity Software Suite v3.1.0.0, Incucyte 2022B Rev2, EnVision Manager v1.14.3049.528, BD FACSDiva software (v10.9.0)
Data analysis	ImageJ 1.54p, Bitplane Imaris v10.2.0, Maps offline viewer v3.28, GraphPad Prism 10.4.2, Li-Cor Image Studio Lite v5.2, FlowJo software (v10.9.0)

For manuscripts utilizing custom algorithms or software that are central to the research but not yet described in published literature, software must be made available to editors and reviewers. We strongly encourage code deposition in a community repository (e.g. GitHub). See the Nature Portfolio guidelines for submitting code & software for further information.

Data

Policy information about availability of data

All manuscripts must include a data availability statement. This statement should provide the following information, where applicable:

- Accession codes, unique identifiers, or web links for publicly available datasets
- A description of any restrictions on data availability
- For clinical datasets or third party data, please ensure that the statement adheres to our policy

All data supporting the findings of this study, except for the electron microscopy images and tomograms, are available within the paper and its supplementary information files. Source data are provided with this paper. Cryo-EM overview maps used for morphology quantitative analyses presented in Figure 1I have been

deposited in Figshare:
doi.org/10.6084/m9.figshare.31293784
doi.org/10.6084/m9.figshare.31293751
doi.org/10.6084/m9.figshare.31293721
Cryo-electron tomograms corresponding to Figure 1K were deposited into EMDB under accession codes: EMD-56668 (Unfractionated); EMD-56664 (SUP); EMD-56667 (PEL).

Research involving human participants, their data, or biological material

Policy information about studies with human participants or human data. See also policy information about sex, gender (identity/presentation), and sexual orientation and race, ethnicity and racism.

Reporting on sex and gender	*Use the terms sex (biological attribute) and gender (shaped by social and cultural circumstances) carefully in order to avoid confusing both terms. Indicate if findings apply to only one sex or gender; describe whether sex and gender were considered in study design; whether sex and/or gender was determined based on self-reporting or assigned and methods used. Provide in the source data disaggregated sex and gender data, where this information has been collected, and if consent has been obtained for sharing of individual-level data; provide overall numbers in this Reporting Summary. Please state if this information has not been collected. Report sex- and gender-based analyses where performed, justify reasons for lack of sex- and gender-based analysis.*
Reporting on race, ethnicity, or other socially relevant groupings	*Please specify the socially constructed or socially relevant categorization variable(s) used in your manuscript and explain why they were used. Please note that such variables should not be used as proxies for other socially constructed/relevant variables (for example, race or ethnicity should not be used as a proxy for socioeconomic status). Provide clear definitions of the relevant terms used, how they were provided (by the participants/respondents, the researchers, or third parties), and the method(s) used to classify people into the different categories (e.g. self-report, census or administrative data, social media data, etc.) Please provide details about how you controlled for confounding variables in your analyses.*
Population characteristics	*Describe the covariate-relevant population characteristics of the human research participants (e.g. age, genotypic information, past and current diagnosis and treatment categories). If you filled out the behavioural & social sciences study design questions and have nothing to add here, write "See above."*
Recruitment	*Describe how participants were recruited. Outline any potential self-selection bias or other biases that may be present and how these are likely to impact results.*
Ethics oversight	*Identify the organization(s) that approved the study protocol.*

Note that full information on the approval of the study protocol must also be provided in the manuscript.

Field-specific reporting

Please select the one below that is the best fit for your research. If you are not sure, read the appropriate sections before making your selection.

☒ Life sciences ☐ Behavioural & social sciences ☐ Ecological, evolutionary & environmental sciences

For a reference copy of the document with all sections, see nature.com/documents/nr-reporting-summary-flat.pdf

Life sciences study design

All studies must disclose on these points even when the disclosure is negative.

Sample size	No statistical methods were used to pre-determine sample sizes. Sample sizes were instead determined based on similar studies: DOI: 10.1128/mbio.03452-22; DOI: 10.3389/fmicb.2024.1484992
Data exclusions	No data were excluded from the analysis.
Replication	For all experiments, except for the aerosol chamber experiments, at least three independent experiments were done and in all cases results could be reproduced. For the aerosol chamber experiments two independent experiments were done for each condition tested and in all cases results could be reproduced. The number of repeats for each experiment is reported in the figure legends.
Randomization	Allocation of samples to groups was random.
Blinding	Investigators were not blinded to group allocation in this study as this was a discovery-based study.

Reporting for specific materials, systems and methods

We require information from authors about some types of materials, experimental systems and methods used in many studies. Here, indicate whether each material, system or method listed is relevant to your study. If you are not sure if a list item applies to your research, read the appropriate section before selecting a response.

Materials & experimental systems

n/a	Involved in the study
☐	☒ Antibodies
☐	☒ Eukaryotic cell lines
☒	☐ Palaeontology and archaeology
☒	☐ Animals and other organisms
☒	☐ Clinical data
☒	☐ Dual use research of concern
☒	☐ Plants

Methods

n/a	Involved in the study
☒	☐ ChIP-seq
☐	☒ Flow cytometry
☒	☐ MRI-based neuroimaging

Antibodies

Antibodies used

Commercial antibodies
- Polyclonal anti-influenza virus A/WSN/33 H1N1 NA antibody, Thermo Fisher Scientific, catalog# PA5-32238, used 1:1000
- Mouse monoclonal anti-NP antibody (hybridoma supernatant, HB-65, ATCC, catalog# H16-L10-4R5), unpurified hybridoma supernatant was used 1:5 diluted
- Donkey anti-Mouse IgG (H+L) Highly Cross-Adsorbed Secondary Antibody, Alexa Fluor™ 488, A-21202; Thermo Fisher Scientific, RRID: AB_141607, used 1:1000
- IRDye® 800CW Goat anti-Mouse IgG (H + L) Secondary Antibody, LiCor, catalog#926-32210, used 1:5000
- IRDye 680RD Goat anti-Rabbit IgG (H + L) Secondary Antibody, LiCor, catalog #926-68071, used 1:5000

Non-commercial antibodies
- Mouse monoclonal anti-A/WSN/33 HA WCL50 (clone H15-A13, PMID: 2414912), Dilution: unpurified hybridoma supernatant was used 1:10 diluted for iimunofluorescence, 1:5 for western blot or at indicated dilutions for neutralization assays
- Mouse monoclonal anti HA S139/1 IgA, kind gift of Ayato Takada, Hokkaido University, Japan, used at range of dilutions as indicated in figure legend
- Polyclonal anti-NP antibody, kind gift of J. Pavlovic, Institute of Medical Virology, Zurich, Switzerland, used 1:10'000

Validation

- The Polyclonal anti-influenza virus A/WSN/33 H1N1 NA antibody (Thermo Fisher Scientific, catalog# PA5-32238), the mouse monoclonal anti-NP antibody (hybridoma supernatant, HB-65, ATCC, catalog# H16-L10-4R5), the mouse monoclonal anti-A/WSN/33 HA WCL50 (clone H15-A13), the mouse monoclonal anti-HA S139/1 IgA, and the polyclonal anti-NP antibody (kind gift of J. Pavlovic) have been verified in previous related publications (PMID 2414912, PMID 35081340, PMID: 24465606, PMID 35867415)
- Secondary antibodies were tested by including controls without primary antibodies.

Eukaryotic cell lines

Policy information about cell lines and Sex and Gender in Research

Cell line source(s)	Human alveolar basal epithelial adenocarcinoma cells A549, Human embryonic kidney cells (HEK) 293T and Madin-Darby canine kidney (MDCK) cells were acquired from ATCC (cat #CCL-185, #CRL-11268 and #CCL-34, respectively). Primary human bronchial epithelial cell and nasal epithelial were purchased from Epithelix, Switzerland.
Authentication	MDCK cell lines were verified to be of canine origin by sequencing the COX1 transcript. No additional cell line authentication was performed.
Mycoplasma contamination	Cell lines used in the Stertz lab are routinely tested for mycoplasma contamination by PCR and none of the used cells here tested positive.
Commonly misidentified lines (See ICLAC register)	No commonly misidentified cell lines were used.

Plants

Seed stocks	*Report on the source of all seed stocks or other plant material used. If applicable, state the seed stock centre and catalogue number. If plant specimens were collected from the field, describe the collection location, date and sampling procedures.*
Novel plant genotypes	*Describe the methods by which all novel plant genotypes were produced. This includes those generated by transgenic approaches, gene editing, chemical/radiation-based mutagenesis and hybridization. For transgenic lines, describe the transformation method, the number of independent lines analyzed and the generation upon which experiments were performed. For gene-edited lines, describe the editor used, the endogenous sequence targeted for editing, the targeting guide RNA sequence (if applicable) and how the editor was applied.*
Authentication	*Describe any authentication procedures for each seed stock used or novel genotype generated. Describe any experiments used to assess the effect of a mutation and, where applicable, how potential secondary effects (e.g. second site T-DNA insertions, mosiacism, off-target gene editing) were examined.*

Flow Cytometry

Plots

Confirm that:

☒ The axis labels state the marker and fluorochrome used (e.g. CD4-FITC).

☒ The axis scales are clearly visible. Include numbers along axes only for bottom left plot of group (a 'group' is an analysis of identical markers).

☒ All plots are contour plots with outliers or pseudocolor plots.

☒ A numerical value for number of cells or percentage (with statistics) is provided.

Methodology

Sample preparation	Sample preparation is slightly different according to the starting material used and experiment performed. Please refer to the Methods section, where this is described in detail for each experiment.
Instrument	BD FACSymphony™ A1 Cell Analyzer
Software	BD FACSDiva software (v9.0.2), FlowJo software (v10.10.0)
Cell population abundance	Refer to methods section: no cell sorting was performed. Virus samples were prepared with appropriate dilution yielding an acquisition of less than 20,000 total events per second, at least 1200 total events per second.
Gating strategy	Gating strategies from main figures are shown in corresponding Supplementary figures. As a general strategy, virus population was defined using SP SSC-A/AF488-A. Within this population, gates for virion populations labeled with Alexa Fluor 488–conjugated HA were set using controls (buffer only with antibody control and antibody with A/Tasmania/503/2020 (H3N2) strain, which does not bind the anti-H1 antibody, served as negative controls).

☒ Tick this box to confirm that a figure exemplifying the gating strategy is provided in the Supplementary Information.
