# Supplementary material for: Filamentous morphology of influenza A virus confers enhanced stability in aerosols
Source: Nat Commun. 2026 May 15;17:6470. doi: 10.1038/s41467-026-73133-w (PMC13376898; doi:10.1038/s41467-026-73133-w)
Supplement: Supplementary file 3 — Transparent Peer Review file [file 41467_2026_73133_MOESM3_ESM.pdf]

nature portfolio

Peer Review File

Filamentous morphology of influenza A virus confers enhanced stability in aerosols

Corresponding Author: Professor Silke Stertz

This file contains all reviewer reports in order by version, followed by all author rebuttals in order by version.

Version 0:

Reviewer comments:

Reviewer #1

(Remarks to the Author)
Summary
This study investigates how influenza virus morphology, spherical versus filamentous particles, affects aerosol stability and infectivity of influenza virus. With the WSN strain and a WSN- M1udorn variant to represent spherical and filamentous forms respectively, the authors assess how each virus preparation withstands different temperature conditions, pH levels, solute concentrations and relative humidity conditions. They find that WSN particles are more stable at high temperature and low pH when compared to WSN-M1udorn particles, while WSN-M1udorn particles show increased stability in high salt concentrations and high humidity conditions. In human airway cultures initial comparisons between the WSN and the WSN-M1udorn variants show no differences in mucosal resistance. After the WSN-M1udorn is enriched for filaments through centrifugation the authors demonstrate that filamentous particles are more resistant to inactivation and retain higher infectivity under mucosal immune pressures. These findings support the hypothesis that IAV pleomorphism might offer advantages in specific environmental or physiological contexts, though key reservations to some of the interpretations are noted. This work has a potential to fill a notable gap in our understanding of the functional role of influenza pleomorphism or strain-to-strain variability in environmental persistence of influenza virus.

Overall Recommendation: Major revision needed

This study addresses an important question regarding the functional role of influenza virus morphology in its persistence in the environment. The results are novel and have the potential to advance our understanding of virus transmission and adaptation. However, the current manuscript overinterprets some findings and lacks key controls and structural context needed to fully support the conclusions. With major revisions including proper citation, improved morphological characterization/quantification and revised framing/wording this work could be appropriate for publication in Nature Communications.

General comments

1. While the M1 swap is intended to control for morphology, M1 influences many aspects of viral biology including mediate interactions with other viral or cell-membrane proteins/lipids, so virions produced from infections by WT WSN and WSN Udorn-M1 might differ in different aspects of their composition and not solely in shape. Without experiments that confirm that observed differences are solely due to morphology, the interpretation remains ambiguous. I suggest that either 1) key experiments be performed with shape-enriched preparations belonging to the same strain (also in Figures 1-3), or 2) conclusions for the properties ascribed solely to shape be qualified and alternative explanations to the results be discussed.
2. A lack of clear shape-dependent advantage of filaments over spheres in Figures 4-5 before additional enrichment is performed adds to the doubt that the initial comparisons were reporting on shape. See the preceding comment for alternative recommendations.
3. Several references are either incorrectly cited or used to support conclusions that the original studies do not directly address. See below for details.
4. Proper quantification of virion shape distributions from electron microscopy is needed in both Figures 1 and 4. IF images of several cells in Fig. 1, and which are likely to differ from cell-to-cell, are inadequate – they are not quantitative and do not report on the virus in their preparations. EMs in Fig. 4 are more helpful, but quantification is needed.
5. I suggest adding a structure showing where the amino acid differences between WSN and Udorn M1 lie within the known

structure of M1 to provide important insights into whether there are differences in other interactions that may underlie the stability differences.
6. The authors imply causal relationships between morphology and stability or infectivity where data supports only correlation. More qualified language would provide a more accurate interpretation of the results.

Line by line – (specific comments)

• Line 36, the words “genetically similar” are taken to mean ‘genetically identical’, as the authors go on to state that the WSN and the WSN-M1ud viruses differ only in morphology. We do not know this to be true, and even infectivity differences in Figure 1b argue to the contrary. They can differ in their structural properties and interactions with other viral or cellular proteins which could affect viral stability.
• Line 58-61 there is no evidence that shows that laboratory adapted IAV strains primarily produce uniform spherical virions and that pleomorphism is observed in clinical isolates. This statement has been repeated multiple times throughput the manuscript and it’s not substantiated.
• Line 61, citation 11 has been incorrectly cited. The paper studies virus from second egg and 3rd cell culture passage.
• Line 65, citation 18, this paper shows that M1 swaps change NA activity as well as shape, it is not clear whether it is the pleomorphism that led to increased transmissibility or if it is the changes in NA activity.
• Line 65- suggest changing the wording of the sentence, there is no causality established in the cited article.
• Line 92 citations should be listed in ascending order (12,14,28).
• Line 93, citation 29 asserts the opposite of what the authors state, in this citation spheres were preferentially released by NA inhibitor challenge.
• Figure 1b, do not need to add Udorn in the kinetics comparisons, WSN vs WSN-M1ud is enough.
• Line 165 – the findings can also be explained by the structure of the mismatched M1-HA, NA, NP virion being more labile in a way that is not related or exists in addition to morphology. Additionally, Udorn M1 could be less thermostable than WSN M1. I suggest discussing/acknowledging alternative explanations.
• Line 168 ResAM should be defined.
• Line 180, slower inactivation kinetics for WNS compared to WSN-M1-Udorn could be a result of M1-HA mismatch and not necessarily morphology.
• Line 252- pH sensitivity between different M1 variants can be different not necessarily because of shape.
• Figure 3d-e, not a paired experiment, suggest not connecting the dots on the graph.
• Figure 3f seems a bit high for the genome-particle ratio. Was RNase treatment added in this assay? It is not mentioned in the methods.
• Line 264-266, figure 3f – What is the actual size of particles in these droplets? Since no quantification of shape was done on these viral stocks it’s hard to draw the conclusion that the most filament containing viruses are in the smallest aerosol fraction, this is an assumption at this point.
• Line 287- does low-speed centrifugation cause aggregation? This can be hard to test but can be mentioned in the discussion. Check reference – don’t see that protocol in the cited paper.
• Line 290- Suggest adding quantification to know the degree of enrichment and the starting shape distributions (see comment above re: Figure 1).
• Figure 5a-b- for the neutralization assay, the MOI was set to achieve 90% infection, which too high. This will allow for significant coinfection making it hard to have a linear outcome between cells being infected and the virus being neutralized.

Reviewer #2

(Remarks to the Author)
The study is about influenza A virus, where one strain was modified to consist of both spherical and filamentous virus particles, and how the two different strains survive under various physicochemical pressures, i.e. high temperature, high salt, low pH and in aerosol phase. It is a nice study where the authors combined a variety of methods to explore virus inactivation in spherical and pleomorphic influenza, respectively. Aerosol experiments are lacking in literature, and the authors have created an impressive setup. The manuscript is well prepared with nice figures and text and I think the topic is very interesting, however, I have some comments that need to be addressed.
Considering the title, I find “airborne transmission” misleading. This is not a study where transmission is investigated. Your study evaluates virus survival and inactivation in an experimental setup.
In general, I am not convinced by the authors’ introduction and discussion about the low pH of exhaled aerosol particles. My interpretation is that there are conflicting perspectives of the pH of exhaled aerosol particles, what particle sizes that are relevant and on what (relevant) time scales pH changes take place. Your manuscript does not represent (or cite) other work than your own in a time when the scientific community does not agree. Your model results from previously are not what I would consider established facts, and therefore I strongly suggest that you broaden the picture of pH importance for respiratory aerosol particles, citing other groups performing research on the same or similar topic. For example, this publication indicates that in indoor air, there is no strong chemical driver to make an aerosol droplet at pH 7 change level of pH in indoor air without other strong sources: https://onlinelibrary.wiley.com/doi/full/10.1111/ina.12670 . Two of the co-authors of your manuscript have recently published an article showing that the pH increases shortly after exhalation, likely to pH ~10 (https://pubs.acs.org/doi/full/10.1021/acscentsci.5c00284). This is not clearly discussed in the present study, nor is a high pH evaluated in the experimental data.
Introduction:
I find two important errors in the introductory paragraph about pH. Exhaled aerosol particles do not become alkaline because of HCO_3 uptake, it is due to CO_2 evaporation in ambient air, which has lower CO_2 concentration than the air in our lungs.

You write about ”small particles” and “larger particles” as if they were defined; you need to be VERY specific here, because in aerosol science small particles can vary from 2 nm to 5 µm and large particles from 100 nm to 100 µm depending on whom you talk to.
During the covid pandemic, most exhaled viral RNA was found in particle sizes from 0.5-5 µm. These particles were likely around 2-20 µm as droplets. I would think that these are the important sizes to target for this type of research. Even though there might be viruses in even smaller particles <0.5 µm, these particles have limited ability to deposit in our respiratory system (ICRP model) and would therefore contribute with very little transmission of infection.
Would you please go through your text and verify that it is clear when you mean a wet aerosol particle (or a droplet) and when you mean a dry particle. I find it confusing sometimes when you refer to initial particle size - is that then the wet particle (droplet) size? Maybe there is nothing wrong, but I felt unsure in some places.
Figure 1c:
The micrographs showing stained cells, could you indicate in the small headings above the pictures that what is shown are “cells infected with WNS/Udorn/WSN-M1ud” instead of just the virus strains (for clarity for readers who are not used to interpret this type if images, or those who see it in grayscale).
Figure 2
Do you need to have different types of y-axes for the different graphs? For the C/C0 you measured PFU/mL, the same as in graph a and b, so could you have the same for c-f?
You use PBS as matrix for aerosolizing your virus – how is that related to your model results on pH for SLF?
Figure 3.
For the aerosol collection experiments, can you show the aerosol particle size distributions during each experiment? How stable is it within each collection time and between samples? You indicated earlier that you might have different protein concentrations in the liquids and this is something that could alter the size distribution and thereby what you collect, especially in the impactor. In any case, it is necessary to see how reproducible your aerosol concentration and size distribution is during your sampling times across the full size range that you collect (5 nm – 10 µm). You also need to show the difference between the aerosol size distributions with PBS and SLF, respectively. If they differ, it is also natural that your results do so, and then that could explain parts of the differences you show.
One way to clearly show the expected variations due to variations in the aerosol concentration is to determine the theoretically collected aerosol mass or volume from aerosol sizing instrument data (for example SMPS and APS) for each sample. This could then be included in your uncertainty and statistical calculations.
Since you have minutes on the x-axes for graphs a-c, can you also have minutes on the y-exes for d-e? It would help to have the same scale on a-c. As for now, it is difficult to compare a with b and c.
About the time resolution on your x-axis. If you sample for 10 min, then maybe you cannot give a sample just one given time point on the x-axis on a scale of minutes. How did you sample at all the time points given in Supplementary Fig.2? Was each sampling point from a new aerosolization? What is meant by “one experiment”? How does one sample at, for example 2.5 min when sampling takes 10 min? You write that “N0 is you first sampling time”. Does it take 10 min to collect that sample? Can you explain the jump from the first sample at time 0.0 (and how you sampled at t=0) to 2.5 min in Fig S2? I want to assure you that I very much appreciate that you include raw data like in S2, it is worth a lot for anyone who wants to compare! But maybe also make it shine through to Fig.3 as your divisions could look more representative of what they are, not starting from 0, but from -2 or what it actually is? It looks like there are error bars for some values but not for all? Why are copy numbers at the same level for both strains in one experiment S2a and S2d but not in S2b and s2c? I mean, if you look at your PFU/M1ud/25% you intend to do the exact same thing twice and end up with samples at the level of 10^4 one time and 10^2 another – how do you explain that? Even though your inactivation curves look nice, if you get this much variation that could be just chance? My apologies for a dozen questions here but this is crucial information and not clearly described.
In your previous publication in Frontiers, you have less than one order of magnitude inactivation within 45 min at low RH, here you have two orders of magnitude. Is it because of the different strains or what causes this difference?
Your conclusion here (fig.3), in the title and in the study in general, IAV has an advantage from its two morphologies which endure either high salt or acid conditions better. But if it is so that the pH becomes low in the aerosol particle, the virus would be exposed to both low pH AND high salt conditions, and would it then be so sure that having two morphologies is an advantage? You state this message in the title as well, and I am not sure this is what you show with your data. If the spherical ones inactivate at low pH and the filamentous at high salt concentration, then they would all be inactivated by the time someone else inhales them. I feel like you argue against yourselves.
Figure 4.
Impressive how well separated the spherical and filamentous viruses look from the electron microscopy images. Wouldn’t it be good if you stated if it is TEM or SEM?
Figure 5.
What does MFI stand for in the figure text? It is nice when one can understand as much as possible when looking at the graphs without reading the text – would it be possible to indicate in each graph with dilution factor (also fig.4) what was diluted (mucus, HA)? Some graphs have several markers for each x-value and some have one x-value with error bars – can you do the same? How many replicates do you have for the ones with error bars?
Did you ever try to aerosolize the two fractions SUP and PEL? I understand it is challenging to get enough material for it, but it feels like you could get more convincing data of any differences that exist.
Minor comments:
Row 73: “Elevated acidity”? Isn’t acidity enough?
Row 74: Is there nobody else to cite but your own group regarding influenza in aerosol particles?
Row 221: Elevated levels of proteins compared to what? And what is the total protein concentration?
Several places: “an” MOI instead of “a”.
Row 382: what is the buffering capacity of PBS in this context?
Row 397: where would they be if not in the liquid phase of a droplet? Protruding out in the air?
Row 409: You write that you expect organic compounds to form a liquid coating around the salt particle – on what do you

base this? From electron microscopy images with composition analysis that I have seen, there is no indication of this. There is no cited study either, is it just an assumption? It sounds wild to me.
Row 598: missing punctuation
SLF recipe. There are different recipes for synthetic lung fluid. Can you motivate your choice?
In general, I find it hard to differentiate between the different lines and markers in the some of the graphs because the line goes through the hollow ones filling them. Could you choose other shapes to facilitate interpretation?

Reviewer #3

(Remarks to the Author)
I co-reviewed this manuscript with one of the reviewers who provided the listed reports. This is part of the Nature Communications initiative to facilitate training in peer review and to provide appropriate recognition for Early Career Researchers who co-review manuscripts.

Version 1:

Reviewer comments:

Reviewer #1

(Remarks to the Author)
This study addresses an important question about the functional role of influenza virus morphology in its persistence in the environment. The results are novel and have the potential to advance our understanding of virus transmission and adaptation. The revised manuscript is a substantial improvement from the previous version. The inclusion of the experiments with shape enriched virus populations of the same strain adds important rigor to the study. Additionally, assessment of virion shape by both flow virometry and electron microscopy provides greater confidence in the findings. Despite the key improvements, detailed below are several remaining comments regarding the morphological analyses, use of citations, and statements of conclusions.
General comments
1. Flow virometry clearly shows size difference between WSN and WSN-M1ud and size enrichment in different WSN-M1ud fractions. However, the details of the quantification do not seem convincing:
a. For many of the flow traces shown in figures S1 and S2, the virus populations are not clearly resolved from the noise. A subset of spherical virions is likely being excluded from the quantification; the problem is worse for some samples than others – in the unfractionated samples, the spherical peak is almost entirely gated out, and in the SUP fraction, it is sliced through the middle by the gate. The authors should address this limitation as it affects the value for the fraction of filaments (exaggerates their prevalence) and has a consequence on where the true filament gate likely lies. While flow data confirms that different fractions are enriched in different size particles, the quantification by flow is not of sufficient quality to quantitatively compare to EM. This makes EM analysis quite important and raw data should be provided.
b. The current filament gating strategy assumes that WSN virions are entirely spherical, but data in Fig. 1d and S1 reveal two subpopulations, and S1 shows a diagonal tail radiating from the tight peak with greater fluorescence and scatter values. Since cutting off of a portion of the spherical peak due to poor separation from the noise (in S1) would increase the apparent fraction of larger particles (see preceding comment), the ‘real’ gate position might be after the main peak and before the small shoulder. Shifted gate would also make sense for the SUP data, which are quite different from the WSN virus – the small WSN ‘shoulder’ is a major subpopulation in the SUP, yet most of it is excluded from the current filamentous gate.
2. Explain the protocol modification that was done to increase the fraction filamentous in the WSN-M1ud virus – is this the amicon concentration step mentioned in the methods? If so, it is unclear how passage through a 100kDa pore would selectively remove spheres. Additional methodological detail is needed to explain how this step results in filament enrichment.
3. How does the dilution series test for aggregation? This approach may capture concentration-dependent aggregation, but it is unclear how it would distinguish irreversible aggregates from filaments.
4. Authors should include all EM images that went into quantification of morphology in figure 1 as raw data (currently only 1 image for the Unfractionated, SUP, and PEL are shown).

Minor comments
1. Some citations are incorrect or misrepresented and there is insufficient citing of previous literature:
a. Lines 104-106 -Make sure one of those references (12-14) includes ex vivo measurements to warrant the use of ‘primary’.
b. Lines 392-394 – “Initial tests with our virus the virus pair WSN and WSN-M1ud showed only minor differences in sensitivity to a HA-binding neutralizing antibody (IgG) (Fig. 4a), despite previous reports suggesting filamentous particles have an advantage” – Please change how this is explained - these are genetic variants and do not contrast studies performed on shape-enriched particles.
c. Lines 421-423 – “While viral NA activity correlates with mucus sensitivity, the association between viral shape and its ability to penetrate the mucus layer, along with the underlying mechanisms, remains poorly understood” - should cite Vahey and Fletcher on mucus penetration and filaments, directional motion.
• Michael D Vahey, Daniel A Fletcher (2019) Influenza A virus surface proteins are organized to help penetrate host mucus eLife 8:e43764
d. Lines 463-465 – “Given the higher increase in NA compared to HA these findings also support the concept that filamentous IAV with a higher NA:HA ratio may be more resistant to mucus inhibition” - seemingly contrary to what Vahey reported and should be discussed.
• Vahey, M. D. and D. A. Fletcher (2019). "Low-Fidelity Assembly of Influenza A Virus Promotes Escape from Host Cells."

Cell 176(1-2): 281–294 e219.
e. Lines 484-485 – "filamentous morphology that is genetically controlled by the M segment" - genetic control is versatile and not limited to M.
2. Lines 406-407 – "we also tested whether the reduced sensitivity of the filament-enriched fraction extends to IgA" - worth commenting that in the IgG experiment, sup is more sensitive and in the IgA experiment, pellet is more resistant (i.e. unfractionated tracks with the PELLET and SUP, respectively).
3. Please tone down overstating conclusions
a. Lines 566-568 – "Our findings support and extend this concept by demonstrating that the higher HA protein content of filamentous virus particles confers a distinct infectivity advantage in the presence of neutralizing IgG and, particularly, IgA antibodies" -- HA content as the cause was not explored - that seems likely, but it is not addressed by the current analyses.
b. Line 576 – "Together, our data provide a mechanistic understanding" – please tone down, current analyses do not address the mechanism though the findings are nonetheless relevant.

Reviewer #2

(Remarks to the Author)
Review of revised manuscript NCOMMS-25-40578A
Thank you very much for the revised manuscript. You have made impressive improvements with the additional experiments with SUP and PEL fractions investigated separately. Figures are much improved, as is the title.
The majority of my comments have been addressed and revised, however a few things remain. The major thing is about the acidity and particle size.
First, I find it unwise to use the term IRP (infectious respiratory particle) for anything else than the definition that is stated in the WHO document where it was recently defined (if you want to use the term). It is droplets from the respiratory tract containing pathogens and they can range from sub-micrometer to millimeter sizes. Thus, you cannot say that IRPs are the sub-µm and "large particles" is another size fraction. IRPs are all sizes.
Then we come to the size versus acidification in aerosol phase. You now state clearly at what time scales that particles of various sizes become acidic (pH 4), which I highly appreciate. However, if you have a 1 µm particle in initial/freshly exhaled size, it will become around 0.3 µm when evaporated at RH <75% in a few milliseconds. These aerosol particles have the size corresponding to the deposition minima in the respiratory tract, i.e. only about 10% will deposit (and mainly in the alveolar region). Most importantly, these particles contain 1000 times less volume than 3 µm particles that deposit to >95% in the respiratory tract. This means that you need to have way more (x10^4) particles of those sizes to actually make a difference.
The other size that you mention, 10 µm, should take days to become as acidic (pH 4) according to your model. The 10 µm initial droplet will dry to around 3 µm and carries a lot of volume (potential to carry more virus). According to your own experiments, amount of aerosol corresponds with virus genome copies, i.e. it is quite likely homogenously distributed in your aerosol particles (unless you would show something else). There might be a non-homogeneous distribution of virus in exhaled aerosol from humans, but in nebulized aerosol, it is most likely that it is homogenously distributed. Therefore, you should also have virus in particles in the µm-size range.
What I'm trying to get to, is that your focus on sub-µm particles and your model showing that they become very acidic very fast is not the full picture of relevance for aerosol transmission of virus. I believe that you have at 30% of mass above 0.8 µm that you do not measure. You might be theoretically right, but in this context, it is not fully relevant to focus only on 0.3 µm particles and ignore the larger ones that you choose not to measure.
You only plot your aerosol number distribution and not the mass or volume distribution (Suppl.fig.4a), and for homogenously distributed virus in particles, the volume distribution would be more accurate to show. Also, measuring only with the SMPS, that is limited to particles <0.8 µm and then stating that the majority of particles were of sub-µm sizes is a bit ridiculous. Fine, there are by number less particles above 0.8 µm, but they matter for the mass that you collect and for the particles that contain the virus that you present as your result (PFU). This you can clearly see in your collection with the Andersen impactor. You have a lot of viruses in the size range from 0.65-5 µm as you show here.
To conclude this big comment, I think that you should reframe the way you talk about particles of different sizes, about the rate of acidification in the context of virus-containing particles (to align with your experiment), and the relevance of acidification in normal indoor air.
And if you want to say that aerosol particles become acidified when you expose them to HNO_3, can you calculate the expected pH for the particle sizes corresponding to the Andersen impactor sizes as well?
Another thing on the topic. If the filamentous IAV are as long as 10 µm, how do they fit in the sub-µm particles? Do they wind up or shrink in some way?

Then there's the other thing about the SMPS measurements. The inter-run variability seems a bit high, varying a factor of 5 from #1 and #2 that you show in S.4a. Combined with the fact that you don't measure any particles larger than 0.8 µm, I don't think you can say that there's a distinct pattern difference in the Andersen impactor results, which I understand it as you ran it once for each virus strain. Limited aerosol particle monitoring during your experiments is a major flaw of the aerosol part of this study, especially since the data you show indicate quite some variability.

Minor comments
Row 44. Include statistics on this correlation.
Row 46. How acidified are particles of relevant particle size?
Row 74. Why did you choose reference no.23? Why not cite the actual original article that shows the data and science and not the bench version.
Row 365-367. As written above, this is a too strong statement considering the data you show.
Row 372. I don't agree that you expose aerosol to low pH until you have calculated the pH for Andersen-impactor-sizes.

Row 523-526. Again, this is theory but can you do the model calculations for relevant sizes and say the same?
Row 534. At RH below efflorescence, there can be a liquid phase from organics, but there shouldn't be any aqueous phase, right?
Row 542-545. It doesn't have to. This depends very much on the time scale that is relevant. So again, back to calculating the actual pH that is reached for 0.6-5 µm sizes and include those.

Reviewer #3

(Remarks to the Author)
I co-reviewed this manuscript with one of the reviewers who provided the listed reports. This is part of the Nature Communications initiative to facilitate training in peer review and to provide appropriate recognition for Early Career Researchers who co-review manuscripts.

Version 2:

Reviewer comments:

Reviewer #1

(Remarks to the Author)
Our positive overall assessment of the study and its impact we communicated previously stands. We further acknowledge the improvements in this round of revisions, but still have one major concern when it comes to the flow virometry and its interpretation. Basically, quantitative data for particle shape cannot be extracted from the flow data shown in Figure S2 and corresponding to the panels i, j, and l in Figure 1. Data presented in Figure S1 and corresponding to the panels e and f in Figure 1 do not suffer from the same shortcoming and seems fine to include. At most, the data shown in Fig. S2 support a qualitative conclusion of filament enrichment in the pellet fraction. Together with the provided EM data, it is sufficient to make the authors' claims as relevant for this study, but I would refrain from providing quantification or quantitative comparisons to the EM data. We suggest that the authors remove panels of Fig1. i, j, and the virometry quantification from panel l. We provide detailed explanation for this suggestion below.

The simple application of the same gating strategy across samples in Figure S2 where spherical population is not sufficiently pulling out of the noise definitely leads to an overestimation of filaments (and not only potentially, as indicated in line 671 of the revised manuscript). This is not to suggest that a different gate should be drawn because drawing it any lower would include noise (and lead to underestimating filaments), but to emphasize the limitation of those specific virometry measurements (Fig. S2) to derive quantitative value for shape distributions. A close look at the scatter plots shown in Fig. S2, especially for the unfractionated and the Sup fraction, reveals a virus subpopulation that is present but not entirely pulling out of the noise. This is not as evident in the pellet fraction, possibly because the signal is dominated by larger filaments. The lower relative inclusion of the spherical particle data can also explain the distributions shown in Fig. 1h where the virus peak for the unfractionated and supernatant fractions is shifted towards larger events and does not correspond with the sphere peak in WSN or WSN-UdM1 samples. Given these limitations, we do not think that quantitative values could be derived from the flow virometry data in Fig. S2.

Along the same lines, direct quantitative comparisons between flow virometry and EM are not appropriate in the context of Fig. S2 data. The claim that the missed virions have undetectable HA levels and thus are not infectious and can be disregarded is an assumption that is not substantiated by evidence. Smaller particles not pulling out from the noise could result from a technical issue like insufficient labeling antibody, higher noise level, fluorescent laser instability, trigger delay issues, etc. With respect to the virus structure, it could reflect epitope accessibility and not necessarily HA levels. Even if HA levels were lower on a subset of particles, sensitivity by virometry is not a measure of particle infectivity, and disregarding particles not revealed by virometry as not infectious is unjustified. Importantly, these missed virions are being counted in the EM quantification, so obtaining the same values is not expected, and rather than corroborating virometry, it further brings into question the gating strategy.

In conclusion, the authors should acknowledge that flow virometry is a quick readout that allows quick corroboration of filament enrichment in the pellet fraction, but in the case of provided data in Fig. S2, not a quantitative assessment since it excludes a major virus subpopulation.

Reviewer #2

(Remarks to the Author)
Great that you have added the volume distribution of your aerosol. Could you change to diameter instead of radius to be in line with your previous plots?

Highly appreciate the model figures being included now, it is more transparent to the reader. Although I am not convinced that this plays a major role for airborne transmission of influenza (considering the low lung deposition fraction of 0.3 µm particles).

Regardging the Andersen impactor measuremetns, did you not have a filter collecting the <0.65 µm particles? That would have shown real proof of your virus distribution.

Reviewer #3

(Remarks to the Author)
I co-reviewed this manuscript with one of the reviewers who provided the listed reports. This is part of the Nature Communications initiative to facilitate training in peer review and to provide appropriate recognition for Early Career Researchers who co-review manuscripts.

Open Access This Peer Review File is licensed under a Creative Commons Attribution 4.0 International License, which permits use, sharing, adaptation, distribution and reproduction in any medium or format, as long as you give appropriate credit to the original author(s) and the source, provide a link to the Creative Commons license, and indicate if changes were made.
In cases where reviewers are anonymous, credit should be given to 'Anonymous Referee' and the source.
The images or other third party material in this Peer Review File are included in the article's Creative Commons license, unless indicated otherwise in a credit line to the material. If material is not included in the article's Creative Commons license and your intended use is not permitted by statutory regulation or exceeds the permitted use, you will need to obtain permission directly from the copyright holder.
To view a copy of this license, visit https://creativecommons.org/licenses/by/4.0/

Liu et al., Response to reviewers' comments

We would like to thank the reviewers for their thoughtful suggestions for further improvement. We have addressed the comments from the reviewers point-by-point as outlined below (in blue). The line numbers given below refer to the manuscript version with marked-up changes.

Major Issues: Key Experiments Required for Acceptance

Reviewer #1:Summary

This study investigates how influenza virus morphology, spherical versus filamentous particles, affects aerosol stability and infectivity of influenza virus. With the WSN strain and a WSN-M1udorn variant to represent spherical and filamentous forms respectively, the authors assess how each virus preparation withstands different temperature conditions, pH levels, solute concentrations and relative humidity conditions. They find that WSN particles are more stable at high temperature and low pH when compared to WSN-M1udorn particles, while WSN-M1udorn particles show increased stability in high salt concentrations and high humidity conditions. In human airway cultures initial comparisons between the WSN and the WSN-M1udorn variants show no differences in mucosal resistance. After the WSN-M1udorn is enriched for filaments through centrifugation the authors demonstrate that filamentous particles are more resistant to inactivation and retain higher infectivity under mucosal immune pressures. These findings support the hypothesis that IAV pleomorphism might offer advantages in specific environmental or physiological contexts, though key reservations to some of the interpretations are noted. This work has a potential to fill a notable gap in our understanding of the functional role of influenza pleomorphism or strain-to-strain variability in environmental persistence of influenza virus.

Overall Recommendation: Major revision needed

This study addresses an important question regarding the functional role of influenza virus morphology in its persistence in the environment. The results are novel and have the potential to advance our understanding of virus transmission and adaptation. However, the current manuscript overinterprets some findings and lacks key controls and structural context needed to fully support the conclusions. With major revisions including proper citation, improved morphological characterization/quantification and revised framing/wording this work could be appropriate for publication in Nature Communications.

General comments

1. Without experiments that confirm that observed differences are solely due to morphology, the interpretation remains ambiguous. I suggest that either 1) key experiments be performed with shape-enriched preparations belonging to the same strain (also in Figures 1-3), or 2) conclusions for the properties ascribed solely to shape be qualified and alternative explanations to the results be discussed.

To address this point, we followed suggestion (1) and performed both, the bulk and aerosol-chamber experiments, using shape-enriched preparations of the same strain (WSN-M1udorn). These preparations were characterized with detailed morphological quantification using highly

sensitive flow virometry [1, 2]. The updated results are presented in the revised Fig. 1–3 and new Supplementary Fig. 1–2.

When using the shape-enriched fractions of WSN-M1udorn we found that for salt-mediated inactivation both systems lead to the same conclusion: Filamentous shape confers protection from salt-mediated inactivation. Thus, both viral systems can be used. However, for low pH-mediated inactivation the shape-enriched fractions revealed that filamentous shape also protects from low pH-mediated inactivation. This is in contrast to the results obtained with the virus pair (WSN/ WSN-M1udorn), leading us to speculate that the amino acid differences in M1 between WSN and WSN-M1ud not only affect viral morphology but also other viral traits that impact acid stability. Thus, using the shape-enriched fractions is the better system to study morphology-related differences. We believe that following this reviewer's suggestion has thus improved the manuscript substantially, and we thank them for their valuable input.

2. A lack of clear shape-dependent advantage of filaments over spheres in Figures 4-5 before additional enrichment is performed adds to the doubt that the initial comparisons were reporting on shape. See the preceding comment for alternative recommendations.

As mentioned above, we quantified virion shapes for both WSN and WSN-M1ud concentrated by ultrafiltration using flow virometry. These measurements confirmed that WSN-M1ud contains a higher proportion of filaments compared with WSN, although filament abundance in WSN-M1ud remained below 20%. We agree that this relatively modest difference may limit the ability to detect clear shape-dependent effects in Fig 4–5 prior to additional enrichment. This is why we performed subsequent shape-enrichment experiments, which enabled more robust comparisons and allowed us to better isolate morphology-associated phenotypes.

3. Several references are either incorrectly cited or used to support conclusions that the original studies do not directly address. See below for details.

We have carefully reviewed the referenced literature and corrected all citations accordingly. Details of the corrections are provided below.

4. Proper quantification of virion shape distributions from electron microscopy is needed in both Figures 1 and 4. IF images of several cells in Fig. 1, and which are likely to differ from cell-to-cell, are inadequate – they are not quantitative and do not report on the virus in their preparations. EMs in Fig. 4 are more helpful, but quantification is needed.

For quantification of virion shape distributions, we developed a quantitative flow virometry assay based on a recently published approach[1, 2] and validated this method by cryo-EM, shown in Fig.1d-l and Supplementary Fig. 1–2.

5. I suggest adding a structure showing where the amino acid differences between WSN and Udorn M1 lie within the known structure of M1 to provide important insights into whether there are differences in other interactions that may underline the stability differences.

We appreciate the suggestion to map the M1 amino acid differences onto the known structure. As noted in comment #1, we now rely mainly on purified shape-enriched WSN-M1ud fractions to examine morphology-related effects. Therefore, we have not included an M1 structural illustration but now clearly acknowledge in the text that M1-related viral traits may contribute to IAV acidic pH stability.

6. The authors imply causal relationships between morphology and stability or infectivity where data supports only correlation. More qualified language would provide a more accurate interpretation of the results.

We have gone through the text to modify the wording where necessary in response to this comment. Examples are in lines 234, 264, and 466-467.

Line by line – (specific comments)

7. Line 36, the words “genetically similar” are taken to mean ‘genetically identical’, as the authors go on to state that the WSN and the WSN-M1ud viruses differ only in morphology. We do not know this to be true, and even infectivity differences in Figure 1b argue to the contrary. They can differ in their structural properties and interactions with other viral or cellular proteins which could affect viral stability.

Thank you for this comment. We did not intend to imply that the WSN and WSN-M1ud viruses are genetically identical or that they differ exclusively in morphology. Our intention was to highlight that the two viruses are genetically similar and share the same genetic background, aside from the engineered differences in the M1 gene that drive their distinct morphologies. We fully agree that these M1 differences may also influence structural properties or interactions with viral or cellular proteins, which could in turn contribute to the observed infectivity and stability differences.

We have revised the wording throughout to avoid this confusion. The specific sentence referred to here has been cut from the abstract. Moreover, as the focus of the manuscript is now mainly on the comparison of the shape-enriched fractions, this is less of a problem now.

8. Line 58-61 there is no evidence that shows that laboratory adapted IAV strains primarily produce uniform spherical virions and that pleomorphism is observed in clinical isolates. This statement has been repeated multiple times throughput the manuscript and it’s not substantiated.

We have revised the text (lines 61–64, and 104) throughout the manuscript to clarify that pleomorphism and filament formation are more frequently observed in low-passage-number or primary clinical isolates, rather than implying a strict division. We have also added appropriate citations to support this statement and removed any instances where the language may have overgeneralized.

9. Line 61, citation 11 has been incorrectly cited. The paper studies virus from second egg and 3rd cell culture passage.

We thank the reviewer for pointing this out. We acknowledge that citation 11 was incorrectly cited in this context, as the referenced study used virus derived from the second egg passage and the third cell culture passage. We have reworded the text accordingly in lines 61–64.

10. Line 65, citation 18, this paper shows that M1 swaps change NA activity as well as shape, it is not clear whether it is the pleomorphism that led to increased transmissibility or if it is the changes in NA activity.

According to the reviewer’s comment we reworded in lines 67–69 to clarify that “*IAV filamentous morphology has been reported to be associated with enhanced viral infectivity and transmissibility in animal infection models*”.

11. Line 65- suggest changing the wording of the sentence, there is no causality established in the cited article.

According to the reviewer's suggestion we reworded in lines 67–69 to clarify that "*IAV filamentous morphology has been reported to be associated with enhanced viral infectivity and transmissibility in animal infection models*".

12. Line 92 citations should be listed in ascending order (12,14,28).

This has been corrected (line 106).

13. Line 93, citation 29 asserts the opposite of what the authors state, in this citation spheres were preferentially released by NA inhibitor challenge.

We acknowledge the mistake and have corrected the statement accordingly in lines 106–109.

14. Figure 1b, do not need to add Udorn in the kinetics comparisons, WSN vs WSN-M1ud is enough.

As suggested, we now only show WSN vs WSN-M1ud in revised Fig.1a–c.

15. Line 165 – the findings can also be explained by the structure of the mismatched M1-HA,NA, NP virion being more labile in a way that is not related or exists in addition to morphology. Additionally, Udorn M1 could be less thermostable than WSN M1. I suggest discussing/acknowledging alternative explanations.

We agree that M1 Udorn could influence thermostability. To address this, we now use shape-enriched fractions from the same strain (as described in response to comment #1) to study morphology-dependent thermostability, as shown in the new Fig. 2a–b.

16. Line 168 ResAM should be defined.

ResAM has been defined in the introduction section in lines 90-92.

17. Line 180, slower inactivation kinetics for WNS compared to WSN-M1-Udorn could be a result of M1-HA mismatch and not necessarily morphology.

We appreciate the reviewer's important point. As discussed in response to comment #1, we now use shape-enriched fractions of WSN-M1ud to study morphology-dependent acid stability in bulk (new Fig. 2c–d). Interestingly, the two viral systems show opposite trends in acid stability, which suggests that the amino-acid differences in M1 between WSN and WSN-M1ud may influence not only morphology but also additional viral traits affecting acid stability, as the reviewer noted. We have added this important point in lines 282–285 of the revised manuscript.

18. Line 252- pH sensitivity between different M1 variants can be different not necessarily because of shape.

We appreciate the reviewer's important point. This overlaps with comment #17. After comparing the new bulk pH-stability data from the purified WSN-M1ud fractions with our previous WSN vs. WSN-M1ud viral pair, we observed opposite trends in virus decay. This supports the conclusion that the purified WSN-M1ud fractions provide a more appropriate model for isolating shape-related effects during low-pH inactivation. For this reason, and because pH sensitivity differences between M1 variants cannot be attributed solely to morphology, we have not included the

comparison of WSN vs. WSN-M1ud in the aerosol chamber in the revised manuscript but focus on the shape-enriched fractions instead.

19. Figure 3d-e, not a paired experiment, suggest not connecting the dots on the graph.

As suggested, we have revised the figures and we no longer connect the data points. In the new Fig. 3b, 3f, and 3h, each independent dataset is displayed with consistent symbol shapes (either open circles or open triangles) without lines connecting them.

20. Figure 3f seems a bit high for the genome-particle ratio. Was RNase treatment added in this assay? It is not mentioned in the methods.

We did not perform RNase treatment of our virus stocks and the genome to particle ratio can vary between stocks. However, as shown in Supp. Fig. 4a and Fig. 3e (and discussed in more detail below in response to comment no. 8 from reviewer no. 2) we find that the decay rates we measure are robust even if input virus stocks differ in genome to particle ratio or other parameters.

21. Line 264-266, figure 3f – What is the actual size of particles in these droplets? Since no quantification of shape was done on these viral stocks it's hard to draw the conclusion that the most filament containing viruses are in the smallest aerosol fraction, this is an assumption at this point.

As discussed in response to comment no. 2 we have now included flow virometry measurements for the virus preparations and found that the percentage of filamentous versus spherical virions is consistent across batches (revised Fig. 1). This allows us to conclude on the shape distribution before aerosolization. However, the flow virometry method is currently not sensitive enough to quantify shape distribution in the samples recovered from the Andersen impactor (previously Fig. 3f; now Supp. Fig. 4b). We have reworded the text to make the reader aware of this limitation (lines 367–369).

22. Line 287- does low-speed centrifugation cause aggregation? This can be hard to test but can be mentioned in the discussion. Check reference – don't see that protocol in the cited paper.

This is an important point. To minimize potential aggregation after low-speed centrifugation, we applied sonication to ensure a homogeneous suspension before aliquoting the virus stocks. We also assessed the WSN-M1ud viral fractions by flow virometry across serial dilutions and observed that virion counts decreased linearly with dilution, while the median fluorescence intensity remained constant (Supplementary Fig. 2b–c). These results indicate that the preparation is monodisperse and that viral aggregates are largely absent [3, 4]. Sonication was also performed prior to each experiment. This is described in the methods section in lines 661–663.

23. Line 290- Suggest adding quantification to know the degree of enrichment and the starting shape distributions (see comment above re: Figure 1).

This overlaps with comments #2 and #4. Therefore, all details are given above.

24. Figure 5a-b- for the neutralization assay, the MOI was set to achieve 90% infection, which too high. This will allow for significant coinfection making it hard to have a linear outcome between cells being infected and the virus being neutralized.

We acknowledge that coinfection can occur at moderate MOIs and could, in theory, influence infection readouts. However, we would like to clarify that prior to the final experiment, we

conducted a pilot experiment using a range of MOIs (0.625, 1.25, 2, and 3) as shown in new supplementary Fig 5 to determine the conditions that meet the following criteria: (1) strong and comparable signal intensity between different virus preparations, (2) operation within the linear dynamic range of input virus versus infection output, and (3) avoidance of signal saturation.

Based on these pilot results, we selected an MOI of 1.25, which provided robust and reproducible infection levels across all virus conditions without saturating the assay. While some degree of coinfection is possible at this MOI, our experimental design focuses on comparing the relative neutralizing activity of different virus populations under identical assay conditions. All samples were treated equivalently, so any coinfection would occur uniformly across conditions and would not confound comparative results. Additionally, we consistently observed high reproducibility among technical triplicates within each biological replicate.

Most importantly, we detected a clear and substantial (~1 $\log_{10}$) difference in IC_{50} values between the filament-enriched and control virus fractions in mucus neutralization assays. This magnitude of difference strongly suggests that the enhanced resistance of filamentous virions is a dominant effect and unlikely to result from minor differences in coinfection frequency.

Reviewer #2 (Remarks to the Author):

The study is about influenza A virus, where one strain was modified to consist of both spherical and filamentous virus particles, and how the two different strains survive under various physicochemical pressures, i.e. high temperature, high salt, low pH and in aerosol phase. It is a nice study where the authors combined a variety of methods to explore virus inactivation in spherical and pleomorphic influenza, respectively. Aerosol experiments are lacking in literature, and the authors have created an impressive setup. The manuscript is well prepared with nice figures and text and I think the topic is very interesting, however, I have some comments that need to be addressed.

1. Considering the title, I find “airborne transmission” misleading. This is not a study where transmission is investigated. Your study evaluates virus survival and inactivation in an experimental setup.

With the previous title we aimed to combine the results from the aerosol experiments with the results from the primary airway cultures, the two parts that mimic the airborne phase and the infection of a new host during viral transmission. However, we acknowledge that we do not assess virus transmission. We have therefore adjusted the title. It now reads “Filamentous morphology of influenza A virus confers enhanced stability in aerosols”.

2. In general, I am not convinced by the authors’ introduction and discussion about the low pH of exhaled aerosol particles. My interpretation is that there are conflicting perspectives of the pH of exhaled aerosol particles, what particle sizes that are relevant and on what (relevant) time scales pH changes take place. Your manuscript does not represent (or cite) other work than your own in a time when the scientific community does not agree. Your model results from previously are not what I would consider established facts, and therefore I strongly suggest that you broaden the picture of pH importance for respiratory aerosol particles, citing other groups performing research on the same or similar topic. For example, this publication indicates that in indoor air, there is no strong chemical driver to make an aerosol droplet at pH 7 change level of pH in indoor air without

other strong sources: https://onlinelibrary.wiley.com/doi/full/10.1111/ina.12670 . Two of the co-authors of your manuscript have recently published an article showing that the pH increases shortly after exhalation, likely to pH ~10 (https://pubs.acs.org/doi/full/10.1021/acscentsci.5c00284). This is not clearly discussed in the present study, nor is a high pH evaluated in the experimental data.

We acknowledge that our original manuscript did not sufficiently represent the broader and ongoing scientific discussion regarding the physicochemical evolution of exhaled infectious respiratory particles (IRPs). As suggested, we have now expanded the introduction to provide a more comprehensive overview of current perspectives, citing additional studies from other groups (lines 82–89, 96-98). In this new text, we clarify that IRP pH dynamics depend on multiple factors, including particle size, matrix composition, timescale after exhalation, and indoor air chemistry, and that the scientific community has not yet reached a consensus.

To address the reviewer's point regarding rapid alkalinization after exhalation, we also conducted new experiments testing viral sensitivity to alkaline pH 10 in bulk solution (new Fig. 2f).

Finally, we added a discussion on the implications of both acidic and alkaline environments for IAV stability in IRPs in lines 542–559.

Introduction:

3. I find two important errors in the introductory paragraph about pH. Exhaled aerosol particles do not become alkaline because of HCO_3 uptake, it is due to CO_2 evaporation in ambient air, which has lower CO_2 concentration than the air in our lungs.

We acknowledge the mistake. We corrected the text accordingly in lines 93–94.

You write about "small particles" and "larger particles" as if they were defined; you need to be VERY specific here, because in aerosol science small particles can vary from 2 nm to 5 μm and large particles from 100 nm to 100 μm depending on whom you talk to.

We have now defined the size of IRPs as "submicron aerosol particles" in line 92 and "larger particles with an initial radius of 10 μm" in line 95–96.

4. During the covid pandemic, most exhaled viral RNA was found in particle sizes from 0.5-5 μm. These particles were likely around 2-20 μm as droplets. I would think that these are the important sizes to target for this type of research. Even though there might be viruses in even smaller particles <0.5 μm, these particles have limited ability to deposit in our respiratory system (ICRP model) and would therefore contribute with very little transmission of infection.

Would you please go through your text and verify that it is clear when you mean a wet aerosol particle (or a droplet) and when you mean a dry particle. I find it confusing sometimes when you refer to initial particle size - is that then the wet particle (droplet) size? Maybe there is nothing wrong, but I felt unsure in some places.

We thank the reviewer for this important point. In our aerosol chamber experiments, we report only the dry aerosol particle size distribution, as this is what we measured using a scanning mobility particle sizer (SMPS). These measurements consistently showed that the majority of dry particles are in the submicrometer range, and we have clarified this explicitly in the revised text (lines 537 and 544).

By contrast, whenever we refer to “initial particle size” elsewhere in the manuscript, this refers to the input conditions of our biophysical model, which simulates the physicochemical evolution of freshly exhaled infectious respiratory particles (IRPs). We do not specify “wet” in this context, because the term “initial” inherently refers to freshly exhaled, water-rich droplets at the moment of formation. The model assumes an initial temperature slightly below body temperature (36°C) and a particle water activity of 0.952 [5]. We have revised the manuscript to use the term “freshly exhaled” (lines 92 and 214) to make this distinction clearer.

Figure 1c:

5. The micrographs showing stained cells, could you indicate in the small headings above the pictures that what is shown are “cells infected with WNS/Udorn/WSN-M1ud” instead of just the virus strains (for clarity for readers who are not used to interpret this type if images, or those who see it in grayscale).

We have added headings to revised Fig 1c as suggested.

Figure 2

6. Do you need to have different types of y-axes for the different graphs? For the C/C0 you measured PFU/mL, the same as in graph a and b, so could you have the same for c-f?

To ensure consistency across panels, we revised the figures so that all bulk infectivity data now use a uniform y-axis, expressed as $\log_{10}$ infectivity loss normalized to the corresponding controls [$\log_{10}(C/C_0)$]. This normalization reduces the influence of variation in input virus titers and allows direct comparison across all conditions. We have updated Fig. 2 and Supplementary Fig.3 accordingly.

7. You use PBS as matrix for aerosolizing your virus – how is that related to your model results on pH for SLF?

The model results are most relevant for predicting pH evolution and to this aim, SLF was used a matrix for the model but also for the pH-related experiments in bulk and in the chamber. The switch to PBS was only applied for the salt-related experiments and consistent across these.

PBS contains only salts, and we used it as a simplified matrix to specifically study salt-mediated inactivation of IAV in aerosols. At 85% RH, which is above the efflorescence relative humidity (ERH), PBS aerosol particles remain aqueous, and the modeled particle pH is ~6.4, close to neutral.

At 25% RH, we assume that efflorescence occurs, consistent with the expected behavior of saline solutions. Because PBS provides a controlled and well-defined chemical system, it allows us to isolate the effect of salt-driven physicochemical changes without the additional complexity of SLF composition and buffering. This rationale is explained in the revised manuscript (lines 297-300).

Figure 3.

8. For the aerosol collection experiments, can you show the aerosol particle size distributions during each experiment? How stable is it within each collection time and between samples? You indicated earlier that you might have different protein concentrations in the liquids and this is something that could alter the size distribution and thereby what you collect, especially in the impactor. In any case, it is necessary to see how reproducible your aerosol concentration and

size distribution is during your sampling times across the full size range that you collect (5 nm – 10 µm). You also need to show the difference between the aerosol size distributions with PBS and SLF, respectively. If they differ, it is also natural that your results do so, and then that could explain parts of the differences you show.

One way to clearly show the expected variations due to variations in the aerosol concentration is to determine the theoretically collected aerosol mass or volume from aerosol sizing instrument data (for example SMPS and APS) for each sample. This could then be included in your uncertainty and statistical calculations.

We thank the reviewer for this helpful suggestion. We could not include SMPS measurements in each experiment due to limited availability of the respective instrumentation. However, we were able to include SMPS measurements for one set of experiments. These data from aerosolization of WSN and WSN-M1ud in the presence of 10 ppb HNO_3 are shown in the new supplementary Fig. 4a.

We found that the aerosol concentrations of WSN and WSN-M1ud varied across independent experiments (Supplementary Fig. 4a) but correlated with the genomic copy (GC) concentrations recovered from aerosol samples. Despite this variability, the decay kinetics from the two experiments were highly similar (Fig. 3e). These findings support the use of GC-normalized infectivity measurements for assessing IAV inactivation kinetics in this chamber system. We thus believe that this addition has strengthened our manuscript.

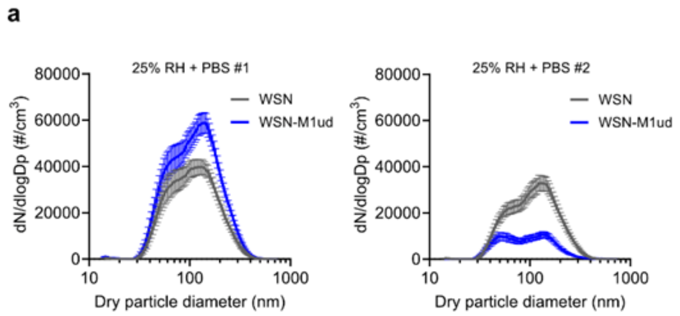

Supplementary Fig. 4. Size distributions of virus-laden aerosol particles at 25% RH. (**a**) Average (32 min) aerosol number particle size distributions (dN/dlogDp; cm^{-3}) obtained using a scanning mobility particle sizer (SMPS) (13.6–736.5 nm) from the experiments described in Fig. 3**e**. The SMPS scanned every 4 min for a total 32 min. Results from two biologically independent assays are shown in the left and right panel, respectively.

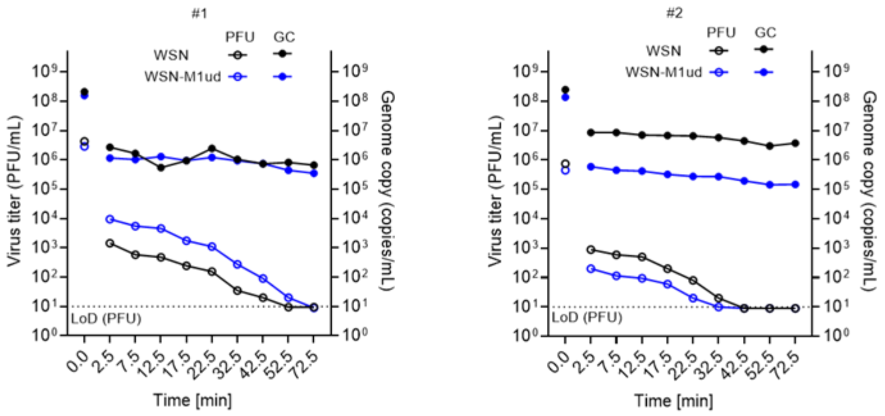

Raw IAV infectivity data from the aerosol chamber. Infectious virus titers (PFU) and total viral genome copies (GC) collected by BioSpot-VIVAS in two independent experiments at 25% RH in PBS.

9. Since you have minutes on the x-axes for graphs a-c, can you also have minutes on the y-exes for d-e? It would help to have the same scale on a-c. As for now, it is difficult to compare a with b and c.

As suggested, we have converted the time scales for all t_{99} values to minutes, ensuring a consistent unit across panels (revised Fig. 3).

10. About the time resolution on your x-axis. If you sample for 10 min, then maybe you cannot give a sample just one given time point on the x-axis on a scale of minutes. How did you sample at all the time points given in Supplementary Fig.2? Was each sampling point from a new aerosolization? What is meant by “one experiment”? How does one sample at, for example 2.5 min when sampling takes 10 min?

You write that “N0 is you first sampling time”. Does it take 10 min to collect that sample? Can you explain the jump from the first sample at time 0.0 (and how you sampled at t=0) to 2.5 min in Fig S2? I want to assure you that I very much appreciate that you include raw data like in S2, it is worth a lot for anyone who wants to compare! But maybe also make it shine through to Fig.3 as your divisions could look more representative of what they are, not starting from 0, but from -2 or what it actually is?

We appreciate the reviewer’s careful reading and have clarified the aerosolization, exposure, and sampling procedures in the Methods (lines 791–814) and in the legend of Fig. 3. Virus-laden aerosol particles were generated by a bubble-bursting nebulizer (Sparging Liquid Aerosol Generator, SLAG) for 1 or 1.5 min and then exposed in the aerosol chamber. Samples were collected in 5-min cycles: 4 min of collection followed by 1 min for Petri dish replacement. Accordingly, each data point reflects the median collection time of its interval. The first sample corresponds to 2.5 min after nebulization, which we now denote as $N_{2.5}$ to avoid implying sampling at t = 0.

It looks like there are error bars for some values but not for all?

Thank you for pointing this out. The error bars were mistakenly included for some technical replicates. Since technical replicates should not be presented with error bars, we have removed all error bars from these datasets and now show only the mean values.

Why are copy numbers at the same level for both strains in one experiment S2a and S2d but not in S2b and s2c? I mean, if you look at your PFU/M1ud/25% you intend to do the exact same thing twice and end up with samples at the level of 10^4 one time and 10^2 another – how do you explain that? Even though your inactivation curves look nice, if you get this much variation that could be just chance? My apologies for a dozen questions here but this is crucial information and not clearly described.

This is an important point. We consistently observe variability in both PFU/mL and GC/mL between independent chamber runs, reflecting at the first sampling point. While we cannot identify a single source, it likely reflects inherent run-to-run variability of the aerosol chamber rather than differences between viral strains.

This interpretation is supported by the SMPS measurements, which show that the total aerosol number concentration fluctuates between experiments (see also our response to comment #8). These variations in aerosol concentration appear to align with the GC levels recovered in the corresponding samples: runs with fewer aerosol particles tend to show lower GC counts and PFU titers. Thus, the initial PFU and GC levels could primarily depend on the aerosol concentration generated in each individual run.

To reduce the influence of these differences in initial aerosol loading, we normalized infectivity as $N(t)/N_{2.5}$, where $N_{2.5}$ is the PFU-to-particle ratio at the first sampling interval (0.5–4.5 min). This helps make the inactivation kinetics more comparable across runs.

11. In your previous publication in Frontiers, you have less than one order of magnitude inactivation within 45 min at low RH, here you have two orders of magnitude. Is it because of the different strains or what causes this difference?

In our opinion, this difference is likely linked to the use of two different strains. We think that this difference in airborne stability between the strains will be highly interesting to follow up on, but this is beyond the scope of the current study.

12. Your conclusion here (fig.3), in the title and in the study in general, IAV has an advantage from its two morphologies which endure either high salt or acid conditions better. But if it is so that the pH becomes low in the aerosol particle, the virus would be exposed to both low pH AND high salt conditions, and would it then be so sure that having two morphologies is an advantage? You state this message in the title as well, and I am not sure this is what you show with your data. If the spherical ones inactivate at low pH and the filamentous at high salt concentration, then they would all be inactivated by the time someone else inhales them. I feel like you argue against yourselves.

In response to a suggestion by reviewer 1 we have repeated the bulk and chamber experiments with the shape-enriched fractions of WSN-M1ud instead of the virus pair used previously to only assess shape-related differences. While using the shape-enriched fractions led to the same results in the salt-related experiments, we observed a clear difference for the low pH experiments: Filamentous virions displayed enhanced stability also for low pH conditions. As the shape-enriched fractions represent the better system due to the genetic identity of the fractions we focused the manuscript on these new results, and this point is no longer an issue.

Figure 4.

13. Impressive how well separated the spherical and filamentous viruses look from the electron microscopy images. Wouldn't it be good if you stated if it is TEM or SEM?

The images show representative slices through cryo-electron tomograms of WSN-M1ud fractions (new Fig. 1k). Shape quantification was performed on over 780 virions using cryo-TEM overview maps as well as flow virometry, as described in our response to Reviewer 1, point #4.

Figure 5.

14. What does MFI stand for in the figure text? It is nice when one can understand as much as possible when looking at the graphs without reading the text – would it be possible to indicate in each graph with dilution factor (also fig.4) what was diluted (mucus, HA)? Some graphs have several markers for each x-value and some have one x-value with error bars – can you do the same? How many replicates do you have for the ones with error bars?

We have now defined MFI (mean fluorescence intensity) in the legend of Fig. 4.

In addition, we have specified the neutralization matrices used (e.g., mucus or HA dilution) directly in each relevant panel of the revised Fig. 4 and Fig. 5 to improve readability.

Regarding the graphical representation, antibody-neutralization assays are typically presented as mean values with error bars together with the fitted curves to ensure clarity. In contrast, for virus growth curves it is standard practice in the field to display all biological replicates. We have also indicated the number of independent biological replicates performed in the Fig. 5 legend.

15. Did you ever try to aerosolize the two fractions SUP and PEL? I understand it is challenging to get enough material for it, but it feels like you could get more convincing data of any differences that exist.

This is a valid point and overlaps with Reviewer 1's comment #1. As detailed in our response there, we were able to obtain sufficient material to test both the WSN-M1ud SUP and PEL fractions in bulk experiments, as well as in the aerosol chamber experiments, shown in new Fig.1–3.

Minor comments:

16. Row 73: "Elevated acidity"? Isn't acidity enough?

According to the reviewer's suggestion here and comment no. 2, we have reworded this phrase to say "potential shifts in pH" instead of "elevated acidity" (line 77–78).

17. Row 74: Is there nobody else to cite but your own group regarding influenza in aerosol particles?

According to the reviewer's suggestion we added and cited more work from other groups (line 78).

18. Row 221: Elevated levels of proteins compared to what? And what is the total protein concentration?

Our initial aim was to explore whether additional factors, such as total protein levels in the purified virus stocks, might influence the interpretation of salt-mediated inactivation. As this was a minor point in the previous manuscript and large amounts of new data were added for this version we cut this part to keep the manuscript concise.

19. Several places: "an" MOI instead of "a".

This has been corrected (lines 667 and 843).

20. Row 382: what is the buffering capacity of PBS in this context?

We have added the catalog number for the PBS solution used in line 758.

21. Row 397: where would they be if not in the liquid phase of a droplet? Protruding out in the air?

In our opinion, protruding out of the droplet is a possibility but we revised the text to make it clear that this is highly speculative (l. 519-520).

22. Row 409: You write that you expect organic compounds to form a liquid coating around the salt particle – on what do you base this? From electron microscopy images with composition analysis that I have seen, there is no indication of this. There is no cited study either, is it just an assumption? It sounds wild to me.

Optical microscopy of an efflorescing SLF particle with a radius of ~40 µm from Luo et al. [5] showed rapid dendritic NaCl crystallization followed by a markedly slower coarsening phase, with crystal growth continuing for up to 2.5 h after initial crystallization. There is clear evidence that a liquid phase coexists with crystalline dendrites. These measurements were performed on micrometer-sized SLF particles; whether the same phase behavior extends to sub-micrometer dry aerosol particles remain to be tested. However, it is known that atmospheric particles observed in marine environments in the 1 µm size range exhibit often an inorganic core (effloresced salt) with a liquid organic coating, e.g. Fig 5(a) of Laskin et al [6]. Although these particles' composition is different from the SLF of our study, it is nevertheless plausible that the same causes (low surface tension of the organic phase, water transport through the particle surface) yield a morphology with the aqueous organic forming a distinct liquid phase being in contact with the gas phase and separated from the solid salt phase. We reworded accordingly in lines 531–540.

23. Row 598: missing punctuation

This has been corrected (line 799).

24.SLF recipe. There are different recipes for synthetic lung fluid. Can you motivate your choice?

The SLF recipe is now fully described in the methods section. We prepared SLF following the formulation adapted from Bicer [7], as detailed in Luo et al. [5], and freeze-dried it according to the procedure described by Hassoun et al. [8]. This SLF composition has been used in previous studies by Luo and Schaub et al. to study influenza A virus inactivation in aerosol particles or droplets under varying RH, pH, NaCl, and organic concentrations[5, 9]. These studies provide a solid foundation, and we therefore selected this recipe to ensure comparability with our published work on IAV aerosol stability.

25. In general, I find it hard to differentiate between the different lines and markers in the some of the graphs because the line goes through the hollow ones filling them. Could you choose other shapes to facilitate interpretation?

As suggested by the reviewer, we have adjusted the marker shapes for the different virus fractions in all relevant figures accordingly.

Reviewer #3 (Remarks to the Author):

I co-reviewed this manuscript with one of the reviewers who provided the listed reports. This is part of the Nature Communications initiative to facilitate training in peer review and to provide appropriate recognition for Early Career Researchers who co-review manuscripts.

References

1. Partlow EA, Jaeggi-Wong A, Planitzer SD, Berg N, Li Z, Ivanovic T. Influenza A virus rapidly adapts particle shape to environmental pressures. *Nature Microbiology* **10**, 784-794 (2025).

2. Fernandes C*, et al.* Flow virometry: recent advancements, best practices, and future frontiers. *Journal of Virology* **99**, e01717-01724 (2025).

3. Burnie J*, et al.* Flow Virometry Quantification of Host Proteins on the Surface of HIV-1 Pseudovirus Particles. *Viruses* **12**, (2020).

4. Maltseva M, Langlois M-A. Flow Virometry for Characterizing the Size, Concentration, and Surface Antigens of Viruses. *Current Protocols* **2**, e368 (2022).

5. Luo B*, et al.* Expiratory Aerosol pH: The Overlooked Driver of Airborne Virus Inactivation. *Environ Sci Technol* **57**, 486-497 (2023).

6. Laskin A*, et al.* Tropospheric chemistry of internally mixed sea salt and organic particles: Surprising reactivity of NaCl with weak organic acids. *Journal of Geophysical Research: Atmospheres* **117**, (2012).

7. Bicer EM. Compositional characterisation of human respiratory tract lining fluids for the design of disease specific simulants.). King's College London (2015).

8. Hassoun M, Royall PG, Parry M, Harvey RD, Forbes B. Design and development of a biorelevant simulated human lung fluid. *J Drug Deliv Sci Technol* **47**, 485-491 (2018).

9. Schaub A*, et al.* Impact of organic compounds on the stability of influenza A virus in deposited 1-µL droplets. *mSphere* **9**, e00414-00424 (2024).

__Liu et al., Response to reviewers' comments__

We would like to thank the reviewers for their thoughtful suggestions for further improvement. We have addressed the comments from the reviewers point-by-point as outlined below (in bold). The line numbers given below refer to the manuscript version with marked-up changes.

Reviewer #1 (Remarks to the Author):

This study addresses an important question about the functional role of influenza virus morphology in its persistence in the environment. The results are novel and have the potential to advance our understanding of virus transmission and adaptation. The revised manuscript is a substantial improvement from the previous version. The inclusion of the experiments with shape enriched virus populations of the same strain adds important rigor to the study. Additionally, assessment of virion shape by both flow virometry and electron microscopy provides greater confidence in the findings. Despite the key improvements, detailed below are several remaining comments regarding the morphological analyses, use of citations, and statements of conclusions.

General comments
1. Flow virometry clearly shows size difference between WSN and WSN-M1ud and size enrichment in different WSN-M1ud fractions. However, the details of the quantification do not seem convincing:
a. For many of the flow traces shown in figures S1 and S2, the virus populations are not clearly resolved from the noise. A subset of spherical virions is likely being excluded from the quantification; the problem is worse for some samples than others – in the unfractionated samples, the spherical peak is almost entirely gated out, and in the SUP fraction, it is sliced through the middle by the gate. The authors should address this limitation as it affects the value for the fraction of filaments (exaggerates their prevalence) and has a consequence on where the true filament gate likely lies.
As the reviewer mentions, the applied gating strategy could lead to a potential overestimation of filamentous particles in our preps. However, adjusting the gate in a way that includes more of the "missed" virions would come with the risk of including exosomes or virus particles with undetectable levels of HA incorporation. Given that such particles would not be infectious we believe that such a gating strategy would cause more problems than the one chosen. While we may overestimate the fraction of filamentous particles our results will still hold: We compare preparations with less filaments to preparations with more filaments for their aerosol stability; the absolute percentage of filamentous particles is not crucial. Moreover, comparison with EM suggests that the quantification of filaments is not biased towards filaments. We have now included a paragraph addressing this limitation in l. 669-678.

While flow data confirms that different fractions are enriched in different size particles, the quantification by flow is not of sufficient quality to quantitatively compare to EM. This makes EM analysis quite important and raw data should be provided.
Traditionally, virus shape has been quantified by EM. Thus, we understand the importance of including EM for comparing the results of flow virometry. Even though we

are not the first ones to employ flow virometry, and this method has been introduced and extensively validated for IAV in Partlow et al., Nat Microbiol. 2025 Mar;10(3):784-794, we still included a careful comparison of quantification by flow virometry versus EM. We find that they give highly comparable results and we have now included the underlying data to further corroborate this.

1) Representative EM maps have been included as new supplementary figure 3.

2) Additional EM images have been uploaded to figshare with the following identifiers (detailed in the data availability statement):
https://doi.org/10.6084/m9.figshare.31293784
https://doi.org/10.6084/m9.figshare.31293751
https://doi.org/10.6084/m9.figshare.31293721

3) Cryo-electron tomograms corresponding to Figure 1K were deposited into EMDB under accession codes: EMD-56668 (Unfractionated); EMD-56664 (SUP); EMD-56667 (PEL) (detailed in the data availability statement).

b. The current filament gating strategy assumes that WSN virions are entirely spherical, but data in Fig. 1d and S1 reveal two subpopulations, and S1 shows a diagonal tail radiating from the tight peak with greater fluorescence and scatter values. Since cutting off of a portion of the spherical peak due to poor separation from the noise (in S1) would increase the apparent fraction of larger particles (see preceding comment), the 'real' gate position might be after the main peak and before the small shoulder. Shifted gate would also make sense for the SUP data, which are quite different from the WSN virus – the small WSN 'shoulder' is a major subpopulation in the SUP, yet most of it is excluded from the current filamentous gate.
As stated in l. 151-152 we are fully aware that WSN does not only produce spherical virions: "In contrast, wildtype WSN produces predominantly spherical particles (> 80%), with the remainder consisting of shorter bacilliform virions.". We also make it clear in l. 185-186 that the SUP fraction does not only contain spherical virions: "while the supernatant (SUP) mainly consisted of spherical and bacilliform virions (Fig. 1h–k)". With our flow virometry approach we do not aim to say there are only two groups of virions. Instead, we wanted to establish a procedure that is faster and more amenable to medium-throughput for quantifying shapes in viral preparations compared to EM so that batch-to-batch variation can be assessed. To achieve this, we chose a gating strategy that distinguishes between spherical + short bacilliform shapes and longer filaments. We acknowledge that the different shapes could be distinguished into further subgroups, but the point here is to say that having a larger proportion of longer filaments helps with retaining virus infectivity under different stress conditions. We have checked the text, and we did not find places that would suggest that our WSN or SUP preparations only contain spherical particles.

2. Explain the protocol modification that was done to increase the fraction filamentous in the WSN-M1ud virus – is this the amicon concentration step mentioned in the methods? If so, it is unclear how passage through a 100kDa pore would selectively remove spheres. Additional methodological detail is needed to explain how this step results in filament enrichment.
For the experiments with the virus pair (WSN vs. WSN-M1ud) the virus preparations were concentrated by Amicon filtration (l. 587-591 in the methods section). In contrast, when performing fractionation of WSN-M1ud we followed the published protocol, which

includes ultracentrifugation as first step and does not include Amicon filtration (l. 592-603 of the methods section). It is important to note that the unfractionated preparation of WSN-M1ud also underwent ultracentrifugation. We thus speculate that the difference in filament content is due to this difference in concentration method. However, we have not tested this by performing both methods in parallel with the same virus stock and thus, we can only speculate that this is the reason for the difference in filament content. We have modified the methods section for the fractionation to make it clearer that the unfractionated WSN-M1ud also underwent ultracentrifugation (l. 587-596).

3. How does the dilution series test for aggregation? This approach may capture concentration-dependent aggregation, but it is unclear how it would distinguish irreversible aggregates from filaments.
It is correct that the dilution series captures mainly concentration-dependent aggregation. In the revised text, we now specify that the assay measures "concentration-dependent aggregation" (l.167, 171). If there was irreversible aggregation we would expect to detect this via EM. However, EM did not reveal the presence of aggregates, which we state in l. 179-180. Combined, these two findings (no concentration-dependent aggregates as measured by flow virometry and no aggregates detected by EM) suggest that aggregation is not a major problem.

4. Authors should include all EM images that went into quantification of morphology in figure 1 as raw data (currently only 1 image for the Unfractionated, SUP, and PEL are shown).
As described above, representative EM maps have been included as new supplementary figure 3, additional images have been uploaded to figshare and EM tomograms have been deposited in EMDB.

Minor comments
1. Some citations are incorrect or misrepresented and there is insufficient citing of previous literature:
a. Lines 104-106 -Make sure one of those references (12-14) includes ex vivo measurements to warrant the use of 'primary'.
Reference 12 (Seladi-Schulman et al.) includes data on a primary clinical virus isolate that was amplified only on airway cultures before being imaged in MDCK cells. We had referred to this finding and thus "primary" referred to the virus isolate, not the cell type. To avoid any confusion, we have removed "or primary clinical isolates" (l. 59).

b. Lines 392-394 – "Initial tests with our virus the virus pair WSN and WSN-M1ud showed only minor differences in sensitivity to a HA-binding neutralizing antibody (IgG) (Fig. 4a), despite previous reports suggesting filamentous particles have an advantage" – Please change how this is explained - these are genetic variants and do not contrast studies performed on shape-enriched particles.
We respectfully disagree with the reviewer. While reference 38 employs genetic variants the authors still conclude on the impact of shape on antibody sensitivity. More importantly, reference 37 uses shape-enriched fractions to test antibody sensitivity. Thus, we think that our explanation that the difference might be due to a lower proportion of filaments in our preparations is a reasonable hypothesis.

c. Lines 421-423 – "While viral NA activity correlates with mucus sensitivity, the association between viral shape and its ability to penetrate the mucus layer, along with the underlying

mechanisms, remains poorly understood" - should cite Vahey and Fletcher on mucus penetration and filaments, directional motion.

• Michael D Vahey, Daniel A Fletcher (2019) Influenza A virus surface proteins are organized to help penetrate host mucus eLife 8:e43764

We had already included this paper as a reference when describing what was known about the role of HA and NA in the movement through mucus (l. 116, 512). Thus, we had not ignored this important paper. With the sentence highlighted by the reviewer, we aim to say that the process is not fully understood. We do not refer to any specific finding and thus, we do not agree that this sentence needs a reference.

d. Lines 463-465 – "Given the higher increase in NA compared to HA these findings also support the concept that filamentous IAV with a higher NA:HA ratio may be more resistant to mucus inhibition" - seemingly contrary to what Vahey reported and should be discussed.

• Vahey, M. D. and D. A. Fletcher (2019). "Low-Fidelity Assembly of Influenza A Virus Promotes Escape from Host Cells." Cell 176(1-2): 281–294 e219.

The paper cited by the reviewer does not include any experiments with mucus. We thus do not see a discrepancy between our findings and the findings reported in Vahey and Fletcher.

e. Lines 484-485 – "filamentous morphology that is genetically controlled by the M segment" - genetic control is versatile and not limited to M.

We have reworded to say "filamentous morphology has been correlated…" (l. 431-432).

2. Lines 406-407 – "we also tested whether the reduced sensitivity of the filament-enriched fraction extends to IgA" - worth commenting that in the IgG experiment, sup is more sensitive and in the IgA experiment, pellet is more resistant (i.e. unfractionated tracks with the PELLET and SUP, respectively).

We have added a sentence in l. 371-373 to incorporate this observation: "Of note, the unfractionated virus preparation showed comparable IgG sensitivity to the PEL fraction but was closer to the SUP fraction in the IgA experiment."

3. Please tone down overstating conclusions

a. Lines 566-568 – "Our findings support and extend this concept by demonstrating that the higher HA protein content of filamentous virus particles confers a distinct infectivity advantage in the presence of neutralizing IgG and, particularly, IgA antibodies" -- HA content as the cause was not explored - that seems likely, but it is not addressed by the current analyses.

We have reworded to say "Our findings support and extend this concept by demonstrating that the filamentous fraction with the higher HA protein content has a distinct infectivity advantage in the presence of neutralizing IgG and, particularly, IgA antibodies." (l. 503-504).

b. Line 576 – "Together, our data provide a mechanistic understanding" – please tone down, current analyses do not address the mechanism though the findings are nonetheless relevant.

We have reworded to say "Together, our data suggest that differences in HA/NA content and ratio may underlie IAV shape-dependent infectivity during airborne transmission." (l. 512-513).

Reviewer #2 (Remarks to the Author):

Thank you very much for the revised manuscript. You have made impressive improvements with the additional experiments with SUP and PEL fractions investigated separately. Figures are much improved, as is the title.
The majority of my comments have been addressed and revised, however a few things remain. The major thing is about the acidity and particle size.
First, I find it unwise to use the term IRP (infectious respiratory particle) for anything else than the definition that is stated in the WHO document where it was recently defined (if you want to use the term). It is droplets from the respiratory tract containing pathogens and they can range from sub-micrometer to millimeter sizes. Thus, you cannot say that IRPs are the sub-μm and ”large particles” is another size fraction. IRPs are all sizes.
We fully agree with the reviewer that IRPs refer to all sizes, and we do not understand how the reviewer came to the conclusion that we use the term IRPs only for small sizes. In the introduction we describe the different sizes and how size impacts the microenvironment, and we do not state anywhere that IRPs only refer to submicrometer particles. We can only assume that the reviewer got this impression because our aerosol system has a strong bias to submicrometer particles. We have reworded key sentences in the text to clarify that our experimental system produces aerosol particles with a diameter of <7 μm in dry size and that this is the reason for our focus on the smaller size aerosols: l. 36-37, 81-102, 125-126, 267-272.
Moreover, we have added a paragraph to say that our experimental focus on small IRPs is a limitation of this work, and future approaches should also include systems to produce and capture larger IRPs (l. 524-529).

Then we come to the size versus acidification in aerosol phase. You now state clearly at what time scales that particles of various sizes become acidic (pH 4), which I highly appreciate. However, if you have a 1 μm particle in initial/freshly exhaled size, it will become around 0.3 μm when evaporated at RH <75% in a few milliseconds. These aerosol particles have the size corresponding to the deposition minima in the respiratory tract, i.e. only about 10% will deposit (and mainly in the alveolar region). Most importantly, these particles contain 1000 times less volume than 3 μm particles that deposit to >95% in the respiratory tract. This means that you need to have way more (x10^4) particles of those sizes to actually make a difference.
The other size that you mention, 10 μm, should take days to become as acidic (pH 4) according to your model. The 10 μm initial droplet will dry to around 3 μm and carries a lot of volume (potential to carry more virus). According to your own experiments, amount of aerosol corresponds with virus genome copies, i.e. it is quite likely homogenously distributed in your aerosol particles (unless you would show something else). There might be a non-homogeneous distribution of virus in exhaled aerosol from humans, but in nebulized aerosol, it is most likely that it is homogenously distributed. Therefore, you should also have virus in particles in the μm-size range.
What I'm trying to get to, is that your focus on sub-μm particles and your model showing that they become very acidic very fast is not the full picture of relevance for aerosol transmission of virus. I believe that you have at 30% of mass above 0.8 μm that you do not measure. You might be theoretically right, but in this context, it is not fully relevant to focus only on 0.3 μm particles and ignore the larger ones that you choose not to measure.

You only plot your aerosol number distribution and not the mass or volume distribution (Suppl.fig.4a), and for homogenously distributed virus in particles, the volume distribution would be more accurate to show. Also, measuring only with the SMPS, that is limited to particles <0.8 µm and then stating that the majority of particles were of sub-µm sizes is a bit ridiculous. Fine, there are by number less particles above 0.8 µm, but they matter for the mass that you collect and for the particles that contain the virus that you present as your result (PFU). This you can clearly see in your collection with the Andersen impactor. You have a lot of viruses in the size range from 0.65-5 µm as you show here.
To conclude this big comment, I think that you should reframe the way you talk about particles of different sizes, about the rate of acidification in the context of virus-containing particles (to align with your experiment), and the relevance of acidification in normal indoor air.
The reviewer is correct that the SMPS cannot measure particles > 800nm and our size measurements therefore did not show the full range of particle sizes emitted. To improve this aspect, we have now included OPC measurements. We have combined these data with the SMPS data to illustrate the particle sizes in our system better. This new graph is included as new Supp. Fig. 5a and shows that we indeed have particles with a diameter of up to 7 µm in dry size as pointed out by the reviewer. However, they are 10^4-10^5-fold lower in abundance than the particles with a diameter of 500nm or less in dry size. If we assume even, volume-proportional distribution of viruses the vast majority of viruses will be in the submicrometer particles (new Supp. Fig. 5b). We have changed the text to say that we have particles up to 7 µm in dry size but that the focus is on submicrometer particles as they hold the majority of viruses in this system (l. 268-272).

We would also like to mention that we do not find it appropriate that the reviewer describes our conclusion (or any other part of our work) as "ridiculous". More appropriate wording would have been appreciated.

And if you want to say that aerosol particles become acidified when you expose them to HNO3, can you calculate the expected pH for the particle sizes corresponding to the Andersen impactor sizes as well?
We had previously published the results of pH modelling for a number of particle sizes and thus had not included the modeling results again here. However, we understand that it is helpful to have the modeling results included and thus we have added pH calculations for particles ranging from an initial wet size of 1-20 µm radius. These results are shown in Supp. Fig. 5d and described in l. 308-314.

Another thing on the topic. If the filamentous IAV are as long as 10 µm, how do they fit in the sub-µm particles? Do they wind up or shrink in some way?
At this point, we do not know the morphology of the filamentous virions in submicrometer particles. We can only speculate that they "roll up", which could potentially provide protection for some of the surface proteins.

Then there's the other thing about the SMPS measurements. The inter-run variability seems a bit high, varying a factor of 5 from #1 and #2 that you show in S.4a. Combined with the fact that you don't measure any particles larger than 0.8 µm, I don't think you can say that there's a distinct pattern difference in the Andersen impactor results, which I understand it as you ran it once for each virus strain. Limited aerosol particle monitoring during your experiments is a major flaw of the aerosol part of this study, especially since the data you show indicate quite some variability.

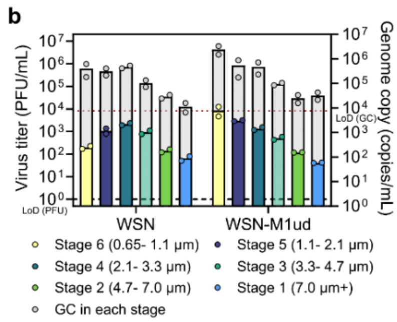

As stated in the legend of the previous supp. fig. 5b (now supp. fig. 5e) the experiments with the Andersen impactor were run twice independently and the results from both experiments match very well. As shown in the figure, the genome copies and the infectious titers were highly comparable across the two experiments represented by the two dots for each bar. Given this robustness of the data, we respectfully disagree with the reviewer and believe that we can use the data to conclude that the two viruses show a distinct pattern in the Andersen impactor.

Minor comments

Row 44. Include statistics on this correlation.

We had already included statistical testing in figures 4-5 that show the relevant data. We thus assume that the reviewer's suggestion refers to mentioning that the inhibition is statistically significant in the abstract and have thus reworded the abstract in l. 44 as follows: "… we further found that filamentous IAVs possess a <u>statistically significant</u> infectivity advantage under mucosal immune pressures, including neutralizing antibodies, and mucus inhibition."

Row 46. How acidified are particles of relevant particle size?

As described above we have now included pH calculations for different aerosol particle sizes. Please see our explanations above.

Row 74. Why did you choose reference no.23? Why not cite the actual original article that shows the data and science and not the bench version.

The statement "IAV can spread through infectious respiratory particles (IRPs) emitted from the respiratory tract of an infected individual during coughing, sneezing, breathing and talking (ref. 23)." is rather general and does not refer to the results of a single paper but rather a body of work. We thus believe that citing a review summarizing knowledge on this topic is an appropriate reference.

Row 365-367. As written above, this is a too strong statement considering the data you show.

We reworded the text (l. 333-337). It now reads "We observed that viral genome content and infectivity declined from stage 6 to stage 1 for WSN-M1ud viruses, whereas genome content and infectivity peaked in stage 4 for WSN virions, a result contrary to our initial expectation."

Row 372. I don't agree that you expose aerosol to low pH until you have calculated the pH for Andersen-impactor-sizes.

Row 523-526. Again, this is theory but can you do the model calculations for relevant sizes and say the same?

As described above we have now included pH calculations for different aerosol particle sizes and these calculations show that the majority of viruses will be exposed to inactivating pH values.

Row 534. At RH below efflorescence, there can be a liquid phase from organics, but there shouldn't be any aqueous phase, right?

The reviewer is correct: It should say “liquid microenvironment”, not “aqueous microenvironment”. This has been corrected (l. 475).

Row 542-545. It doesn’t have to. This depends very much on the time scale that is relevant. So again, back to calculating the actual pH that is reached for 0.6-5 μm sizes and include those. **As described above we have now included pH calculations for different aerosol particle sizes and these calculations show that the majority of viruses will be exposed to inactivating pH values.**

__**Liu et al., Response to reviewers' comments**__

We would like to thank the reviewers for their final remarks. We have addressed the comments from the reviewers point-by-point as outlined below (in bold).

REVIEWERS' COMMENTS

Reviewer #1 (Remarks to the Author):

Our positive overall assessment of the study and its impact we communicated previously stands. We further acknowledge the improvements in this round of revisions, but still have one major concern when it comes to the flow virometry and its interpretation. Basically, quantitative data for particle shape cannot be extracted from the flow data shown in Figure S2 and corresponding to the panels i, j, and l in Figure 1. Data presented in Figure S1 and corresponding to the panels e and f in Figure 1 do not suffer from the same shortcoming and seems fine to include. At most, the data shown in Fig. S2 support a qualitative conclusion of filament enrichment in the pellet fraction. Together with the provided EM data, it is sufficient to make the authors' claims as relevant for this study, but I would refrain from providing quantification or quantitative comparisons to the EM data. We suggest that the authors remove panels of Fig1. i, j, and the virometry quantification from panel l. We provide detailed explanation for this suggestion below.

The simple application of the same gating strategy across samples in Figure S2 where spherical population is not sufficiently pulling out of the noise definitely leads to an overestimation of filaments (and not only potentially, as indicated in line 671 of the revised manuscript). This is not to suggest that a different gate should be drawn because drawing it any lower would include noise (and lead to underestimating filaments), but to emphasize the limitation of those specific virometry measurements (Fig. S2) to derive quantitative value for shape distributions. A close look at the scatter plots shown in Fig. S2, especially for the unfractionated and the Sup fraction, reveals a virus subpopulation that is present but not entirely pulling out of the noise. This is not as evident in the pellet fraction, possibly because the signal is dominated by larger filaments. The lower relative inclusion of the spherical particle data can also explain the distributions shown in Fig. 1h where the virus peak for the unfractionated and supernatant fractions is shifted towards larger events and does not correspond with the sphere peak in WSN or WSN-UdM1 samples. Given these limitations, we do not think that quantitative values could be derived from the flow virometry data in Fig. S2.

Along the same lines, direct quantitative comparisons between flow virometry and EM are not appropriate in the context of Fig. S2 data. The claim that the missed virions have undetectable HA levels and thus are not infectious and can be disregarded is an assumption that is not substantiated by evidence. Smaller particles not pulling out from the noise could result from a technical issue like insufficient labeling antibody, higher noise level, fluorescent laser instability, trigger delay issues, etc. With respect to the virus structure, it could reflect epitope accessibility and not necessarily HA levels. Even if HA levels were lower on a subset of particles, sensitivity by virometry is not a measure of particle infectivity, and disregarding particles not revealed by virometry as not infectious is unjustified. Importantly, these missed virions are being counted in the

EM quantification, so obtaining the same values is not expected, and rather than corroborating virometry, it further brings into question the gating strategy.

In conclusion, the authors should acknowledge that flow virometry is a quick readout that allows quick corroboration of filament enrichment in the pellet fraction, but in the case of provided data in Fig. S2, not a quantitative assessment since it excludes a major virus subpopulation.

Flow virometry has been published as a quantitative method to measure influenza virus shape: https://www.nature.com/articles/s41564-025-01925-9
We used this method developed by others and we carefully compared results obtained by flow virometry with results obtained by the standard methodology electron microscopy. This resulted in us obtaining the same proportions of spherical versus filamentous viruses. In our opinion this further validates flow virometry as a quantitative method.

As the reviewer agrees that "together with the provided EM data, it is sufficient to make the authors' claims as relevant for this study" we believe that the current presentation of data and their interpretation is appropriate for our study. We therefore did not remove the data shown in figures 1i-j, l.

Reviewer #2 (Remarks to the Author):

Great that you have added the volume distribution of your aerosol. Could you change to diameter instead of radius to be in line with your previous plots?

The graphs depicting the volume distribution of our aerosol are already plotted as diameter and not radius. Please see supp. figures 5a-c.

Highly appreciate the model figures being included now, it is more transparent to the reader. Although I am not convinced that this plays a major role for airborne transmission of influenza (considering the low lung deposition fraction of 0.3 μm particles).

We are pleased to read that the reviewer finds our addition of the modelling results helpful.

Regardging the Andersen impactor measuremetns, did you not have a filter collecting the <0.65 μm particles? That would have shown real proof of your virus distribution.

The Andersen impactor that we used comes with a fixed size resolution and the smallest sizes collected are as indicated in supp. fig. 5e particles between 0.65-1.1 μm. It was not possible to collect particles < 0.65 μm. Given that this experiment was not performed to support the main conclusions of the manuscript but is rather an observation on the side, further tests would be beyond the scope of this manuscript.
